# Regulation of microtubule dynamics and function in living cells *via* cucurbit[7]uril host–guest assembly†

**DOI:** 10.1039/d4sc00204k

**Published:** 2024-06-20

**Authors:** Akshay Saroha, Monica Swetha Bosco, Sneha Menon, Pratibha Kumari, Tanmoy Maity, Subinoy Rana, Sachin Kotak, Jagannath Mondal, Sarit S. Agasti

**Affiliations:** a New Chemistry Unit, Jawaharlal Nehru Centre for Advanced Scientific Research (JNCASR) Bangalore Karnataka 560064 India sagasti@jncasr.ac.in; b Tata Institute of Fundamental Research 36/P, Gopanpally Village Hyderabad 500046 India; c Materials Research Centre, Indian Institute of Science C. V. Raman Road Bangalore 560012 India; d Department of Microbiology and Cell Biology, Indian Institute of Science 560012 Bangalore India; e Chemistry & Physics of Materials Unit, Jawaharlal Nehru Centre for Advanced Scientific Research (JNCASR) Bangalore Karnataka 560064 India; f School of Advanced Materials (SAMat), Jawaharlal Nehru Centre for Advanced Scientific Research (JNCASR) Bangalore Karnataka 560064 India

## Abstract

Living systems utilize sophisticated biochemical regulators and various signal transduction mechanisms to program bio-molecular assemblies and their associated functions. Creating synthetic assemblies that can replicate the functional and signal–responsive properties of these regulators, while also interfacing with biomolecules, holds significant interest within the realms of supramolecular chemistry and chemical biology. This pursuit not only aids in understanding the fundamental design principles of life but also introduces novel capabilities that contribute to the advancements in medical and therapeutic research. In this study, we present a cucurbit[7]uril (CB[7]) host–guest system designed to regulate the dynamics and functions of microtubules (MTs) in living cells. To establish communication between MTs and CB[7] and to reversibly control MT function through host–guest recognition, we synthesized a two-faced docetaxel-*p*-xylenediamine (Xyl-DTX) derivative. While Xyl-DTX effectively stabilized polymerized MTs, inducing MT bundling and reducing dynamics in GFP-α-tubulin expressing cells, we observed a significant reduction in its MT-targeted activity upon threading with CB[7]. Leveraging the reversible nature of the host–guest complexation, we strategically reactivated the MT stabilizing effect by programming the guest displacement reaction from the CB[7]·Xyl-DTX complex using a suitable chemical signal, namely a high-affinity guest. This host–guest switch was further integrated into various guest activation networks, enabling ‘user-defined’ regulatory control over MT function. For instance, we demonstrated programmable control over MT function through an optical signal by interfacing it with a photochemical guest activation network. Finally, we showcased the versatility of this supramolecular system in nanotechnology-based therapeutic approaches, where a self-assembled nanoparticle system was employed to trigger the MT-targeted therapeutic effect from the CB[7]·Xyl-DTX complex.

## Abstract 2

We describe here a reconfigurable supramolecular system based on cucurbit[7]uril (CB[7]) host–guest chemistry to regulate the dynamics and functions of microtubules in living cells.

## Graphical abstract

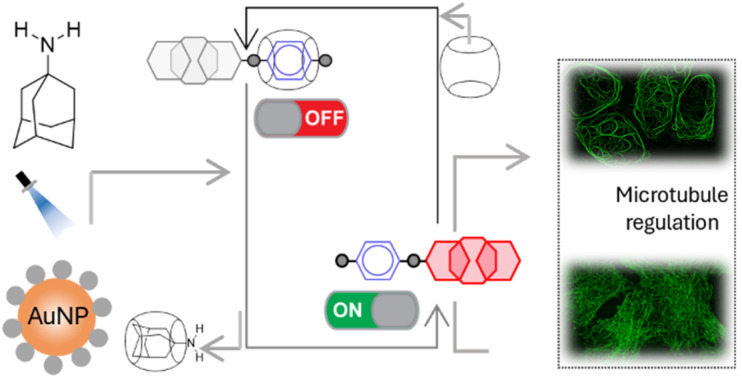

## Introduction

Microtubules (MTs) are ubiquitous cytoskeletal filaments in eukaryotic cells that are assembled from the dynamic polymerization of globular α/β heterodimers. Along with actin and intermediate filaments, MTs are responsible for providing most of the structure and spatial organization in the cell. Apart from establishing and maintaining cell morphology, MTs play critical roles in multiple cellular processes, including intracellular trafficking, cell polarization, migration, and cell division.^1^ The underlying dynamics of the MTs play a pivotal role in many of these microtubule-dependent processes; perhaps the most striking example is mitosis, where dynamic assembly and disassembly are of paramount importance in segregating and separating the chromosomes.^2^ To control the dynamics, assembly, and function of MTs, living cells utilize a variety of sophisticated biochemical regulators and signal transduction mechanisms in a way that is appropriate to bring about a specific functional outcome.^1,3–5^ Replicating the functional aspects of these complex regulators with simple synthetic building blocks having signal–responsive properties represents a major focus of interest in modern-day chemical biology research.^6–13^ These synthetic systems have the potential to provide programmable and ‘user-defined’ control over the underlying dynamic assemblies and associated cellular functions of MTs for a diverse range of fundamental studies as well as biomedical applications. In addition, these minimalistic synthetic modules can be eventually integrated into multifunctional life-like systems with increasing complexity, bringing us closer to the vision of building a synthetic cell from the bottom up. Inspired by the molecular interactions that play a crucial role in underpinning the regulatory networks in cells, chemists have created a few interesting synthetic supramolecular scaffolds with attractive molecular recognition properties.^14^ These supramolecular scaffolds exploit the principles of non-covalent interactions to open new opportunities for the rational design of synthetic assemblies that can potentially emulate the function of these biological regulators.^15,16^ However, designing a supramolecular assembly that can be programmed to work under living cellular conditions poses a daunting challenge, as traditional supramolecular scaffolds lack the affinity and selectivity that are required for them to function in cellular complexities.

In this context, host–guest systems based on synthetic macrocyclic receptors provide a viable starting point in this direction, as they are capable of displaying specific molecular recognition properties in the aqueous media. Among various macrocyclic receptors, CB[7], the heptameric member of the cucurbit[*n*]uril family, emerged as one of the prime candidates for constructing supramolecular assemblies with potential functionality in living systems due to its high water solubility (20–30 mM in pure water), biocompatibility, and remarkable ability to form ultra-high affinity (*K*_a_ > 10^6^ M^−1^ and up to 10^17^ M^−1^) and selective mono-valent (1 : 1) host–guest inclusion complexes in biological complexities.^17–26^ The affinities and selectivity that CB[7] displays toward certain synthetic guest molecules match with those typically found in the interaction of proteins and antibodies toward their cognate ligands. The high binding affinities of CB[7]·guest complexes facilitate the expansion of host–guest chemistry to dilute and complex environments, which is typically the case in biological settings.^27–39^ In addition, the emergence of high-affinity recognition from monovalent complexation decreases the need to exploit the structural and combinatorial complexity of multivalency, thereby providing a straightforward design approach for developing reconfigurable multi-component systems. In recent years, these properties have been strategically exploited to design high-fidelity biomimetic systems for the regulation of proteins/enzymes, nanozymes, supramolecular antibiotics, and platinum-based drugs.^40–49^ For example, Isaacs group has elegantly utilized the ultra-high binding affinities, commonly exhibited by CB[7]·guest complexes, to out-compete bovine carbonic anhydrase enzyme for their targets (inhibitor), thereby gaining control over their cognate enzymatic function.^40^ In a different approach, Liu group demonstrated on-demand control of the protein's function by site-specific mutation of amino acid residue in proximity to a protein's substrate entry site with an unnatural amino acid bearing a CB[7] specific guest side chain.^41^ Zhou and co-workers demonstrated reversible 5-formylcytosine (5fC)-targeted intervention tools to control the enzymatic recognition process at 5fC sites by manipulating host–guest interactions based on CB[7].^42^ Recently, Rotello group reported the fabrication of bioorthogonal nanozymes through the encapsulation of transition metal catalysts into the monolayer of protein-sized gold nanoparticles and its allosteric regulation *via* CB[7] host–guest chemistry.^43^ Kim, Isaacs, Zhang, and others reported the encapsulation of platinum-based drugs inside CB[7] for its protection against biological nucleophiles and its target-specific activation in cancer cells.^44–49^ While these examples exquisitely demonstrate the importance of CB[7] host–guest recognition to achieve biomimetic regulatory assemblies, it has so far been largely unexplored in the context of gaining a synthetic control over the dynamic assemblies and associated cellular functions of MT, which is a highly critical cytoskeleton component of a living system.

**Scheme 1:**
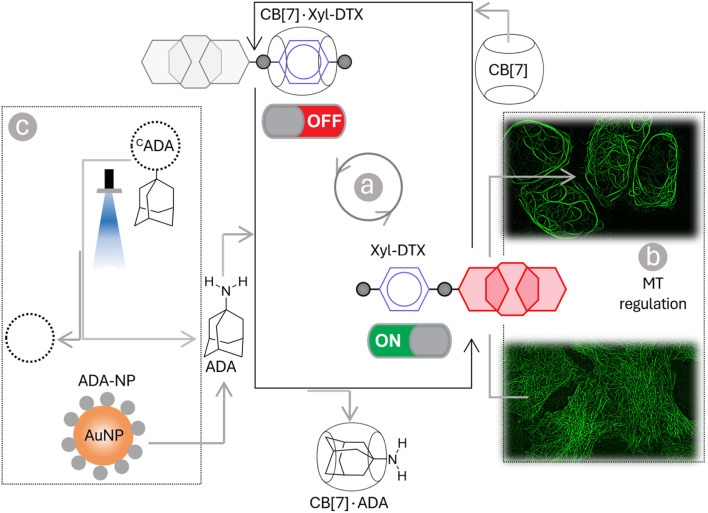
Schematic representation showing programmable control of the MT function in living cells *via* host–guest chemistry. Threading and dethreading of Xyl-DTX using CB[7] host–guest chemistry (shown in a) was coupled with the MT function in living cell (shown in b). The CB[7] host–guest switch was further connected to various guest activation networks (shown in c) to achieve a ‘user-defined’ regulatory control over the MT function. Three types of signals—chemical signal (ADA), light signal (^C^ADA), and nanoparticle signal (ADA-NP)—are employed for this control.

In this article, we describe a supramolecular strategy to regulate the dynamics and functions of MTs in living cells by interfacing with CB[7] mediated host–guest chemistry (Scheme 1). Further, we demonstrate an optical regulation of MTs through an interconnected regulatory network that provides guest activation under photochemically controlled conditions. In order to establish a communication between CB[7] and MTs, we used an MT-binding and stabilizing agent, docetaxel (DTX), which is known to effectively bind polymerized MTs to reduce MT dynamicity and promote mitotic arrest.^5,50^ Towards this end, we have engineered the DTX molecule and incorporated a *p*-xylenediamine (Xyl) moiety into its structure to achieve a two-faced docetaxel derivative (Xyl-DTX) that combines an MT binding epitope (DTX) with a CB[7] recognition moiety (Xyl). *In vitro* polymerization assays and cellular studies using GFP-α-tubulin-expressing HeLa cells demonstrated that Xyl-DTX effectively stabilizes polymerized MTs, leading to MT bundling and mitotic cell arrest. However, interestingly, we found that the CB[7]·Xyl-DTX complex has a significantly reduced effect on MTs, both *in vitro* and in cells, which was also supported by molecular dynamics (MD) simulation-based interactions mapping. This window of differential activity between Xyl-DTX and CB[7]·Xyl-DTX complex allowed us to regulate MT activity by recognition-mediated disassembly of the host–guest complex. We disassembled the CB[7]·Xyl-DTX complex by displacing the Xyl guest from the CB[7] cavity with 1-adamantylamine (ADA), a higher affinity guest of CB[7]. We found a concomitant restoration of MT stabilization activity from fluorescence time-lapse microscopy studies in live GFP-α-tubulin expressing HeLa cells with the ADA mediated displacement of Xyl-DTX from CB[7] cavity, indicating a host–guest mediated control over the MT function. Further, to gain optical control over the MT function, we used ADA conjugated to a photocleavable group (^C^ADA), which, upon light irradiation, releases ADA and triggers activity from the CB[7]·Xyl-DTX complex. Finally, to demonstrate an immediate therapeutic implication of this strategy, we showed ADA-decorated gold nanoparticle (ADA-NP) mediated activation of therapeutic effect from CB[7]·Xyl-DTX complex. To the best of our knowledge, this study marks the first instance where host–guest recognition of CB[7] has been employed to exert synthetic control over the dynamic assemblies and functions of MTs in living cells. The only other study that has employed a member of the cucurbit[*n*]uril family, specifically CB[8], focused on intertubular cross-linking, resulting in the conversion of MTs from fibrous structures into nanoparticle aggregates.^9^ In contrast, our study unveils a new mechanism for host–guest-mediated regulation of MT dynamics and function in living cells. We have harnessed CB[7] host–guest recognition to directly interact with MT lattice stabilization, effectively achieving substantial control over the structure, dynamics, and function of MTs. The high-affinity recognition of CB[7] has been particularly effective when interfacing with highly potent DTX-derived compounds (with IC_50_ values in the μM range or below). The high-affinity binding of CB[7] ensures the formation of regulatory host–guest complexes at low μM concentrations, effectively suppressing or activating the function of DTX derivative under ‘user-defined’ conditions. We further showed that the versatility of this approach extends beyond the chemical realm and includes MT regulation *via* light and nanoparticle signals. Furthermore, the generalizability of this strategy paves the way for the potential regulation of other highly potent toxins to modulate protein function or for therapeutic purposes.

## Results and discussion

**Scheme 2:**
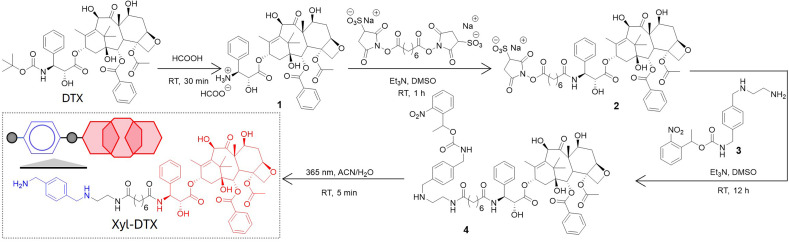
Synthetic strategy for derivatization of docetaxel (DTX) with *p*-xylenediamine (Xyl) to achieve a two-faced docetaxel derivative (Xyl-DTX) that combines an MT binding epitope (DTX) with a CB[7] recognition moiety (Xyl). RT: Room Temperature.

We derivatized the DTX molecule with Xyl at the 3′-amine substituted side chain. This site is known to border along the bound tubulin surface yet projected away from the interior binding site, thereby enabling the retention of potency upon derivatization. We have synthesized the Xyl derivative of DTX in a two-step conjugation process using a bivalent *N*-hydroxy succinimidyl linker (bis(sulfosuccinimidyl) suberate, BS_3_). Scheme 2 presents the synthetic strategy, and ESI† details the synthesis protocol. In brief, first, the 3′-*tert*-butyl group of commercially available DTX was deprotected by formic acid to obtain its amine derivative (1). Under basic conditions, the deprotected NH_2_ group of 1 was coupled with BS_3_*via* amine-NHS coupling. The docetaxel-BS_3_ (DTX-BS_3_, 2) conjugate thus formed was purified by HPLC and subsequently used for conjugation with *o*-nitrobenzyl protected Xyl guest molecule (Xyl-EDA-PC, 3). The covalently conjugated Xyl-DTX-PC (4) was subjected to UV irradiation (365 nm, 50 mW cm^−2^, 5 min) to cleave the photolabile *o*-nitrobenzyl protecting group. The photocleaved reaction mixture was purified to obtain the final two-faced docetaxel Xyl conjugate (Xyl-DTX), which was further characterized by NMR and HRMS (see ESI†). The purity of the final Xyl-DTX conjugate was confirmed through high-performance liquid chromatography (HPLC) analysis (ESI Fig. S7†).

**Fig. 1:**
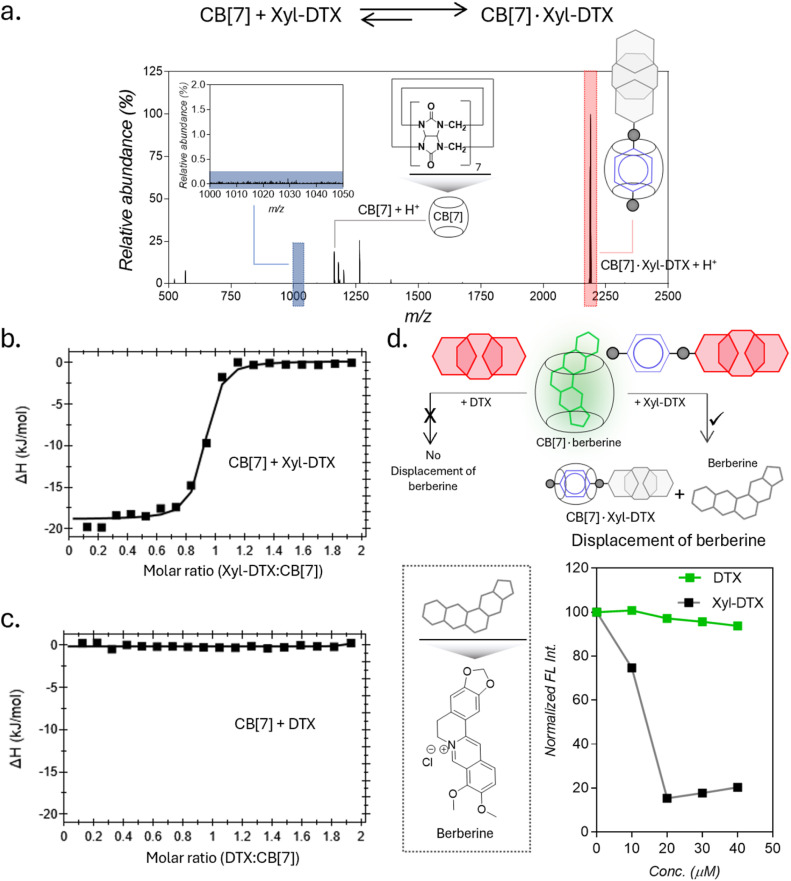
Complexation studies of Xyl-DTX with CB[7] in water and in PBS. (a) Positive MALDI-MS spectrum of a mixture of Xyl-DTX and CB[7] showing signature of CB[7]·Xyl-DTX complex formation. Calculated *m*/*z* for CB[7] + H^+^: 1163.35, found: 1163.17; calculated *m*/*z* for CB[7]·Xyl-DTX + H^+^: 2188.86, found: 2188.58 (b and c) ITC analysis for the complexation of CB[7] with (b) Xyl-DTX and (c) DTX. (d) Schematic showing displacement assays with CB[7]·berberine to compare binding interaction of Xyl-DTX and DTX with CB[7]. Fluorescence-based titration studies show a decrease in fluorescence when Xyl-DTX is added to the CB[7]·berberine complex (prepared by mixing 25 μM CB[7] with 5 μM berberine) in PBS, indicating the displacement of berberine. In contrast, the addition of DTX to the CB[7]·berberine complex does not change the fluorescence, indicating no displacement of berberine.

To investigate a supramolecular host–guest complex formation between the synthesized Xyl-DTX derivative and CB[7], we carried out matrix-assisted laser desorption ionization mass spectrometry (MALDI-MS) analysis. We first analyzed Xyl-DTX using an α-cyano-4-hydroxycinnamic acid (CHCA) matrix and found its characteristic mass signature at 1025.51 *m*/*z* (ESI Fig. 8†). In order to determine the supramolecular assembly characteristics, we initiated a complexation reaction by mixing 10 μM of Xyl-DTX with 1 eq. of CB[7] in an aqueous solution for 1 h. The MALDI-MS spectra of this mixture displayed an intense mass signature at 2188.58 *m*/*z*, corresponding to the 1 : 1 host–guest complex between CB[7] and Xyl-DTX (Fig. 1a). We next used isothermal titration calorimetry (ITC) to determine thermodynamic parameters for this complexation process. Along with the Xyl guest-modified drug (Xyl-DTX), we also studied the parent DTX molecule for its affinity toward CB[7]. The ITC studies were performed at 25 °C by titrating Xyl-DTX or DTX solutions into the CB[7] solution. Xyl-DTX showed negative enthalpy change (Δ*H*) with a binding stoichiometry close to 1 : 1 (Fig. 1b). The binding constant determined from the curve-fitting analysis revealed an association constant (*K*_a_) of 1.70 × 10^7^ M^−1^ between Xyl-DTX and CB[7]. However, as shown in Fig. 1c, when titration was performed with the parent DTX molecule into a solution of CB[7], no clear heat change was observed. These experiments suggest that the parent DTX molecule does not possess any significant affinity towards CB[7], and host–guest complexation in Xyl-DTX is primarily driven by an interaction between Xyl moiety and CB[7] host. We further used a solution-based fluorescence indicator displacement assay (IDA) to confirm the strong binding interaction between Xyl-DTX and CB[7]. We used berberine encapsulated CB[7] complex for the IDA, where berberine is known to display an enhanced emission upon encapsulation inside the cavity. We carried out the fluorescence titrations of CB[7]·berberine complex (*K*_a_ of 10^6^ M^−1^) with increasing concentrations of DTX and Xyl-DTX in a 96-well micro-plate.^21^ Upon titrating with DTX, we observed that there is a negligible decrease in fluorescence intensity, indicating the minimal displacement of berberine by DTX (Fig. 1d). However, in the case of the Xyl-DTX addition, a near quantitative reduction in fluorescence emission was obtained, indicating the complete displacement of CB[7]·berberine complex by relatively high-affinity Xyl-DTX. Overall, these results confirm the high affinity and chemoselective complexation of Xyl-DTX conjugate with CB[7] *via* host–guest interactions.

**Fig. 2:**
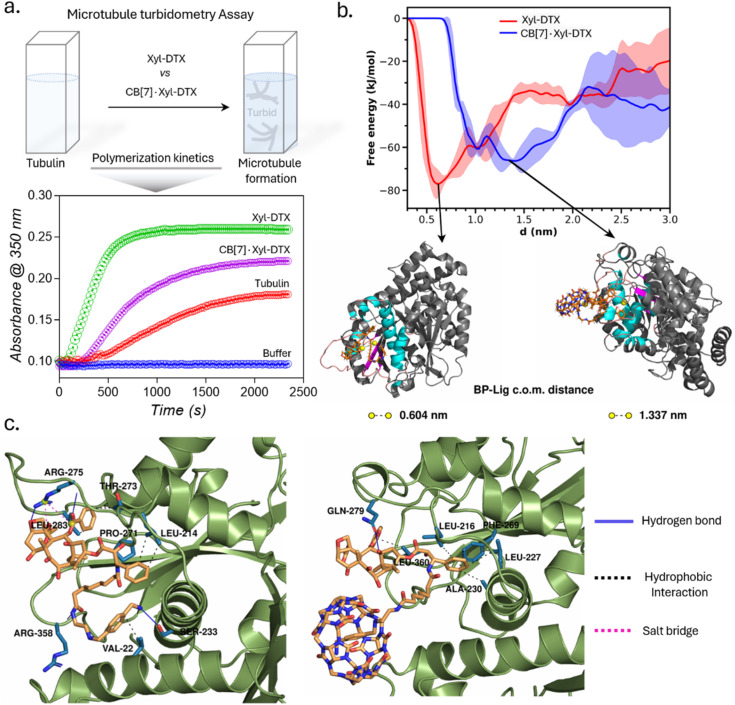
Microtubule turbidometry assay and molecular insights into the relative binding of Xyl-DTX and CB[7]·Xyl-DTX to tubulin *via* computational modeling. (a) Effect of Xyl-DTX and CB[7]·Xyl-DTX on the turbidity time course of tubulin polymerization. (b) Free energy profiles of binding of Xyl-DTX and CB[7]·Xyl-DTX complex to tubulin. Representative snapshots corresponding to the free energy minimum in each case are presented. The ligand molecules are represented as sticks. The protein binding pocket is colored according to secondary structure in cyan (helix), magenta (β-strands), and pink (coil); the rest of the protein is colored grey, (c) Protein–ligand interactions involved in the binding of Xyl-DTX (left) and CB[7]·Xyl-DTX (right) to tubulin. The protein residues involved in the interactions are shown as sticks colored in blue. The different types of interactions are depicted as solid and dashed lines, as indicated in the legend.

In order to understand the effect of CB[7] complexation on the tubulin polymerization efficacy of Xyl-DTX, we performed an *in vitro* turbidity assay.^51^ This assay monitored the polymerization kinetics of purified tubulin at 37 °C by spectroscopically measuring turbidity at 350 nm. As shown in Fig. 2a, in the absence of any DTX conjugates (taken as tubulin control), tubulin heterodimerizes and self-assembles to form microtubules in a time-dependent manner with the observation of lag, log, and steady-state phases. Notably, the addition of Xyl-DTX (10 μM) significantly enhanced the tubulin polymerization compared to the control, supporting the strong MT binding and stabilization ability of the Xyl-DTX conjugate.^52^ However, upon complexation with CB[7] (10 μM Xyl-DTX with 200 μM CB[7]), Xyl-DTX substantially loses its ability to enhance tubulin polymerization, as observed from the polymerization kinetics depicted in Fig. 2a. This suggests that the interaction with CB[7] negatively impacts the efficacy of Xyl-DTX in stabilizing microtubules. To ensure that CB[7] alone does not influence tubulin polymerization, we conducted control experiments comparing the polymerization kinetics of tubulin alone and tubulin in the presence of 200 μM CB[7]. The results, shown in ESI Fig. S10a,† indicated no significant difference in their polymerization kinetics, confirming that CB[7] alone does not have any impact on the polymerization process. Furthermore, we assessed whether the turbidity readout can be influenced by CB[7] solubility in the assay buffer. The kinetic readings, included in ESI Fig. S10b,† revealed no observable difference between the turbidity of the CB[7] solution (200 μM) and the buffer alone. This indicated that CB[7] does not have any solubility issues that could interfere with the measurements. Overall, the decrease in tubulin polymerization efficacy with the CB[7]·Xyl-DTX complex, as observed from the kinetic results, suggests that CB[7] threading reduces the binding affinity of Xyl-DTX to microtubules, leading to a loss of potency and bioactivity.

We further sought to gain molecular insights into the relative binding modes of Xyl-DTX and CB[7]·Xyl-DTX to tubulin *via* computational modeling and simulation approach. Towards this end, we employed a combination of *in silico* docking, molecular dynamics simulation technique, and free energy estimation approaches. For this purpose, we first employed *in silico* docking together with MD simulation to develop a stable pose of both the ligands (Xyl-DTX and CB[7]·Xyl-DTX) with the tubulin (see ESI Methods† for details). Subsequently, we simulated the profile of free-energy of binding of both Xyl-DTX and CB[7]·Xyl-DTX to tubulin *via* the metadynamics simulation approach.^53,54^ In this method, a bias potential is added at a predefined frequency along one or multiple collective variables that drive the system to rigorously explore both favorable and unfavorable states. Here, we have used the center of mass (COM) distance between the ligand molecule and the binding pocket as the collective variable to explore the unbinding event in order to determine the ligand binding free energy. For both Xyl-DTX and its complex with CB[7], three metadynamics runs were performed, and an average free energy profile was calculated, as presented in Fig. 2b, along with snapshots of the bound conformation, ascertained from the lowest free energy minimum. The binding free energy of Xyl-DTX to tubulin is ∼77 kJ mol^−1^, corresponding to a ligand-pocket COM distance of ∼0.6 nm. On the other hand, the binding free energy of the CB[7]·Xyl-DTX complex is relatively weaker, with a value of ∼66 kJ mol^−1^. More significantly, in the stable bound conformation of CB[7]·Xyl-DTX complex, the ligand is found to be further away from the tubulin-binding pocket with a COM distance of ∼1.3–1.5 nm, compared to that obtained in the absence of CB[7] (in which case the stable pose had a pocket-ligand distance of 0.6 nm). This suggests that the host–guest complexation of Xyl-DTX by CB[7] would render a less tight binding of the ligand to the tubulin than the uncomplexed ligand. The protein–ligand interaction profile for a representative bound conformation of Xyl-DTX and CB[7]·Xyl-DTX to tubulin is presented in Fig. 2c; the details of the interactions are tabulated in ESI Tables S2 and S3.† Analyzing the interactions indicates that the binding of Xyl-DTX to tubulin is stabilized by a network of electrostatic interactions such as hydrogen bonds and salt bridges as well as hydrophobic interactions. The relatively weaker binding of CB[7]·Xyl-DTX complex is found to lack electrostatic stabilization and can be mainly attributed to hydrophobic interactions. The presence of the CB[7] cage around the ligand would sterically hinder the tight binding of Xyl-DTX and would preclude the formation of favorable electrostatic interactions. These molecular insights corroborate with the experimental assays and suggest that when complexed with CB[7], there is a significant decrease in the binding affinity of Xyl-DTX to tubulin.

**Fig. 3:**
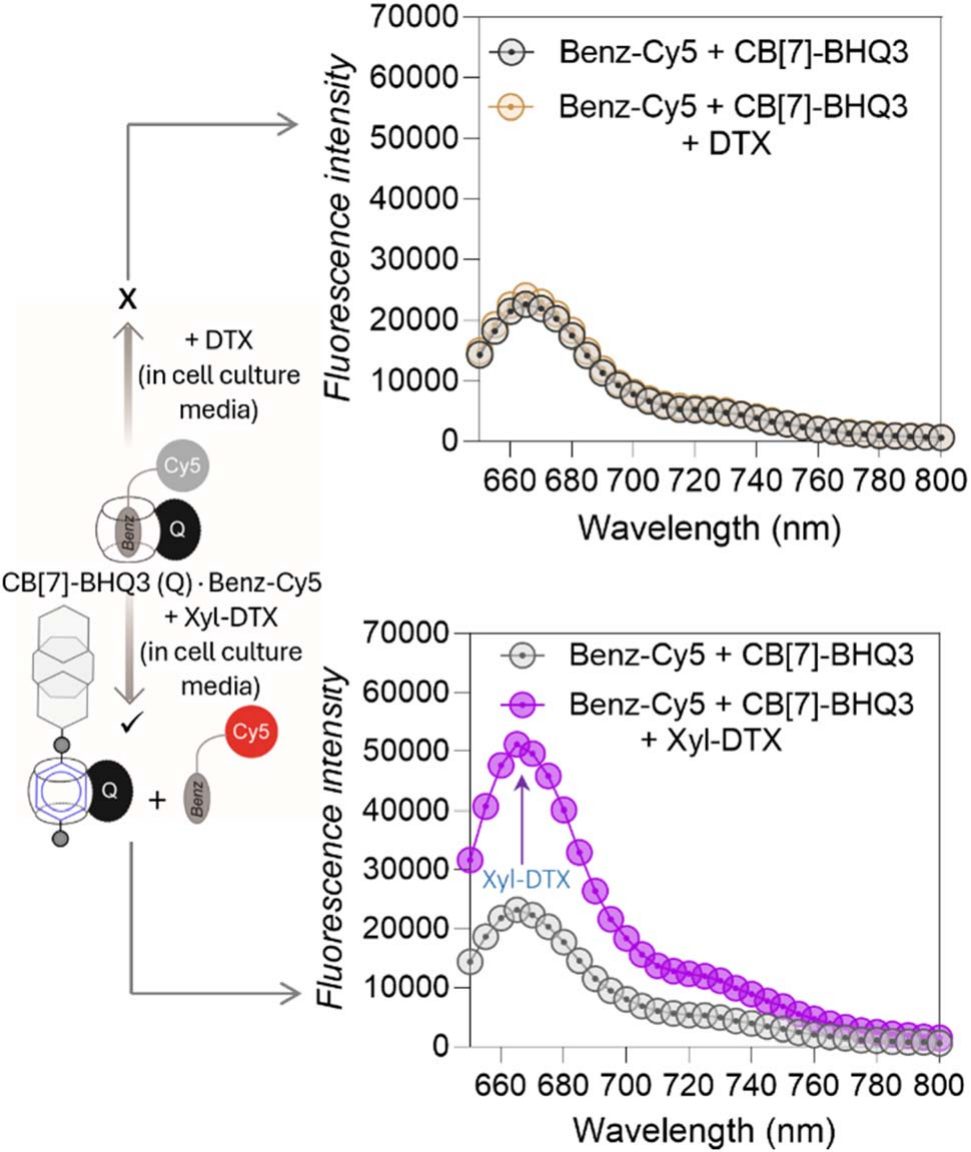
Displacement assay to study complexation of CB[7] and Xyl-DTX in cell culture media. The addition of Xyl-DTX (10 μM) to a quenched solution of CB[7]-BHQ3·Benz-Cy5 complex (prepared by mixing 1 μM of CB[7]-BHQ3 and 1 μM of Benz-Cy5) resulted in fluorescence recovery, indicating that Xyl-DTX displaced Benz-Cy5 and formed a CB[7]-BHQ3⋅Xyl-DTX complex. No fluorescence recovery was observed upon the addition of DTX (10 μM).

Before evaluating the MT-targeted activity of the host–guest complex in cells, we investigated whether the complexation between CB[7] and DTX-Xyl can occur in cell culture media. As evidenced by earlier studies, the media in which host and guest molecules are dispersed significantly influences their complex formation.^55,56^ Hence, we examined the formation of the CB[7]·Xyl complex in cell culture media, a crucial step preceding cellular studies aimed at understanding the impact of CB[7] complexation on cellular processes. First, to assess whether Xyl represents a suitable guest molecule that can form host–guest complexes with CB[7] in cell culture media, we utilized a Förster Resonance Energy Transfer (FRET)-based quenching assay employing a CB[7]-conjugated fluorophore (CB[7]-TAMRA) and a Xyl-conjugated quencher (Xyl-BHQ2) (ESI Fig. S11a†). Initially, we prepared a solution of CB[7]-TAMRA in cell culture media, recorded emission spectra, and then introduced Xyl-BHQ2. The decrease in TAMRA fluorescence indicated FRET-based quenching due to the close proximity of the fluorophore and quencher, suggesting the formation of a CB[7]-TAMRA·Xyl-BHQ2 host–guest complex in cell culture media. Control experiments with a quencher (EtA-BHQ2, see ESI Fig. S11b†) that lacks a binding partner for CB[7] did not lead to a decrease in fluorescence emission. This result highlights the essential role of the Xyl guest in forming a complex with CB[7]. Subsequently, to evaluate the complexation ability of the target compound Xyl-DTX with CB[7] in cell culture media, we performed displacement assay using a quenched FRET pair comprised of a benzyl guest conjugated Cy5 dye (Benz-Cy5) and a CB[7]-conjugated BHQ3 quencher (CB[7]-BHQ3(Q)) (Fig. 3 and S12†). The benzyl guest, with lower affinity (*K*_a_ ∼ 10^6^ M^−1^) for CB[7] compared to Xyl-DTX,^43^ can be displaced by Xyl-DTX, allowing fluorescence recovery as an indicator of Xyl-DTX complexation with CB[7]. In this assay, we first mixed Benz-Cy5 and CB[7]-BHQ3 in cell culture media in a microwell plate to form a quenched complex. Upon adding Xyl-DTX, fluorescence recovery was observed, indicating a separation between the fluorophore and quencher. This suggests that Xyl-DTX displaced Benz-Cy5 from the initial CB[7]-BHQ3·Benz-Cy5 complex and formed a new complex with CB[7] (CB[7]-BHQ3·Xyl-DTX complex) in the cell culture media (Fig. 3). A control well, where DTX was added, showed no change in fluorescence, confirming that the displacement was specific to Xyl-DTX and driven by the CB[7]·Xyl host–guest complexation. Further confirmation on the formation of CB[7]·Xyl-DTX was provided by MALDI-MS analysis of a mixture of CB[7] and Xyl-DTX prepared in cell culture media. The MALDI-MS spectra revealed a significant mass peak, corresponding to the 1 : 1 host–guest complex of CB[7] and Xyl-DTX (ESI Fig. S13†), validating the effective complexation of CB[7] and Xyl-DTX in the cell culture media. We also conducted experiments to confirm the formation of a host–guest complex between CB[7] and the Xyl guest within a simulated intracellular cellular environment. We observed specific fluorescence staining of the microtubule structure in fixed cells where Xyl was immobilized onto microtubules using an antibody (Ab)-based labeling approach, and complexation was performed with CB[7]-TAMRA in cell culture media (ESI Fig. S14†). This indicated the formation of the CB[7]-TAMRA·Xyl-Ab host–guest complex onto the microtubule structure in a simulated intracellular setting. Additionally, intracellular complexation of Xyl-DTX with CB[7] was investigated through a displacement assay, where Xyl-DTX was used to displace microtubule-bound CB[7]-TAMRA·Benz-Ab complex (ESI Fig. S15†). We found that incubation with Xyl-DTX significantly reduced microtubule fluorescence arising from CB[7]-TAMRA·Benz-Ab complexation, indicating successful displacement of dye stain (CB[7]-TAMRA) from microtubule structure and formation of a more stable CB[7]-TAMRA·Xyl-DTX complex. Control experiments with DTX alone showed no change in fluorescence, confirming that the high affinity of Xyl-DTX for CB[7] is essential for the displacement. Overall, these observations support the formation of the CB[7]·Xyl-DTX complex within a simulated intracellular setting.

**Fig. 4:**
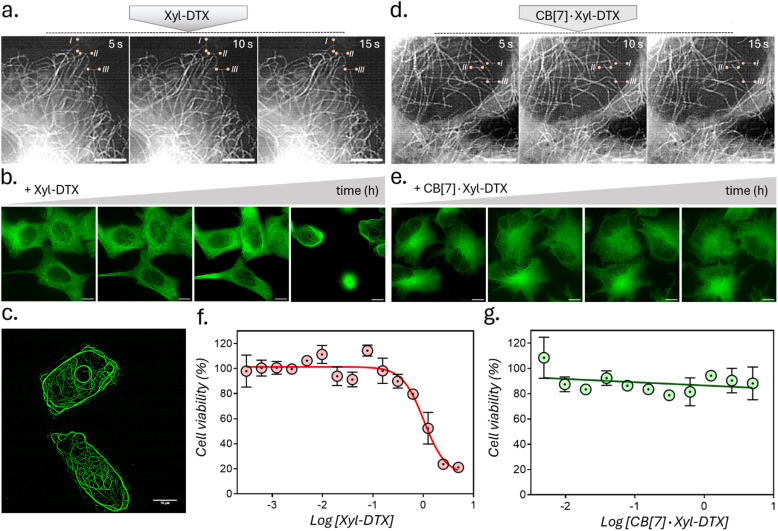
Effect of CB[7] complexation with Xyl-DTX on the structure, dynamics, and function of MTs in HeLa cells. (a and d) Time-lapse SIM fluorescence images at 5s intervals after treatment with (a) Xyl-DTX and (d) CB[7]·Xyl-DTX to live GFP-α-tubulin expressing HeLa cells. These images were used to evaluate MT dynamics. Reference lines were drawn to better understand the changes in the positions of the MT ends. Images in (a) were recorded after 15 h of incubation with Xyl-DTX, and images in (d) were recorded after 17 h of incubation with CB[7]·Xyl-DTX. (b and e) SIM fluorescence images over a period of several hours, illustrating the eventual bundling of MTs in the case of treatment with Xyl-DTX (5 μM). However, incubating cells with CB[7]·Xyl-DTX complex, formed by mixing 100 μM CB[7] with 5 μM Xyl-DTX, did not induce any significant changes in MT structure over time. Time points in (b) include 0 h, 3 h, 6 h, and 17 h, while time points in (e) include 10.5 h, 12.5 h, 20.5 h, and 22.5 h. (c) Zoomed-in SIM fluorescence image of HeLa cells, showing extensive MT bundling, after treating with Xyl-DTX (5 μM) after 17 h of incubation. (f and g) Dose–response curve for (f) Xyl-DTX and (g) CB[7]·Xyl-DTX in HeLa cells. Scale bar: 5 μm (a and d) and 10 μm (b, c and e).

We next assessed the effect of CB[7] complexation with Xyl-DTX on the structure, dynamics, and function of MTs in live cells. To directly visualize the impact on cellular MTs, we carried out time-lapse super-resolved Structured Illumination Microscopy (SIM) imaging of HeLa cells expressing GFP labelled α-tubulin.^57,58^ We first examined the effect of Xyl-DTX (3 μM) on MT dynamics (Fig. 4a). Time-lapse SIM fluorescence images captured at 5s intervals showed minimal changes in the positions of MT ends, indicating significantly suppressed microtubule dynamics. Subsequently, time-lapse super-resolved SIM fluorescence images were acquired in the GFP channel for over 24 h to observe changes in MT structure. As shown in Fig. 4b, c, and S16,† we noticed a drastic change in typical filamentous MT structure with the appearance of MT bundling. The extent of MT bundling gradually increased over time and ultimately led to cell death. This bundling of MTs is a classical hallmark of MT stabilizing agents, especially at higher concentrations.^59,60^ Generally, MTs are formed preferentially upon the action of the stabilizing agents, and they usually show reduced dynamicity.^61,62^ Moreover, these stabilized and less dynamic MTs have been known to associate with each other *via* close wall-to-wall alignments, or they get complexed with some C-shaped protofilament ribbons, ultimately resulting in the formation of MT bundles. The occurrence of bundling eventually affects the vital function of MTs in the process of cell division, leading to cell death. Therefore, the observation of suppressed dynamicity and MT bundling from our time-lapse fluorescence imaging experiment validates the MT stabilizing behavior of Xyl-DTX and clearly indicates a specific MT-targeted mode of action for Xyl-DTX conjugate in the live cell. Next, we evaluated the effect of CB[7] complexation with Xyl-DTX on the structure and function of MTs. Accordingly, Xyl-DTX in the form of a CB[7]·Xyl-DTX complex was incubated with the cells. Internalization was confirmed by both MALDI-MS analysis and LC-MS (liquid chromatography-mass spectrometry) (see ESI Fig. S17 and S18†). Subsequently, time-lapse fluorescence imaging was performed. Interestingly, contrary to Xyl-DTX alone, CB[7]·Xyl-DTX complex had minimal effect on the MT dynamics and structures. As shown in Fig. 4d, images acquired at 5s intervals, after treatment with a mixture of Xyl-DTX (3 μM) and CB[7] (60 μM) showed significant changes in the positions of MT ends. These changes in length over time of individual MT clearly illustrate the dynamic behavior of the MTs. Additionally, MT structure largely remained unperturbed over a ∼22 h period, and no appreciable MT bundling was observed from the cells (Fig. 4e and S19†). These observations clearly indicate that the complexation with CB[7] significantly reduced the MT stabilizing potency of Xyl-DTX in the live cell, which was also supported by our *in vitro* studies and simulation results. Subsequently, the efficacy of the Xyl-DTX conjugate in inducing mitotic arrest, resulting in cell death, was quantified by the MTT assay. HeLa cells were treated with increasing concentrations of the Xyl-DTX derivative in the presence of the efflux pump inhibitor verapamil for a period of 24 h. After washing, cells were further maintained at 37 °C for 24 h before performing the MTT assay. As shown in Fig. 4f, the percentage of cell survival decreased gradually with the increase in the concentration of Xyl-DTX. IC50 (half-maximum inhibitory concentration) was determined to be ∼1 μM from the fitted dose–response curve. We next performed the MTT assay to assess the effect of the CB[7]·Xyl-DTX in cell survival. The dose–response curve, shown in Fig. 4g, depicts that CB[7] complexation results in a significantly decreased toxicity profile of Xyl-DTX. Interestingly, no noticeable change in cell viability was observed up to a concentration range (5 μM) where Xyl-DTX alone showed a ∼80% decrease in cell viability, indicating a strong influence of CB[7] inclusion complex formation on the toxicity profile of Xyl-DTX. Further, to establish that derivatization with Xyl played a key role in achieving CB[7] mediated cellular effect from DTX, we performed a toxicity assay with native DTX in the presence and absence of CB[7]. As shown in ESI Fig. S20,† we did not observe any change in DTX activity in the presence of CB[7], indicating the importance of derivatization with high-affinity CB[7] recognition moiety for modulating DTX activity.

**Fig. 5:**
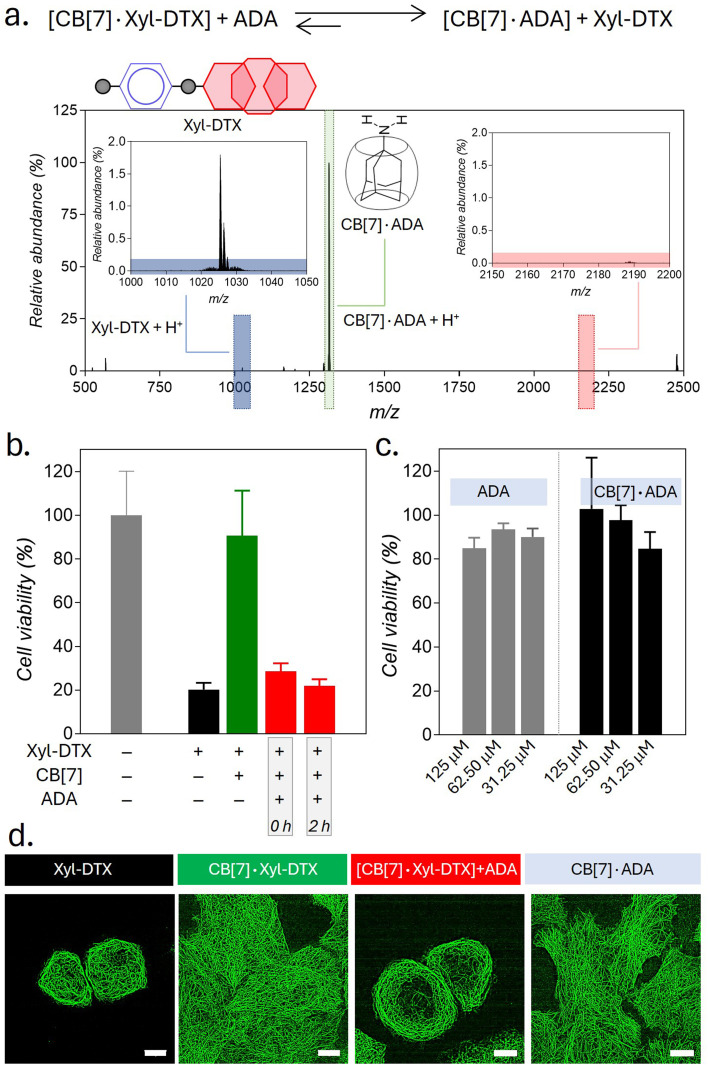
Triggering MT targeted activity using ADA chemical signal. (a) Positive MALDI-MS spectrum of ADA added CB[7]·DTX-Xyl complex solution in water, showing displacement of Xyl-DTX by ADA from the CB[7] cavity. Calculated *m*/*z* for Xyl-DTX + H^+^: 1025.51, found: 1025.43; calculated *m*/*z* for CB[7]·ADA + H^+^: 1314.49, found: 1314.47. (b) Cell viability in the presence of CB[7]·Xyl-DTX complex after *in situ* addition of ADA at different time points. Xyl-DTX: 2.5 μM, CB[7]: 50 μM, ADA: 50 μM (c) Cell viability in the presence of ADA and CB[7]·ADA complex. (d) SIM images of GFP-α-tubulin expressing HeLa cells showing the effect of ADA activation on MT structure. Scale bar: 10 μm (d).

Supramolecular host–guest complexes, as in the case of CB[7]·Xyl-DTX, make use of non-covalent interactions to achieve the controlled assembly of the molecular components. In addition to the modification of molecular properties, non-covalent supramolecular complexation provides the facility for actuation through competitive interaction with orthogonally presented guest molecules, enabling complex and signal-responsive behavior within living systems. To achieve competitive displacement of the Xyl guest from CB[7] cavity, we explored the use of an orthogonal guest molecule, which possesses a higher affinity towards CB[7] than Xyl. We tested whether the host–guest complex between CB[7]·Xyl-DTX can be disassembled by the presentation of orthogonal guest molecule 1-adamantylamine (ADA), which is a relatively high-affinity (*K*_a_ of ∼10^12–13^ M^−1^) binding competitor for CB[7] as compared to Xyl-DTX. We first investigated competitive unsheathing of the CB[7]·Xyl-DTX host–guest complexes by ADA using MALDI-MS analysis. We acquired MALDI-MS spectra after the addition of a 10 μM solution of ADA for 15 min to a CB[7]·Xyl-DTX complex solution (prepared by mixing 10 μM CB[7] with 10 μM Xyl-DTX) in water. The MALDI-MS spectra of this mixture displayed a mass signature at 1314.47 *m*/*z*, corresponding to the high-affinity CB[7]·ADA complex, along with a mass signature at *m*/*z* of 1025.43 for the released Xyl-DTX (Fig. 5a), indicating the transformation of CB[7]·Xyl-DTX to Xyl-DTX*via* ADA mediated dethreading. We further investigated the displacement of a Xyl guest by the high-affinity ADA guest in both cell culture media and cellular environments. Using the FRET pair (CB[7]-TAMRA and Xyl-BHQ2), we found that ADA addition to a quenched CB[7]-TAMRA·Xyl-BHQ2 complex in cell culture media leads to an increase in TAMRA fluorescence (ESI Fig. S21a†). In contrast, the addition of a low-affinity cyclohexyl guest did not result in any change in fluorescence from the quenched complex (ESI Fig. S21b†). This observation suggests that the high-affinity guest ADA can separate the fluorophore and quencher by displacing Xyl from the CB[7] cavity, forming a CB[7]·ADA complex in cell culture media. In a cellular setting, adding ADA to fixed cells with microtubules labeled *via* the CB[7]-FL·Xyl-Ab complex resulted in a loss of fluorescence signal from microtubule structures (ESI Fig. S22†). This suggests that CB[7]-FLs are no longer attached to Xyl-Ab, having been displaced by ADA through the formation of a CB[7]-FL·ADA complex. Further, mass signatures from MALDI-MS spectrometry confirmed that ADA could displace a CB[7]·Xyl-DTX complex in cell culture media, forming a new CB[7]·ADA complex (ESI Fig. S23†). These findings suggest that ADA can effectively release Xyl-DTX from the CB[7] cavity in both cell culture media and cellular environments, forming stable CB[7]·ADA complexes. Notably, the observed dethreading of the supramolecular complex in the cellular context raises the possibility that MT-targeted activity from CB[7]·Xyl-DTX complex can now be triggered by the use of ADA chemical signal. To test this hypothesis, HeLa cells were incubated with CB[7]·Xyl-DTX complex, and subsequently, ADA trigger was added at 0 h and 2 h time points. As shown in Fig. 5b, incubation of the Xyl-DTX, complexed with CB[7], results in increased cell survival (∼90% cell viability) as compared to the Xyl-DTX alone (∼20% cell viability). Interestingly, adding ADA to CB[7]·Xyl-DTX treated cells led to a significant decrease in cell viability to ∼22%, approaching the level of cell viability observed from the Xyl-DTX alone. Overall, these results indicate that ADA acts as an effective trigger for the disassembly of CB[7]·Xyl-DTX, restoring the specific MT-targeted biological activity of Xyl-DTX. Additionally, control experiments where cells were just treated with the ADA trigger or its complex with CB[7] ensured that the observed cytotoxicity was primarily due to the disassembly of the CB[7]·Xyl-DTX complex (Fig. 5c). To support the restoration of MT targeted activity upon ADA trigger, we also carried out fluorescence imaging of HeLa cells expressing GFP labelled α-tubulin. As seen in Fig. 5d and S24,† cells treated with the Xyl-DTX conjugate alone resulted in MT bundling. However, upon treatment of cells with the CB[7]·Xyl-DTX complex, MT structures were observed to be intact. Upon addition of ADA to CB[7]·Xyl-DTX treated cells, substantial MT bundling was observed, indicating restoration of specific MT targeting activity by ADA trigger. Overall, our experimental findings indicate that ADA is capable of displacing Xyl-DTX from the CB[7]·Xyl-DTX complex in the cellular environment by forming a CB[7]·ADA complex, leading to the restoration of MT-targeted effects *via* ADA chemical signal.

**Fig. 6:**
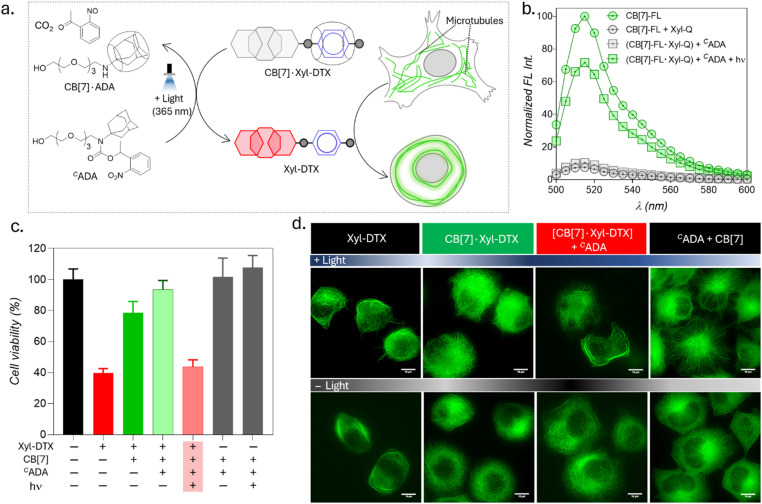
Light-induced modulation of MT function through photochemical activation of guest molecules. (a) Schematic representation illustrating the activation of the CB[7]·Xyl-DTX complex using ^C^ADA under light irradiation and the subsequent effects on MT. (b) Fluorescence displacement assay in PBS (pH = 7.4) demonstrating that ^C^ADA effectively displaces Xyl from the CB[7] cavity upon light stimulation. CB[7]-FL: 1 μM, Xyl-Q: 1 μM, ^C^ADA: 5 μM. (c) MTT assay results for HeLa cells treated with the CB[7]·Xyl-DTX complex in the presence of ^C^ADA, with and without light irradiation. Xyl-DTX: 3 μM, CB[7]: 60 μM, ^C^ADA: 60 μM. (d) SIM images of GFP-α-tubulin-expressing HeLa cells showing MT bundling from the effect of light-mediated activation of the CB[7]·Xyl-DTX complex by ^C^ADA. Scale bar: 10 μm (d).

Upon achieving regulation of MT structure and function *via* ADA-mediated competitive guest displacement reaction, we aimed to investigate whether we can gain optical control over the MT function through an interconnected network that provides ADA guest activation under photochemically controlled conditions (Fig. 6a). Optical stimulus is an important external trigger that can provide both spatial and temporal control over the function of the biomolecules under non-invasive conditions. Toward this aim, we synthesized a caged ADA derivative (^C^ADA) that lacks CB[7] affinity due to the presence of an *o*-nitrobenzyl group attached to its amine functionality. The presence of the o-nitro benzyl group sterically blocks ADA encapsulation inside the CB[7] cavity as well as inhibits electrostatic interaction with its carbonyl portal, resulting in a diminished affinity of ADA moiety towards CB[7].^30^ However, the *o*-nitrobenzyl group from the ^C^ADA can be efficiently cleaved to generate the high-affinity ADA guest under 365 nm light illumination. Photouncaging of the ^C^ADA can then lead to the disassembly of CB[7]·Xyl-DTX complex to release the active Xyl-DTX by forming CB[7]·ADA complex, thereby placing the MT targeted activity under optical control. We initiated our investigation with a fluorescence displacement assay, utilizing CB[7]-BODIPY (CB[7]-FL) fluorophore and Xyl-BHQ1 (Xyl-Q) quencher pair, to assess the impact of ^C^ADA. This method enabled us to verify that ^C^ADA effectively displaces Xyl from the CB[7] cavity upon light stimulation, as indicated by the restoration of fluorescence emission following 365 nm light irradiation (Fig. 6b). Next, we conducted an MTT cell viability assay on the CB[7]·Xyl-DTX complex in the presence of photocaged ADA (^C^ADA), both with and without light irradiation. We observed a significant decrease in cell viability when ^C^ADA was cleaved by light. However, in the absence of light, there was no significant change in cell viability. Control experiments involving only light irradiation on cells or the presence of CB[7] and ^C^ADA (with and without light) showed no significant toxicity from the light or any reactants/reaction products (Fig. 6c and S25†). Subsequently, in order to visualize the optical effect on MT structure, CB[7]·Xyl-DTX treated HeLa cells expressing GFP labeled α-tubulin cells were incubated with ^C^ADA for 2 h. Afterward, a set of cells was irradiated with 365 nm light from an LED source for 100 s. In a controlled study, another group of cells continued to be maintained under dark conditions for a period of ∼24 h after incubation with CB[7]·Xyl-DTX and ^C^ADA. In order to understand the effect on MTs, we acquired fluorescence images from the HeLa cells expressing GFP labeled α-tubulin. As shown in Fig. 6d and S26,† HeLa cells treated with the CB[7]·Xyl-DTX complex followed by ^C^ADA incubation and light irradiation led to a significantly enhanced MT bundling as compared to the control set of cells that were maintained under dark conditions. This indicates that photouncaging of ^C^ADA leads to the disassembly of CB[7]·Xyl-DTX complex, leading to the restoration of Xyl-DTX bioactivity. Notably, other control studies, where cells were irradiated with light after incubation with only CB[7]·Xyl-DTX or with a mixture of ^C^ADA and CB[7] did not display any significant alteration of microtubule morphology, indicating the minimal influence of light irradiation on the observed MT alteration. Overall, the observed MT targeted activity by photouncaging of ^C^ADA in the presence of CB[7]·Xyl-DTX complex establishes an optical control over the MT function through guest activation under photochemically controlled conditions.

**Fig. 7:**
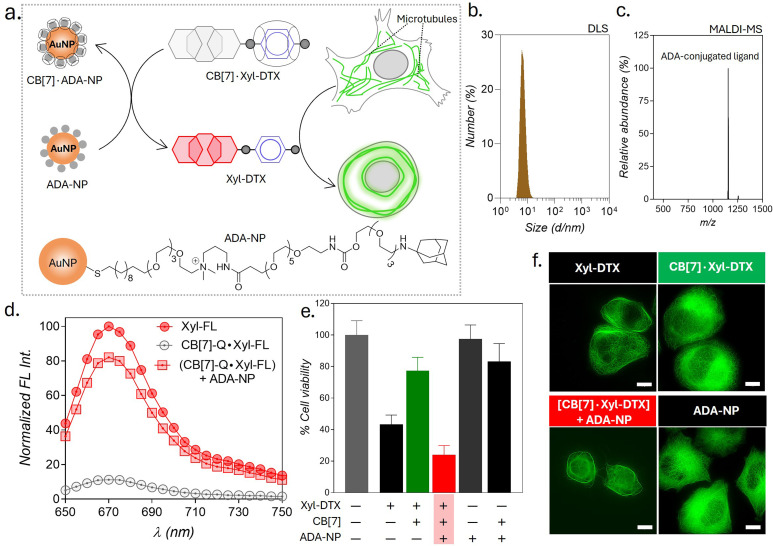
Triggering the activity of CB[7]·Xyl-DTX complex by using ADA functionalized gold nanoparticles (ADA-NPs). (a) Schematic showing activation strategy of Xyl-DTX using ADA-NP and its subsequent effect on MT. (b) Dynamic Light Scattering (DLS) analysis of ADA-NPs. (c) Positive MALDI-MS spectrum of ADA-NP showing the mass signature corresponding to the ADA-conjugated ligand. Calculated *m*/*z* for ADA-conjugated ligand: 1153.79, found: 1153.95. (d) *In vitro* fluorescence displacement studies in PBS (pH = 7.4) depicting the recovery of fluorescence of Xyl-SiR with the addition of ADA-NP. Xyl-FL: 1 μM, CB[7]-Q: 1.5 μM, ADA-NP: 20 nM (e) MTT assay results for HeLa cells treated with CB[7]·Xyl-DTX complex in the presence of ADA-NP. Xyl-DTX: 3 μM, CB[7]: 18 μM, ADA-NP: 125 nM. (f) Cellular imaging of GFP-α-tubulin labeled cells showing the effect of ADA-NP activation on MT structure. Scale bar: 10 μm (f).

Therapeutic strategies based on the use of nanoparticles provide promising methodologies for improving disease treatment with reduced side effects. Engineered nanoparticles with an appropriate size and surface chemistry can preferentially accumulate in tumor tissue either due to enhanced permeability and retention (EPR) effect or through an active targeting mechanism.^63^ Taking advantage of their preferential accumulation in tumor tissue, we thought to investigate whether a nanoparticle can be used to activate the MT-targeted therapeutic effect from the CB[7]·Xyl-DTX complex *via* exploiting nanoparticle-mediated guest displacement reaction (Fig. 7a). Accordingly, we have synthesized a biocompatible nanoparticle with a gold core that is surface-functionalized with high-affinity ADA guest *via* a polyethylene glycol linker. Detailed protocol for the synthesis of the nanoparticle has been given in ESI Scheme 1.† In brief, to synthesize this ADA conjugated gold nanoparticle (ADA-NP), we used an amine-functionalized gold nanoparticle (AuNMe_2_-Prp-NH_2_), which was first reacted with an NHS ester derivative of ^C^ADA.^64^ The ^C^ADA functionalized nanoparticles were then irradiated with UV light to remove the photo-protecting group and to obtain the ADA-decorated NPs (ADA-NPs). ADA-NPs were characterized using Transmission Electron Microscopy (TEM) and Dynamic Light Scattering (DLS). TEM images revealed detailed morphology with an average core size of *d* = 2.03 ± 0.55 nm (ESI Fig. S27†). DLS measurements indicated a hydrodynamic diameter of 6.84 ± 2.71 nm, accounting for both the core and surface-grafted ligands, reflecting the nanoparticle's size in a hydrated state (Fig. 7b). The surface attachment of ADA molecules was verified through a fluorescence titration assay using CB[7]-TAMRA (CB[7]-FL). In this experiment, the addition of ADA-NPs to a solution of CB[7]-FL resulted in a significant fluorescence quenching (ESI Fig. S28†). This quenching effect arises due to the close proximity of the fluorophore (CB[7]-FL) to the gold core of the nanoparticle,^65^ which occurs when CB[7]-FL binds to ADA molecules on the nanoparticle's surface. This observation supports the successful anchoring of ADA molecules to the nanoparticle surface. Furthermore, this fluorescence titration allowed us to estimate the number of ADA moieties per nanoparticle, which was ∼60/nanoparticle. MALDI-MS analysis further corroborated the presence of ADA-conjugated ligands, showing a peak at *m*/*z* of 1153.95, corresponding to the molecular ion peak of the ADA-appended surface ligands (Fig. 7c). We then titrated a quenched pair of Xyl-SiR fluorophore and CB[7]-BHQ3 quencher with ADA-NPs and observed fluorescence recovery after the addition of ADA-NPs (Fig. 7d), indicating the nanoparticle's ability to enable displacement of xylene conjugated SiR fluorophore from CB[7] cavity *via* competitive host–guest interactions. To understand the effect of ADA-NPs trigger on cell survival, we incubated CB[7]·Xyl-DTX complex treated cells with ADA-NPs and evaluated the cytotoxicity *via* MTT assay. As shown in Fig. 7e, while incubation of CB[7]·Xyl-DTX complex alone did not show any significant toxicity, the addition of ADA-NPs to the CB[7]·Xyl-DTX treated cells showed a significant reduction in cell viability. This indicates that the addition of ADA-NPs triggered the release of the Xyl-DTX from the supramolecular CB[7]·Xyl-DTX complex and restored the cytotoxicity of Xyl-DTX. In addition, control experiments performed with ADA-NPs alone or its complex with CB[7] (CB[7]·ADA-NP) did not display any significant cytotoxicity to the cell, thereby providing assurance that the cellular effect is mainly due to the restoration of bioactivity of Xyl-DTX upon displacement by the ADA-NPs. We have further verified the MT-targeted effect from these systems *via* cellular imaging of HeLa cells expressing GFP labeled α-tubulin. As shown in Fig. 7f and S29,† HeLa cells treated with the CB[7]·Xyl-DTX complex followed by treatment with ADA-NPs displayed cellular death with evident MT bundling, while the control experiments with ADA-NPs did not show any toxic effects and change in MT morphology. Overall, these results establish that CB[7]·Xyl-DTX host–guest complex can be activated by a nanoparticle-based trigger signal, potentially achieving a localized effect for a better therapeutic outcome.

## Conclusion

In this study, we have described a supramolecular approach based on CB[7] mediated host–guest recognition to regulate the dynamics, structure, and functions of MTs under chemical, light, and nanoparticle signals. A two-faced Xyl-DTX derivative, containing an MT binding epitope and a CB[7] recognition moiety, was used to establish a communication between MTs and CB[7] host–guest chemistry. *In situ* probing using GFP-α-tubulin expressing cells indicated an effective MT stabilization and bundling by Xyl-DTX, whereas threading of CB[7] with Xyl-DTX significantly reduced its effect on MTs. Simulation studies were further used to get molecular insights into the differential activities of Xyl-DTX and CB[7]·Xyl-DTX towards MT. Importantly, we could achieve reversible activation of MT stabilizing effect by dethreading CB[7]·Xyl-DTX complex using a high-affinity ADA guest. Additionally, by employing a photochemically activable ^C^ADA guest, we were able to gain control over the MT function by an optical signal, which holds tremendous potential for spatiotemporally regulated biological studies. Finally, we demonstrated the therapeutic potential of this supramolecular approach, where the therapeutic effect of CB[7]·Xyl-DTX was activated by a nanoparticle trigger. Overall, our studies highlight that by exploiting user-defined molecular recognition properties, supramolecular systems can achieve external/user-defined control over the underlying biomolecular network in the living system, paving the way for a diverse range of fundamental studies and future biotechnological applications.

## Data availability

All the necessary data has been provided in the ESI.†

## Author contributions

A. S., M. S. B., and S. S. A. conceived the study, designed and performed the experiments, analyzed and interpreted the data, and wrote the manuscript. S. M. and J. M. conducted the simulation studies and contributed to writing the manuscript. P. K. assisted with the cell culture studies. T. M. and S. R. carried out the binding affinity experiments and also contributed to writing the manuscript. S. K. assisted with the experiments involving GFP-α-tubulin expressing cells and offered critical insights for the manuscript. S. S. A. supervised the overall study.

## Conflicts of interest

There are no conflicts to declare.

## Supplementary Material

SC-015-D4SC00204K-s001
