# Supplementary material for: Regulation of microtubule dynamics and function in living cells via cucurbit[7]uril host–guest assembly
Source: Chem Sci. 2024 Jun 20;15(30):11981–94. doi: 10.1039/d4sc00204k (PMC11290447; doi:10.1039/d4sc00204k)
Supplement: SC-015-D4SC00204K-s001 [file SC-015-D4SC00204K-s001.pdf]

Electronic Supplementary Material (ESI) for Chemical Science.
This journal is © The Royal Society of Chemistry 2024

Supporting information

for

Regulation of microtubule dynamics and function in living cells *via* cucurbit[7]uril host-guest assembly

Akshay Saroha,[a],‡ Monica Swetha Bosco,[a],‡ Sneha Menon,[b] Pratibha Kumari,[a] Tanmoy Maity,[c] Subinoy Rana,[c] Sachin Kotak,[d] Jagannath Mondal,[b] and Sarit S. Agasti*[a],[e],[f]

[a]New Chemistry Unit, Jawaharlal Nehru Centre for Advanced Scientific Research (JNCASR), Bangalore, Karnataka 560064, India.

[b]Tata Institute of Fundamental Research, 36/P, Gopanpally Village, Hyderabad, 500046, India

[c]Materials Research Centre, Indian Institute of Science, C. V. Raman Road, Bangalore, 560012, India

[d]Department of Microbiology and Cell Biology, Indian Institute of Science, 560012, Bangalore, India

[e]Chemistry & Physics of Materials Unit, Jawaharlal Nehru Centre for Advanced Scientific Research (JNCASR), Bangalore, Karnataka, 560064, India.

[f]School of Advanced Materials (SAMat), Jawaharlal Nehru Centre for Advanced Scientific Research (JNCASR), Bangalore, Karnataka, 560064, India

‡These authors contributed equally to this work.

E-mail: *sagasti@jncasr.ac.in*

SUPPORTING INFORMATION

Table of contents

Contents	Page Number
1. General: materials and methods	3
2. Cell lines and their growth conditions	4
3. Synthesis protocol for preparation of docetaxel conjugated xylene (Xyl-DTX)	5
4. MALDI-MS characterization of host-guest complexes in water	7
5. Isothermal titration calorimetry (ITC)	8
6. Fluorescence titration with CB[7]·berberine complex	9
7. Turbidometry assay	10
8. Computational methods	12
9. Fluorescence assay for detection of Xyl binding to CB[7] in phenol-red free DMEM media (containing 10% FBS).	15
10. Fluorescence displacement assay for determination of binding of Xyl-DTX with CB[7] in Phenol-red free DMEM media (containing 10% FBS)	16
11. MALDI-MS based analysis of CB[7]·Xyl-DTX complexation in phenol-red free DMEM media (containing 10% FBS)	17
12. Cell-imaging experiment for detection of Xyl binding to CB[7] in phenol-red free DMEM media (containing 10% FBS).	17
13. Cell imaging experiment for detection of Xyl-DTX binding to CB[7] in phenol-red free DMEM media (containing 10% FBS).	19
14. Cellular assays and cell imaging with Xyl-DTX and CB[7]·Xyl-DTX	20
15. Fluorescence-based displacement study of Xyl guest by high-affinity ADA in phenol-red free DMEM media (containing 10% FBS)	26
16. Displacement of Xyl guest by high-affinity ADA in the cellular environment	27
17. MALDI-MS characterization of ADA triggered displacement in phenol-red free DMEM media (containing 10% FBS)	28
18. Cellular assays and cell imaging studies upon activation of CB[7]·Xyl-DTX complex with ADA	29
19. Experiments with ^{C}ADA	31
20. Synthesis and experiments with ADA-NP	35

21. Concentration measurement of Xyl-DTX using UV-Visible study.	42
22. Microscopy setup and image processing	42
23. Synthesis of Benz-Cy5	43
24. Preparation of Benz-conjugated antibody	43
25. Figures	47
26. References	85

1. General: materials and methods

All chemicals were purchased from Sigma Aldrich (Merck), Alfa Aesar, Fisher Scientific, TCI Chemicals, SD Fine Chemicals, or Spectrochem, unless otherwise specified. When necessary, solvents were dried using standard methods before use in reactions. High-performance liquid chromatography (HPLC) purification was performed using an Agilent 1260 Infinity Quaternary HPLC system equipped with a ZORBAX Eclipse Plus C18 column (4.6 mm × 100 mm, 3.5 μm). The eluents used were solvent A (water containing 0.1% TFA) and solvent B (acetonitrile containing 0.1% TFA). The concentrations of fluorophores and quenchers were measured with an Eppendorf BioSpectrometer. Fluorescence measurements were taken using a Perkin Elmer LS-55 Luminescence Spectrometer and a Plate Reader Synergy H1 (BioTek). Fluorescence cell imaging was conducted with an inverted Zeiss ELYRA PS1 microscope. ^{1}H NMR spectra were recorded on a Bruker ADVANCE III 400 MHz instrument and a JEOL 600 MHz instrument, with data analyzed using TopSpin 3.5pl7, SpinWorks 4, and JEOL Delta v5.0.5.1. Matrix-assisted laser desorption/ionization mass spectrometry (MALDI-MS) was performed in the reflection mode on Bruker Daltonics (Autoflex speed time-of-flight mass spectrometer) equipped with a Bruker smartbeam-II (Nd:YAG, 355 nm wavelength) laser. High-resolution mass spectrometry (HRMS) was performed on an Agilent 6538 Ultra High Definition (UHD) Accurate-Mass Q-TOF LC/MS. Liquid chromatography-mass spectrometry (LC-MS) was carried out using a Waters Alliance HPLC instrument with an SQ Detector 2 mass analyzer. LC-MS study for cellular uptake of the host-guest complex of the drug conjugate was carried out in (Dionex Ultimate) 3000 UHPLC (Thermo Fisher Scientific) system connected with MS (Q Exactive HF from Thermo Fisher Scientific). Two types of light sources were used for light-triggered uncaging studies: (1) THORLABS (CS2010-UV curing LED system, 365 nm) and (2) Biobee Tech Handheld UV lamps, 75 W, 365 nm (used in cytotoxicity study, given details in ^{C}ADA section).

Supporting Table S1: List of reagents, culture media, and antibodies used in cellular experiments and their sources.

Reagents, culture media, and antibodies	Commercial Source
Fetal bovine serum (FBS)	Gibco (10270-106)
Dulbecco's Modified Eagle Medium (DMEM) powder	Gibco (12800-017)
Dulbecco's Phosphate Buffered Saline	Gibco (14190-144)
Dulbecco's Modified Eagle Medium (DMEM) Phenol-red free media	Gibco (21063-029)
Verapamil Hydrochloride	TCI (Cat. No. 152-11-4)
3-(4,5-dimethylthiazol-2-yl)-2,5-diphenyltetrazolium bromide (MTT)	TCI (Cat. No. 298-93-1)
Docetaxel (DTX)	TCI (Cat. No. 114977-27-5)
Primary Antibody: Microtubule (α-tubulin), Species: Rat	Thermo Fisher Scientific (MAI-80017)
Secondary Antibody:<br>Target: Rat<br>Host: Donkey	Donkey Anti-Rat IgG (H+L)<br>(min X Bov, Ck, Gt, GP, SyHms, Hrs, Hu, Ms, Rb, Shp Sr Prot)<br>Jackson Immuno Research Laboratories<br>(Cat. No. 712-005-153)

2. Cell lines and their growth conditions

1. HeLa cells: HeLa cell line is kindly provided by Prof. Maneesha Inamdar lab at Jawaharlal Nehru Centre for Advanced Scientific Research (JNCASR), Bangalore, Karnataka 560064, India. HeLa cells were cultured in a humidified atmosphere (5% CO_2) at 37°C and grown in Dulbecco's Modified Eagle's Medium (DMEM, high glucose) supplemented with 10% fetal bovine serum (FBS) (Gibco, USA), 2 mM Glutamax (Invitrogen, USA) and 1% antibiotics (100 U/ml penicillin, 100 µg/ml streptomycin and 0.25 µg/ml amphotericin) (Gibco, USA).

2. *HeLa cells expressing GFP-labeled α-tubulin:* This cell line was generously provided by Daniel Gerlich from the Institute of Molecular Biotechnology, Vienna, Austria. This cell line has been previously used in the following literature: REF. 57: M. H. A. Schmitz, M. Held, V. Janssens, J. R. A. Hutchins, O. Hudecz, E. Ivanova, J. Goris, L. Trinkle-Mulcahy, A. I. Lamond, I. Poser, A. A. Hyman, K. Mechtler, J.-M. Peters, and D. W. Gerlich, *Nat. Cell Biol.*, 2010, **12**, 886–893. REF. 58: S. Kotak, C. Busso, and P. Gönczy, *EMBO J.*, 2014, **33**, 1815–1830. This HeLa Kyoto cell line expresses both EGFP-α-tubulin and mCherry-H2B. As our research focused solely on the tubulin structure and dynamics using GFP fluorescence, we referred to this cell line as HeLa cells expressing GFP-labeled α-tubulin. HeLa cells expressing GFP-labeled α-tubulin were cultured in high-glucose DMEM supplemented with 10% fetal bovine serum (10270-106; Gibco) at 37°C in a humidified 5% CO_2 incubator.

3. *BS-C-1 cells*: BS-C1 cell line is kindly provided by Nitin Mohan group at IIT-Kanpur (INDIA). BS-C-1 cell was cultured in a humidified atmosphere (5% CO_2) at 37°C and grown in Minimum Essential Medium (MEM) containing (+) non-essential amino acids (NEAA) and no glutamine, which was further supplemented with 10% fetal bovine serum (FBS) (Gibco, USA), 2 mM Glutamax (Invitrogen, USA) and 1% antibiotics (100 U/ml penicillin, 100 μg/ml streptomycin and 0.25 μg/ml amphotericin) (Gibco, USA), 1 mM Sodium Pyruvate (Gibco, USA).

3. Synthesis protocol for preparation of docetaxel conjugated Xyl (Xyl-DTX)

3.1 Deprotection of Docetaxel to prepare amine derivative of docetaxel (DTX-NH_2):

Docetaxel (5 mg) was taken in a 5 ml glass vial, and 0.5 ml formic acid was added to it. The reaction mixture was kept for stirring at room temperature (25°C) for 60 min. Subsequently, the reaction mixture was evaporated to dryness under a high vacuum and further lyophilized to get the amine derivative of docetaxel (DTX-NH_2). Yield: 95% DTX-NH_2 was characterized by HRMS and was subsequently used for the conjugation step. HRMS (ESI-MS) calculated for $C_{38}H_{46}NO_{12}^+$: 708.3015, found: 708.2991 [M+H$^+$] shown in Fig. S1.

3.2 Conjugation of p-xylenediamine with a photo-cleavable group (PC):

p-xylenediamine was reacted with the tert-butyl N-(2-bromoethyl) carbamate in the presence of K_2CO_3 base in dry DMF for 16 h at 45°C to get the Xyl-EDA-Boc. It was further reacted with 2,5-dioxopyrrolidin-1-yl 1-(2-nitrophenyl) ethyl carbonate (PC-ONHS) in the presence of triethylamine base in dry DMF for 16 h at room temperature (25°C) to get Xyl-EDA-PC-Boc. It was then dissolved in formic acid at room temperature (25°C) for 90 minutes to achieve Boc deprotection, yielding the final product Xyl-EDA-PC. The detailed protocol is

described in our recent manuscript available as a bioRxiv preprint (bioRxiv doi: https://doi.org/10.1101/2023.04.24.538015).

3.3 DTX-NH_2 conjugation with p-xylenediamine:

3.3.1 Conjugation of Bis(sulfosuccinimidyl) suberate (BS_3) with DTX-NH_2:

1. DTX-NH_2 (1 mg, 1.414 µmol, dissolved in 40 µl dry DMSO) was taken in a 0.5 ml microcentrifuge tube.
2. Triethylamine (7.88 µl from the stock of 10% (v/v) triethylamine in dry DMSO, 5.656 µmol) was added to it.
3. Bis(sulfosuccinimidyl) suberate (BS_3) (7.48 mg, 14.140 µmol, dissolved in 40 µl dry DMSO) was added to it, and the reaction was stirred at room temperature (25°C) for 30 min.
4. After that, the reaction mixture was diluted using 50 µl of milli-q water and injected into reversed-phase HPLC for purification using water/acetonitrile containing 0.1% TFA as eluent. Yield: 66%
5. After purification, DTX-BS_3 conjugate was characterized by LCMS. LCMS (ESI-MS): Calculated for $C_{50}H_{60}N_{20}S$ 1040.34, found 1041.72 [$M+H^+$] shown in Fig. S2.
6. Given the instability of the NHS compounds, the DTX-BS_3 conjugate was directly used in the next step without delay.

3.3.2 Reaction of BS_3–DTX-NH_2 conjugate with o-nitrobenzyl protected p-xylenediamine guest molecule (Xyl-EDA-PC:

1. The DTX-BS_3 conjugate (0.98 mg) was then dissolved in dry DMSO (80 µl) for conjugation with Xyl-EDA-PC.

2. In a separate microcentrifuge tube, Xyl-EDA-PC (2.63 mg, 7.072 µmol, dissolved in 30 µl DMSO) was taken, and triethylamine (1.97 µl, 14.144 µmol) was added to it.

3. Next, both solutions were mixed together and stirred at room temperature for 12 h.

4. After completion of reactions, the reaction mixture was diluted using 50 µl of milli-q water and injected into reversed-phase HPLC for purification using water/acetonitrile containing 0.1% TFA as eluent. Yield: 30%

5. The HPLC-purified Xyl-EDA-PC conjugated docetaxel (Xyl-DTX-PC) was then characterized by HRMS and ^{1}H–NMR. HRMS (ESI-MS) calculated for $C_{65}H_{80}N_5O_{18}^+$: 1218.5493, found: 1218.5477 [$M+H^+$]. The HRMS and ^{1}H–NMR of Xyl-DTX-PC have been shown in Fig. S3 and S4, respectively.

Xyl-DTX-PC (Figure S4): ^{1}H NMR (DMSO–d_6, 600 MHz) δ 8.38 (d, J=9.0 Hz, 1H), 7.98 (m, 4H), 7.79 (t, J=7.8 Hz,1H), 7.66-7.72 (m, 2H), 7.54-7.62 (m, 2H), 7.32-7.40 (m, 5H), 7.31 (d, J=7.8 Hz, 2H), 7.22 (m, 2H), 5.86-

6.00 (m, 3H), 5.41 (d, J=7.8 Hz, 1H), 5.24-5.29 (m, 1H), 5.09 (d, J=2.4 Hz, 1H), 5.03 (d, J=7.2 Hz, 1H), 4.98 (br, 1H), 4.90 (d, J=10.8 Hz, 1H), 4.58 (br, 1H), 4.41 (t, J=6.0 Hz, 1H), 3.98-4.14 (m, 6H), 3.67 (d, J=7.2 Hz, 1H), 3.27-3.47 (2H, merged with H_2O peak), 2.93 (br, 2H), 2.27 (m, 1H), 2.23 (s, 3H), 2.11-2.19 (m, 2H), 2.02-2.09 (m, 2H), 1.61-2.01 (m, 6H), 1.55 (d, J=6.6 Hz, 3H), 1.52 (s, 3H), 1.39-1.49 (m, 4H), 1.11-1.23 (m, 4H), 0.98-1.02 (s, 6H).

3.3.3 Deprotection of Xyl-DTX-PC to generate Xyl-DTX:

1. The purified Xyl-DTX-PC compound (0.35 mg) was dissolved in 1:1 acetonitrile (50 µl) and milli-q water (50 µl) in a 0.5 ml microcentrifuge tube. The solution was then irradiated with a 365 nm UV lamp (50 mW/cm^2) for 5 min. Subsequently, the photocleaved product was injected into a reverse-phase HPLC for purification using water and acetonitrile containing 0.1% trifluoroacetic acid (TFA) as the mobile phase. Yield: 45%

2. The Xyl-conjugated docetaxel (Xyl-DTX) was then characterized by HRMS and proceeded to the cytotoxicity and imaging experiment. HRMS (ESI-MS) calculated for $C_{56}H_{73}N_4O_{14}^+$: 1025.5118, found: 1025.5069 [M+H$^+$]. The HRMS and ^{1}H–NMR of Xyl-DTX conjugate have been shown in Fig. S5 and S6, respectively. Furthermore, we conducted HPLC analysis to assess the purity of the Xyl-DTX conjugate, as illustrated in Fig. S7.

Xyl-DTX (Fig. S6): ^{1}H NMR (DMSO–d_6, 600 MHz) δ 8.37 (d, J=9.0 Hz, 1H), 7.99 (d, J=7.2 Hz, 2H), 7.69 (t, J=7.2 Hz,1H), 7.60 (t, J=7.2 Hz, 2H), 7.45-7.53 (m, 5H), 7.32 (t, J=7.8 Hz, 2H), 7.32 (d, J=7.8 Hz, 2H), 7.22 (t, J=7.2 Hz, 1H), 5.86-5.97 (m, 2H), 5.42 (d, J=7.8 Hz, 1H), 5.25-5.29 (m, 1H), 5.09 (d, J=3 Hz, 1H), 5.03 (d, J=7.2 Hz, 1H), 4.98-5.00 (br, 1H), 4.91 (d, J=10.2 Hz, 1H), 4.58 (br, 1H), 4.41 (t, J=6.6 Hz, 1H), 4.18 (br, 2H), 3.98-4.08 (m,4H), 3.69 (d, J=7.2 Hz, 1H), 3.24-3.37 (2H, merged with H_2O peak), 2.94 (br, 2H), 2.28 (m, 1H), 2.24 (s, 3H), 2.10-2.19 (m, 2H), 2.01-2.09 (m, 2H), 1.62-1.87 (m, 6H), 1.52 (s, 3H), 1.40-1.49 (m, 4H), 1.12-1.22 (m, 4H), 0.96-1.04 (s, 6H).

4. MALDI-MS characterization of host-guest complexes in water

MALDI-MS characterization of Xyl-DTX, CB[7]·Xyl-DTX and [CB[7]·Xyl-DTX +ADA] in milli-q water:

We used matrix-assisted laser desorption ionization mass spectrometry (MALDI-MS) to look into the formation of supramolecular host-guest complexes between Xyl-DTX and CB[7], as well as the competitive unsheathing of the CB[7]·Xyl-DTX host-guest complexes by ADA.

(i) Xyl-DTX: A final concentration of 10 µM Xyl-DTX was prepared for MALDI-MS analysis. Specifically, 0.80 µl of Xyl-DTX (from a stock solution of 372.51 µM in DMSO) was dissolved in 29.2 µl of Milli-Q water within a 0.5 ml microcentrifuge tube, and this solution was utilized for the MALDI analysis.

(ii) CB[7]·Xyl-DTX: For MALDI-MS analysis of CB[7]·Xyl-DTX, we prepared a final concentration of 10 µM Xyl-DTX in combination with 10 µM CB[7]. Xyl-DTX (0.80 µl from the stock solution of 372.51 µM in DMSO) and CB[7] (1 µl from the stock solution of 300 µM in milli-q water) were dissolved in 28.2 µl milli-q water and kept for complexation for 1 h in 0.5 ml microcentrifuge tube and used for MALDI-MS analysis.

(iii) (CB[7]·Xyl-DTX)+ADA: At first, CB[7]·Xyl-DTX complex was prepared by following the above method. Subsequently, 1-adamantylamine hydrochloride (ADA) (final conc.:10 µM, 1 µl from the stock solution of 300 µM in milli-q water) was added into it and kept for complexation for 15 min and used for the MALDI-MS analysis.

MALDI-MS sample preparation: 2.5 µl of the solution (from (i), (ii), and (iii) above) was deposited onto a 384 MTP ground steel MALDI plate. Subsequently, it was gently blended with 2.5 µl of the MALDI matrix, which is a saturated solution of CHCA in 70% acetonitrile (ACN) and 30% water with 0.1% TFA. The mixture was allowed to air dry before analysis.

MALDI-MS analysis of Xyl-DTX is shown in Fig. S8. MALDI-MS analysis of CB[7]·Xyl-DTX and (CB[7]·Xyl-DTX)+ADA is presented in the main text.

5. Isothermal titration calorimetry (ITC)

Isothermal titration calorimetry (ITC) experiment to calculate the binding affinity between CB[7] and Xyl-DTX:

The isothermal titration calorimetry (ITC) experiment was carried out in a MicroCal PEAQ-ITC calorimeter. 20 µM solution of CB[7] and 200 µM solution of Xyl-DTX/DTX were prepared in 1% DMSO (v/v) in milli-q water. The reference cell of the calorimeter was filled with 1% DMSO (v/v) in milli-q water solution. Then CB[7] solution was placed in the sample cell, and the Xyl-DTX/DTX solution was titrated into the CB[7] solution at 25°C. The automated addition of 2 µl titrant was continued for 19 injections with a time interval of 150 seconds, and the stirring speed was 750 rpm. The duration of each addition of titrant was 4 s. The integrated data were fitted by MicroCal PEAQ-ITC analysis software to get the thermodynamic parameters, using one site binding model built into the software.

The parameters are n (number of binding sites [Xyl-DTX/CB7]) is 0.879 ± 7.9 × 10^{-3}, association constant (k_a) is 1.70 × 10^7 M^{-1} ± 0.51 × 10^7 M^{-1}, obtained ΔH value is -19.0 ± 0.427 kJ/mol, obtained ΔG value is -41.3 kJ/mol and -TΔS is -22.4 kJ/mol. No clear heat change was observed in the case of CB[7] and DTX titration, indicating that the parent DTX molecule does not possess any significant affinity towards CB[7] (Fig. 1b and 1c).

6. Fluorescence titration with CB[7]·berberine complex

5.1 Fluorescence titration of CB[7]·berberine complex with Xyl-DTX/DTX in PBS:

Firstly, We prepared the primary stock solution of berberine (1 mM) in milli-q water, CB[7] (1 mM) in milli-q water, and Xyl-DTX/DTX (1.164 mM) in acetonitrile (ACN). We took berberine (5 µM, 0.5 µl from the stock solution of 1 mM in milli-q water) and CB[7] (25 µM, 2.5 µl from the stock solution of 1 mM in milli-q water) dissolved in 97 µl of 5 mM of phosphate buffer (pH=7.4) solution and added in the one well of a half-well plate of non-binding surface and recorded the fluorescence spectra emission from 430 nm to 700 nm upon excitation at 400 nm wavelength at room temperature. Then we added Xyl-DTX (10 µM, 0.859 µl from the stock solution of 1.164 mM in ACN) in that well, and we observed a significant decrease in fluorescence intensity. Then we gradually increased Xyl-DTX concentration and recorded the fluorescence spectra at 20 µM, 30 µM, and 40 µM. In another well, we titrated CB[7]·berberine complexes with DTX using similar parameters and observed no significant change in the fluorescence intensity. As the Xyl-DTX/DTX solution was prepared in acetonitrile, to subtract the decrease in fluorescence intensity due to ACN contribution, we titrated CB[7]·berberine complexes with ACN by adding 0.859 µl of acetonitrile four times and recorded the fluorescence spectra. We have plotted the normalized fluorescence intensity at 500 nm vs concentration (µM) of DTX and Xyl-DTX added to the CB[7]·berberine complex after subtracting the ACN contribution (Fig. 1d).

5.2 Fluorescence titration of CB[7]·berberine complex in phenol-red free DMEM media (containing 10% FBS):

We initially attempted to replicate the berberine studies in phenol red-free cell culture media. However, as shown in Fig. S9, the inherent emission from the culture media completely masked the berberine emissions. Therefore, using berberine to study the binding of CB[7] with Xyl-DTX in cell culture media was not feasible.

Protocol for sample preparation and analysis: We prepared the primary stock solution of berberine (1 mM) in milli-q water, and CB[7] (5 mM) in milli-q water. We took berberine (5 µM, 1 µl from the stock solution of 1 mM in milli-q water) and CB[7] (25 µM, 1 µl from the stock solution of 5 mM in milli-q water) dissolved in 198 µl of (10 mM PBS (pH=7.4) or phenol-red free DMEM media solution) and added in the two different well of a

half-well plate of non-binding surface and recorded the fluorescence spectra emission from 450 nm to 700 nm upon excitation at 420 nm wavelength at 37°C.

7. Turbidometry Assay.

Sample preparation and kinetic measurement:

1. Buffer solution containing 1 mM GTP diluted in 1× BRB80 was prepared and kept on ice.

2. This buffer solution (77.32 µl, 73.32 µl, 77.32 µl and 100 µl) was added to the four different wells of a 96-well plate kept on ice, followed by the addition of the following solutions:

(i) To the first well, 2.68 µl of Xyl-DTX from the stock solution of 373.13 µM in DMSO was introduced. This was aimed to result in a final concentration of 10 µM within the well.

(ii) To the second well, CB[7]·Xyl-DTX (200 µM CB[7] + 10 µM Xyl-DTX) complex was introduced. Xyl-DTX (2.68 µl from the stock solution of 373.13 µM in DMSO) and CB[7] (4 µl from the stock solution of 5 mM in milli-q water) were kept for complexation in 0.5 ml microcentrifuge tube for 1 h and added to the second well.

(iii) To the third well, 2.68 µl of DMSO was added to the existing solution of the buffer.

3. Tubulin (20 µl from the stock in 1× BRB80) was then added to the first, second, and third wells. The final concentration of the tubulin in the wells was 20 µM. The final volume was maintained at 100 µl of each well. The plate was shifted to the plate reader kept at 37°C. In the third and fourth wells, buffer with and without tubulin was used as controls. A kinetic study was carried out by taking absorbance measurements every 20 s intervals for 40 min at 350 nm wavelength. (Fig. 2a)

Note: Composition of 1× BRB80 buffer: K- PIPES (pH – 6.8) – 80 mM, $MgCl_2$ – 2 mM and EGTA – 1 mM

Effect of CB[7] on the tubulin polymerization kinetics:

We compared the polymerization kinetics of tubulin alone against tubulin incubated with 200 µM (20×) CB[7] to rule out any effect of excess CB[7] on the observed turbidity results. The results, shown in Fig. S10a indicated similar kinetic graphs for tubulin alone and tubulin with CB[7]. This suggests that the polymerization kinetics of tubulin are not affected by the excess CB[7] in solution.

Protocol of Turbidometry Assay:

1. Buffer solution containing 1 mM GTP diluted in 1× BRB80 was prepared and kept on ice.

2. This buffer solution (81 µl) was added to the two different wells of a 96-well plate kept on ice, followed by the addition of the following solutions:

(i) To the first and second wells, 1 µl of DMSO was added to the existing solution of the buffer to make an overall 1% DMSO in both wells.

(ii) To the first well, 4 µl (200 µM) of CB[7] from the stock solution of 5 mM in milli-Q water was added. This was aimed to result in a final concentration of 200 µM within the well.

(iii) To the second well, 4 µl milli-Q water was added to keep a similar condition as in well 1.

3. Tubulin (14 µl from the stock in 1 × BRB80) was then added to the first, second, and third wells. The final concentration of the tubulin in the wells was 20 µM. The final volume was maintained at 100 µl of each well. The plate was shifted to the plate reader kept at 37°C. A kinetic study was carried out once the temperature was stabilized by taking absorbance measurements every 20 s interval for 20 min at 350 nm wavelength.

Effect of CB[7] on the turbidity in 1 × BRB80 buffer:

We compared the turbidity of a 200 µM solution of CB[7] in the assay buffer against the buffer itself to determine if any turbidity arises directly from the CB[7] in the solvent system. The results, shown in Fig. S10b showed no observable difference between the turbidity of the CB[7] solution and the buffer. This indicates that the turbidity is not coming directly from the CB[7] in the solvent system due to solubility issues and is not affecting the measurements.

Protocol of Turbidometry Assay:

1. Buffer solution containing 1 mM GTP diluted in 1 × BRB80 was prepared and kept on ice.

2. This buffer solution (95 µl) was added to the two different wells of a 96-well plate kept on ice, followed by the addition of the following solutions:

(i) To the first and second wells, 1 µl of DMSO was added to the existing solution of the buffer to make an overall 1% DMSO in both wells.

(ii) To the first well, 4 µl (200 µM) of CB[7] from the stock solution of 5 mM in milli-Q water was added. This was aimed to result in a final concentration of 200 µM within the well.

(iii) To the second well, 4 µl milli-Q water was added to keep a similar condition as in well 1.

The final volume was maintained at 100 µl of each well. The plate was shifted to the plate reader kept at 37°C. A kinetic study was carried out once the temperature was stabilized by taking absorbance measurements every 20 s intervals for 20 min at 350 nm wavelength.

8. Computational Methods

8.1 Docking guided by Molecular Dynamics simulation:

The ligand molecules, Xyl-DTX and CB[7]·Xyl-DTX complex, were first simulated in water to obtain an ensemble of ligand conformations. The General AMBER Force Field (GAFF) parameters for DTX and CB[7] were obtained using Antechamber[1] via the graphical interface CHARMM-GUI[2] and the water molecules were modelled using the TIP3P[3] water model. Atomistic molecular dynamics simulations of the solvated ligands were performed for 50 ns using the GROMACS 2020.5[4] simulation package. Using RMSD-based k-means clustering, ten ligand conformations were chosen from the last 25 ns of the equilibrated trajectory. Docking simulations of these ten conformations (5 iterations for each conformation) were performed in the taxol binding site of β-tubulin (PDB ID: 1JFF)[5] using the Autodock Vina program[6], which is widely used for accurate binding-mode predictions of protein-ligand complexes. The root mean squared deviation (RMSD) of the 50 docked poses were calculated using one of the conformations as reference structure.

8.2 Metadynamics:

The ligand conformation corresponding to the highest probability of RMSD is further used for estimating the binding free energy by employing well-tempered metadynamics[7] approach. This method involves adding a bias potential as a function of one or more collective variables (CV) in order to enable the system to effectively sample the configurational space and rescue from being trapped in low free-energy basins of phase space. The free energy surface of the system can be determined from the negative of the accumulated biasing potential. The center of mass distance between the ligand binding pocket and the ligand molecule (d) is used as the CV in the metadynamics run. The binding pocket was defined as the protein residues within a distance of 0.8 nm from the ligand molecule. Here, the bias was added every 500 steps i.e. 1 ps, in the form of a Gaussian function of the CV, defined as

$$V(\vec{s},t) = \sum_{k\tau<t} W(k\tau) exp\left(-\sum_{i=1}^{d} \frac{\left(s_i - s_i\left(q(k\tau)\right)\right)^2}{2\sigma_i^2}\right)$$

where, τ is the Gaussian deposition rate, $W(k\tau)$ is the height of the Gaussian and σ_i is the width of the Gaussian for the *i*th CV. We used a Gaussian height of 1.2 kJ mol^{-1} and a width of 0.035 nm. A bias factor, which is the ratio between the temperature of the CV and the system temperature, with a value of 6.0 was used. The metadynamics simulations were performed in the isothermal-isobaric (NPT) ensemble using GROMACS 2019.6[8] patched with the plugin PLUMED 2.7.0.[9] The CHARMM36 force field[10] was used to model the protein and TIP3P water model was used for the solvent. A timestep of 2 fs was used; a temperature of 300 K was maintained with the Nose-Hoover thermostat[11] and a constant pressure of 1 bar was maintained using the Parrinello-Rahman barostat[12].

The unbound states were defined as the states in which the distance $d < 3.0\,nm$ beyond which the ligand diffuses in the bulk solvent. Three metadynamics simulations were run for each of the systems, Xyl-DTX and CB[7]·Xyl-DTX, until ligand exit from the pocket happens, which varied between 7-12 ns of metadynamics simulation timescale. The average free energy profile from the three simulations was calculated for comparison between the two systems.

8.3 Protein-ligand interactions:

To determine the various interactions stabilizing the binding of the ligands, the Protein-Ligand Interaction Profiler (PLIP) [13] was used to analyze the tubulin-bound conformation of Xyl-DTX and CB[7]·Xyl-DTX.

Supplementary Table S2: Protein-ligand interactions involved in Xyl-DTX binding to tubulin

**Type of interaction**	**Protein residue involved**	
Hydrophobic Interactions		**Distance (nm)**
	**Val-22**	0.367
	**Leu-214**	0.374, 0.365
	**Thr-273**	0.384
	**Leu-283**	0.356
Hydrogen Bonds		**Distance (nm) (donor:acceptor)**
	**Ser-233**	0.318
	**Pro-271**	0.383
	**Arg-275**	0.327, 0.360
	**Arg-358**	0.40
Salt Bridges		**Distance (nm)**
	**Arg-275**	0.497, 0.397

Supplementary Table S3: Protein-ligand interactions involved in CB[7]·Xyl-DTX complex binding to tubulin

**Type of interaction**	**Protein residue involved**	
Hydrophobic Interactions		**Distance (nm)**
	**Leu-216**	0.372
	**Leu-227**	0.367
	**Ala-230**	0.393
	**Phe-269**	0.380
	**Leu-360**	0.361
Hydrogen Bonds		**Distance (nm) (donor:acceptor)**
	**Gln-279**	0.264

9. Fluorescence assay for detection of Xyl binding to CB[7] in phenol-red free DMEM media (containing 10% FBS).

Strategy for fluorescence quenching-based determination of the binding nature of Xyl to CB[7] in phenol-red free DMEM media (containing 10% FBS):

In order to prevent interference with media fluorescence, we developed a displacement study using a red-shifted dye and created a FRET-based quenching assay (Fig. S11). For this purpose, we modified CB[7] with a TAMRA fluorophore and attached the xylene moiety with a FRET quencher BHQ2, which has the ability to quench TAMRA fluorescence when in close proximity. For the FRET assay, we initially prepared a cell culture solution-dispersed CB[7]-TAMRA solution by adding a DMSO stock solution of the dye to phenol red-free DMEM cell culture media containing 10% FBS. Subsequently, we recorded the emission spectra by placing the solution in a 96-well non-binding fluorescence plate and exciting it with 530 nm light. Following this, we introduced a DMSO solution of Xyl-BHQ2 to this solution and recorded the fluorescence spectra once again. As depicted in Fig. S11a, a decrease in TAMRA fluorescence indicated FRET-based quenching due to the proximity between the fluorophore and quencher, suggesting the formation of a CB[7]-Xyl complex in cell culture media. Further control experiments with a quencher conjugate lacking a binding partner for CB[7] (EtA-BHQ2) did not result in any decrease in fluorescence (Fig. S11b). This implies that quenching is triggered by the formation of specific complexes between CB[7] and Xyl. These findings suggest that the Xyl guest moiety can form host-guest complexes with CB[7] in media used for cellular experiments.

Note: The synthesis protocol of CB[7]-TAMRA is reported in our previously published manuscript (A. Som, M. Pahwa, S. Bawari, N. D. Saha, R. Sasmal, M. S. Bosco, J. Mondal and S. S. Agasti, *Chem. Sci.*, 2021, **12**, 5484–5494). Synthesis of Xyl-BHQ2 and EtA-BHQ2 is described in our recent manuscript, available as a bioRxiv preprint (bioRxiv doi: https://doi.org/10.1101/2023.04.24.538015).

Protocol for sample preparation and measurement: Firstly, We prepared the primary stock solution of CB[7]-TAMRA (368.47 µM) in DMSO, Xyl-BHQ2 (77.89 µM) in DMSO and EtA-BHQ2 (97.36 µM) in DMSO. We took CB[7]-TAMRA (1 µM, 0.542 µl from the stock solution) and dissolved it in 200 µl phenol red-free DMEM cell media with 10 % FBS and added in the one well of a 96-well plate of non-binding surface and recorded the fluorescence spectra emission from 560 nm to 700 nm upon excitation at 530 nm wavelength at 37°C. Then, we added Xyl-BHQ2 (1 µM, 2.567 µl from the stock solution, 1eq.) and we observed a decrease in fluorescence intensity. In another well, we added EtA-BHQ2 (1 µM, 2.054 µl from the stock solution, 1eq.) to the (1:1) complex of CB[7]-TAMRA (1 µM) and Xyl-BHQ2 (1 µM), and recorded the fluorescence spectra, and we observed no

significant decrease in fluorescence intensity. These results indicate that host-guest complexation between CB[7]-TAMRA and Xyl-BHQ2 whereas no complexation between CB[7]-TAMARA and EtA-BHQ2.

10. Fluorescence displacement assay for determination of binding of Xyl-DTX with CB[7] in Phenol-red free DMEM media (containing 10% FBS):

Fluorescence displacement study of (1:1) Benz-Cy5 and CB[7]-BHQ3 complex with the addition of Xyl-DTX and DTX in Phenol-red free DMEM media (containing 10% FBS:

To evaluate the Xyl-DTX complexation ability with CB[7] in cell culture media, we employed a quenched Förster Resonance Energy Transfer (FRET) pair comprised of a benzyl guest conjugated to a fluorescent dye (benz-Cy5) and CB[7] conjugated to a quencher (CB[7]-BHQ3). The benzyl guest was selected due to its lower affinity (~10^6 M^{-1}) for CB[7] compared to Xyl-DTX. This lower affinity allows the benzyl guest to be displaced by the higher affinity Xyl-DTX, enabling the study of fluorescence recovery as an indicator of complexation. Initially, we prepared a quenched complex in a microwell plate by mixing benz-Cy5 with CB[7]-BHQ3 in cell culture media (Fig. S12). Following this, a solution of Xyl-DTX was added to this quenched solution. As a control, DTX alone was incubated with the quenched solution in another well. As shown in Fig. 3, fluorescence measurements clearly depicted a recovery of fluorescence in the case of Xyl-DTX addition, whereas the well containing only DTX showed no observable change in fluorescence. This fluorescence recovery indicates that the addition of Xyl-DTX results in the displacement of benz-Cy5 from the CB[7] cavity, confirming the complexation of the Xyl-DTX conjugate with CB[7] in cell culture media.

Sample preparation and measurement protocol: Firstly, We prepared the primary stock solution of Xyl-DTX (1 mM) in DMSO, DTX (1 mM) in DMSO, and CB[7]-BHQ3 (245 µM) in DMSO and benz-Cy5 (245 µM) in DMSO. We took Benz-Cy5 (1 µM, 0.816 µl from the stock solution) dissolved it in 200 µl phenol red-free DMEM cell media and added in the one well of a 96-well plate of non-binding surface and recorded the fluorescence spectra emission from 650 nm to 800 nm upon excitation at 620 nm wavelength in phenol-red free DMEM media at 37°C. Upon the addition of CB[7]-BHQ3 (1 µM, 0.816 µl from stock solution,1eq.), we observed a decrease in fluorescence monitored at the emission wavelength of 665 nm. We observed around 62% quenching at a 1:1 ratio of CB[7] and Benz. To this (1:1) complex of CB[7]-BHQ3 and benz-Cy5, we added Xyl-DTX (10 µM, 2 µl from the stock solution) and recorded the fluorescence spectra. In another well, we titrated CB[7]-BHQ3 (1 µM) and Benz-Cy5 (1 µM) complex with DTX (10 µM, 2 µl from the stock solution) as control using similar parameters and observed no change in the fluorescence intensity.

Note: Synthesis of CB[7]-BHQ3 is reported in our previously published manuscript (A. Som, M. Pahwa, S. Bawari, N. D. Saha, R. Sasmal, M. S. Bosco, J. Mondal and S. S. Agasti, *Chem. Sci.*, 2021, **12**, 5484–5494).

11. MALDI-MS based analysis of CB[7]·Xyl-DTX complexation in phenol-red free DMEM media (containing 10% FBS):

We carried out matrix-assisted laser desorption ionization mass spectrometry (MALDI-MS) analysis using an α-Cyano-4-hydroxycinnamic acid (CHCA) matrix. To determine the supramolecular complex formation, we initiated a complexation reaction by mixing 3 µM of Xyl-DTX with 20 eq. of CB[7] in phenol-red free DMEM media with 10% FBS for 1 h. The MALDI-MS spectra of this mixture displayed an intense mass signature at 2188.00 m/z, corresponding to the 1:1 host-guest complex between CB[7] and Xyl-DTX.

Protocol for sample preparation and MALDI-MS analysis:

Xyl-DTX (0.60 µl from the stock solution of 150 µM in DMSO) and CB[7] (1.8 µl from the stock solution of 1 mM in milli-q water) were dissolved in 27.6 µl phenol-red free DMEM media and kept for complexation for 1 h in 0.5 ml microcentrifuge tube and used for MALDI-MS analysis.

Samples, consisting of 5 µl, were deposited onto a 384 MTP ground steel MALDI plate. Subsequently, it was gently blended with 2.5 µl of the MALDI matrix, which is a saturated solution of CHCA in 70% acetonitrile and 30% water with 0.1% TFA. The mixture was allowed to air dry before analysis.

The MALDI-MS data for the complexation of CB[7] and Xyl-DTX in phenol-red free DMEM media (containing 10% FBS) is presented in Fig. S13.

12. Cell-imaging experiment for detection of Xyl binding to CB[7] in phenol-red free DMEM media (containing 10% FBS).

Cell-imaging experiment to determine the binding nature of Xyl to CB[7] in phenol-red free DMEM media containing 10% FBS:

We performed experiments to support the formation of a 1:1 complex between CB[7] and the Xyl guest in a cellular environment. To achieve this, we mimicked the intracellular environment by immobilizing Xyl onto cellular microtubules using an antibody (Ab)-based labeling approach (via Xyl-conjugated antibody).

Additionally, we conducted the complexation experiment in DMEM cell culture media containing 10% FBS, which is used for the growth of live cells. We then added a CB[7]-FL solution (dispersed in DMEM cell culture media containing 10% FBS) to the cells and acquired fluorescence images. As shown in Fig. S14, the specific lighting up of the microtubule structures confirmed the immobilization of CB[7]-FL on the tubulin through the formation of the CB[7]·Xyl-Ab host-guest complex. This indicates the ability of Xyl to form a 1:1 complex in an intracellular environment.

Protocol for sample preparation and imaging:

Methanol fixation of cells:

1. Culture media was removed from chamber wells and cells were washed twice with 1 × PBS (pH 7.4).

2. Cells were fixed for 7 minutes with chilled methanol at −20°C followed by washing three times with 1 × PBS.

3. Cells were blocked for 2 h with 3% bovine serum albumin in 1× PBS at room temperature.

Cell imaging:

1. BS-C-1 cell was cultured in a humidified atmosphere (5% CO_2) at 37°C and grown in Minimum Essential Medium (MEM) containing (+) non-essential amino acids (NEAA) and no glutamine, which was further supplemented with 10% fetal bovine serum (FBS) (Gibco, USA), 2 mM Glutamax (Invitrogen, USA) and 1% antibiotics (100 U/ml penicillin, 100 μg/ml streptomycin and 0.25 μg/ml amphotericin) (Gibco, USA), 1 mM Sodium Pyruvate (Gibco, USA). BS-C1 cell was a gift from the Nitin Mohan group at IIT-Kanpur (INDIA).

2. BS-C1 cells (~10000/well) were plated in an 8-well imaging plate and, after 24 h, fixed using methanol fixation protocol.

3. Cells were incubated for 36 h at 4°C with primary antibody against tubulin (10 μg/ml, 150 μl) diluted in PBS containing 3% bovine serum albumin in 1× PBS (pH=7.4).

4. Excess primary antibody was removed by washing with 200 μl 1 × PBS thrice with 5 min incubation each time.

5. Cells were incubated at room temperature for 2 h with Xyl conjugated Donkey Anti-Rat secondary antibody (10 μg/ml, 120 μl) in PBS containing 3% bovine serum albumin. The synthesis protocol of Xyl conjugated Donkey Anti-Rat secondary antibody will be a part of our recent manuscript, available as a bioRxiv preprint (bioRxiv doi: https://doi.org/10.1101/2023.04.24.538015).

6. Excess secondary antibody was removed by washing with 200 µl 1× PBS thrice with 5 min incubation each time.

7. Cells were incubated with CB[7]-TAMRA (50 nM) in phenol-red free DMEM media (150 µl) for 15 min and imaged.

13. Cell imaging experiment for detection of Xyl-DTX binding to CB[7] in phenol-red free DMEM media (containing 10% FBS).

Cell imaging experiment for determination of the binding nature of Xyl-DTX to CB[7] in phenol-red free DMEM media containing 10% FBS:

Next, we investigated the intracellular complexation of Xyl-DTX with CB[7]. We designed a displacement assay in cells to gather evidence. Initially, cellular microtubules were labeled with a benzyl guest, which has a relatively lower affinity towards CB[7] compared to the Xyl guest. We used a benzyl guest conjugated antibody (benz-Ab) to label microtubules. Subsequently, incubation with CB[7]-TAMRA formed CB[7]-TAMRA·benz-Ab complex inside the cells, confirmed by the specific visualization of microtubule structures. After forming this CB[7]-FL·benz-Ab host-guest complex, we incubated the cells with Xyl-DTX in one case and with DTX alone in another. As shown in Fig. S15, incubation with Xyl-DTX resulted in a significant decrease in microtubule-specific fluorescence, whereas incubation with DTX alone did not affect the microtubule fluorescence. This indicates that Xyl-DTX is capable of displacing CB[7]-FL from the benzyl guest by forming a more stable CB[7]-FL·Xyl-DTX complex, thereby erasing the fluorescence signal from the microtubules. Control experiments with DTX alone showing no change in fluorescence establish that the specific high affinity of Xyl-DTX towards CB[7] is crucial for the displacement reaction. This supports the hypothesis that the signal erasure is due to the high-affinity complex formation between Xyl-DTX and CB[7], indicating the formation of the CB[7]·Xyl-DTX host-guest complex in an intracellular environment.

Protocol for sample preparation and imaging:

1. BS-C1 cells (~10,000/well) were plated in an 8-well chambered coverglass and, after 24 h, fixed using methanol fixation protocol.

2. Cells were incubated for 36 h at 4°C with primary antibody against tubulin (10 µg/ml, 150 µl) diluted in PBS containing 3% bovine serum albumin in 1× PBS (pH=7.4).

3. Excess primary antibody was removed by washing with 200 µl 1×PBS thrice with 5 min incubation each time.

4. Cells were incubated at room temperature for 2 h with benzyl-conjugated Donkey Anti-Rat secondary antibody (10 µg/ml, 120 µl) in PBS containing 3% bovine serum albumin. (Note: The Synthesis protocol of benzyl-conjugated Donkey Anti-Rat secondary antibody is described below.)

5. Excess secondary antibody was removed by washing with 200 µl PBS thrice with 5 min incubation each time.

6. Cells were incubated with CB[7]-TAMRA (50 nM) in phenol-red free DMEM media (150 µl) for 15 min and imaged.

7. The Xyl-DTX (25 µM, 3.75 µl from the stock solution of 1 mM in DMSO) was added to the cell media in one well. A control DTX (25 µM, 3.75 µl from the stock solution of 1 mM in DMSO) was added to another well.

8. Structured illumination images were then recorded in the 561 nm after 60 min, keeping the laser intensity and exposure time similar.

14. Cellular assays and cell imaging with Xyl-DTX and CB[7]·Xyl-DTX

14.1 General protocol for cell assays and imaging:

1. The cells were cultured in a humidified atmosphere (5% CO_2) at 37°C.
2. At ~80% confluence, the cells were washed with 1× DPBS (pH 7.3) (Gibco, USA), trypsinized, and suspended in the culture medium.
3. Cells were then counted, and then, in a typical experiment, ~10,000 cells/well/100 µL media were plated in a 96-well plate for cytotoxicity study.
4. The cells were then maintained again in a humidified atmosphere (37°C, 5% CO_2) for 24 h to reach ~60% confluence.

Note: 1. HeLa cells expressing GFP-labelled *α- tubulin* were used for cellular imaging experiments.

2. In all the experiments of cellular imaging and cytotoxicity, the compound incubation was done in cell media prepared with 10 µM Verapamil.

3. For the cytotoxicity experiment, 5 mg/ml MTT stock was prepared in 1× PBS (pH=7.4).

4. CB[7] complexation with the Xyl-DTX/ADA/ADA-NPs was done in two different way

(i) *Ex-situ* complexation, in which complexation is performed in a microcentrifuge tube before being fed to the cells.

(ii) *In-situ* complexation in which complexation occurs after being fed to the cells.

14.2 Protocol of cellular imaging of HeLa cells expressing GFP labelled α-tubulin:

14.2.1 Time-lapse imaging (time scale: s) of HeLa cells treated with Xyl-DTX and CB[7]·Xyl-DTX:

1. HeLa cells expressing GFP-labelled α- tubulin were seeded at ~10,000 cells / 150 µl media in two single glass bottom 35 mm dishes (10 mm plating area).

2. After 24 h of plating, cells were washed with 1× DPBS three times and incubated with the following solutions:

(i) Xyl-DTX (final conc: 3 µM, 2.54 µl from the stock solution of 177.16 µM in DMSO) with DMEM-phenol red-free media (147.46 µl) containing 10 µM verapamil in the first dish.

(ii) The second dish was incubated with CB[7]·Xyl-DTX complex (60 µM CB[7] + 3 µM Xyl-DTX). Xyl-DTX (2.54 µl from the stock solution of 177.16 µM in DMSO) and CB[7] (1.8 µl from the stock solution of 5 mM in milli-q water) were dissolved in 25.66 µl of milli-q water and kept for complexation for 1 h *ex-situ* in a microcentrifuge tube. This solution was then mixed with 120 µl DMEM-phenol red-free media containing 10 µM verapamil and added to the second dish.

3. SIM images were acquired with a 488 nm excitation laser after 15 h of compound incubation in the case of Xyl-DTX and after 17 h of compound incubation in the case of CB[7]·Xyl-DTX complex (Fig. 4a and 4d).

14.2.2 Time-lapse imaging (time scale: h) of HeLa cells treated with Xyl-DTX:

1. HeLa cells expressing GFP-labelled *α- tubulin* were seeded at ~20,000 cells / 450 µl cell media in a single glass bottom 35 mm dish (20 mm plating area) for time point study.
2. After 24 h of plating, cells were washed with 1×DPBS and incubated with Xyl-DTX (final conc.: 5 µM, 6.04 µl from the stock solution of 372.51 µM in DMSO) dissolved in DMEM-phenol red-free media (144 µl) containing 10 µM verapamil for time point study.
3. SIM images were acquired with a 488 nm excitation laser at different time points after the incubation of the compound.

(**Note**: In the main text Fig. 4b and S16b, the images were acquired at different time points after 0 h, 3 h, 6 h, and 17 h (left to right).

14.3 Cellular uptake of CB[7]·Xyl-DTX complex:

We evaluated the direct cellular permeability of the CB[7]·Xyl-DTX complex using both LC-MS (liquid chromatography-mass spectrometry) and MALDI-MS (matrix-assisted laser desorption/ionization mass spectrometry) analysis. To achieve this, cells were treated with the CB[7].Xyl-DTX complex for 6.5 h. Following this incubation period, the cells were washed twice with cold DPBS (Dulbecco's Phosphate-Buffered Saline) to remove any extracellular drug complexes. The cells were then harvested by scraping and placed into a microcentrifuge tube for lyophilization. The lyophilized cell mass was subsequently extracted with methanol, and the methanol fraction was analyzed using MALDI-MS and LC-MS. As illustrated in Fig. S17, the MALDI-MS spectra clearly demonstrated the presence of mass peaks corresponding to CB[7] and the CB[7]·Xyl-DTX complex. This observation indicates that the CB[7]·Xyl-DTX complex was successfully internalized by the cells. To further verify the identity of the internalized complexes, LC-MS analysis was performed using an Orbitrap high-resolution mass spectrometer. To further verify the identity of the internalized complexes, LC-MS analysis was performed using an Orbitrap high-resolution mass spectrometer. As shown in Fig. S18, the presence of Xyl-DTX and CB[7]·Xyl-DTX complex was easily observable from the LC-MS chromatogram. While the majority of the CB[7]·Xyl-DTX complexes are expected to be dissociated in the HPLC column, we still detected the presence of CB[7]·Xyl-DTX complex along with Xyl-DTX. Notably, free CB[7] is usually poorly ionized in ESI and, therefore, remains undetected by the mass detector. The observed isotopic distribution further confirmed the presence of the desired compounds, Xyl-DTX, and its complex with CB[7]. Overall, these experiments indicated the successful internalization of the CB[7]·Xyl-DTX complex within the cells, as evidenced by both MALDI-MS and LC-MS analyses.

Protocol for sample preparation and analysis:

1. HeLa cells were seeded at ~10,00,000 cells/4 ml cell media in a 60 mm cell culture dish.

2. After 48 h, the cells were washed with 1× DPBS and incubated for 6.5 h with

Xyl-DTX (10 µM) + CB[7] (200 µM) (2.096 µl from the stock solution of 8.11 mM (Xyl-DTX) in DMSO + 68 µl from the stock solution of 5 mM (CB[7]) in milli-q water) were dissolved in 68 µl milli-q water and kept for complexation for 1 h ex-situ in a microcentrifuge tube. Then 1561.90 µl of DMEM media containing 10 µM verapamil (Xyl-DTX: CB[7] = 10 µM: 200 µM) was added to the dish.

3. After 6.5 h, cells were washed twice with cold DPBS (quick wash).

4. Now, 1 ml DPBS was added to the dish and cells were scrapped using the cell scrapper and immediately this solution containing the cells was lyophilized in a 5 ml microcentrifuge tube.

5. After lyophilization, 400 µl of methanol was added to this tube and vortex and sonicated for 30 min.

6. This solution was transferred in a 1.5 ml centrifuge tube, and it was centrifuged for 10 min at 12k rpm.

7. The supernatant was collected in a tube and evaporated by purging the nitrogen gas and re-dissolved in the 20 µl of methanol to concentrate the solution.

8. Now 5 µl this solution was used for LCMS and MALDI studies.

MALDI-MS sample preparation: 5 µl of sample was mixed with 2.5 µl of the MALDI matrix, which is a saturated solution of CHCA in 70% acetonitrile and 30% water with 0.1% TFA. This mixture was deposited onto a 384 MTP ground steel MALDI plate. The mixture was allowed to air dry before analysis.

LC-MS analysis: LC-MS was carried out in (Dionex Ultimate)3000 UHPLC (Thermo Fisher Scientific) system connected with MS (Q Exactive HF from Thermo Fisher Scientific). The LC-MS sample was prepared by mixing the 5 µl of the methanol fraction with 5 µl milli-q water. Then 5 µl this mixture was injected into the system connected with a C8 column (Hypersil GoldTM C8, particle size 5 µm, Dim. (mm) 100 × 2.1, Part no: 25205-102130, Thermo Fisher Scientific). Water and acetonitrile with 0.1% formic acid were used as a mobile phase. The polarity of acetonitrile varied from 5% to 95 % over the time of 12 min. The Extracted ion chromatograms (XIC) was extracted using the mass of 1025.511±5ppm.

14.4 Time-lapse imaging (time scale: h) of HeLa cells treated with CB[7]·Xyl-DTX:

1. HeLa cells expressing GFP-labelled *α- tubulin* were seeded at ~3500 cells / 150 µl media in a 4-chamber glass bottom 35 mm dish (20 mm bottom well) for time point study.
2. After 24 h of plating, cells were washed with 1 × DPBS and incubated with CB[7]·Xyl-DTX complex (100 µM CB[7] + 5 µM Xyl-DTX). Xyl-DTX + CB[7] (2.01 µl from the stock solution of 372.51 µM in DMSO + 3 µl from the stock solution of 5 mM in milli-q water) dissolved in 25 µl milli-q water and kept for complexation for 1 h *ex-situ* in a microcentrifuge tube. The complex is then mixed with 120 µl DMEM-phenol red-free media containing 10 µM verapamil for time point study.
3. SIM images were acquired with a 488nm excitation laser at different time points up to 22.5 h.

(**Note**: In the main text Fig. 4e and S19, the images were acquired at different time points after 10.5 h, 12.5 h, 20.5 h, and 22.5 h (left to right).

14.5 Protocol for the determination of cytotoxicity of Xyl-DTX and CB[7]·Xyl-DTX:

1. HeLa cells were seeded at ~10,000 cells/100µl cell media in a 96-well plate.

2. After 24 h, the cells were washed with 1×DPBS and incubated for 24 h with the following solutions:

(i) Xyl-DTX (5 µM, 8.60 µl from the stock solution of 372.51 µM in DMSO) dissolved in 640 µl cell media containing 10 µM verapamil and added in three different wells, 100 µl in each. The remaining 340 µl was mixed with 340 µl fresh cell media containing 10 µM verapamil to get the final concentration of 2.5 µM of Xyl-DTX and added in three different wells, 100 µl in each. The remaining 340 µl was mixed with 340 µl fresh cell media containing 10 µM verapamil to get the final concentration of 1.25 µM of Xyl-DTX and added in three different wells, 100 µl in each. This half-dilution step is repeated to get 15 different concentrations.

(ii) Xyl-DTX + CB[7] (8.60 µl from the stock solution of 372.51 µM in DMSO + 12.8 µl from the stock solution of 5 mM in milli-q) dissolved in 78.6 µl milli-q water and kept for complexation for 1 h *ex-situ* in a microcentrifuge tube. It is then mixed with 540 µl cell media containing 10 µM verapamil (Xyl-DTX: CB[7] = 5 µM: 100 µM) and added in three different wells, 100 µl in each. The remaining 340 µl was mixed with 340 µl fresh cell media containing 10 µM verapamil to get the final concentration of 2.5 µM: 50 µM of Xyl-DTX: CB[7] and added in three different wells, 100 µl in each. The remaining 340 µl was mixed with 340 µl fresh cell media containing 10 µM verapamil to get the final concentration of 1.25 µM: 25 µM of Xyl-DTX: CB[7] and added in three different wells, 100 µl in each. This half-dilution step is repeated to get 11 different concentrations.

3. After 24 h, the cells were washed thrice with 1× DPBS, and fresh media was added to the cells.

4. Further, after 24 h, the cells were washed once with 1× DPBS and incubated with media containing MTT solution (90 µl 1 × PBS (pH=7.4) + 10 µl MTT from stock) per well.

5. Post MTT incubation for 2 h, the solution was removed, and 100 µl of DMSO was added to each well.

6. After ~20 min, absorbance was measured at 570 nm (experimental wavelength) and 690 nm (reference wavelength) using a Plate Reader Synergy H1 (BioTek), and cellular viability was determined. Graphpad prism software was used to plot the dose-response curve and IC50 determination. Cellular viability was determined by using the formula shown below:

Percentage of cell viability (%) = (Absorbance$_{570\ nm-690\ nm}$ treatment / Absorbance$_{570\ nm-690\ nm}$ control) × 100 %

Cell viability data from this experiment is shown in Fig. 4f and 4g.

14.6 Determination of cell viability upon treatment with DTX and [CB[7]+DTX]:

1. HeLa cells were seeded at ~10,000 cells/100µl cell media in a 96-well plate.

2. After 24 h, the cells were washed with 1× DPBS and incubated for 24 h with the following solutions:

(i) DTX (6.40 µl from the stock solution of 500 µM in DMSO) was dissolved in 640 µl cell media containing 10 µM verapamil (final conc. = 5 µM) and added in three different wells, 100 µl in each. The remaining 340 µl was mixed with 340 µl fresh cell media containing 10 µM verapamil to get the final concentration of 2.5 µM of DTX and added in three different wells, 100 µl in each. The remaining 340 µl was mixed with 340 µl fresh cell media containing 10 µM verapamil to get the final concentration of 1.25 µM of DTX and added in three different wells, 100 µl in each. This half-dilution step is repeated to get 16 different concentrations.

(ii) DTX + CB[7] (6.40 µl from the stock solution of 500 µM in DMSO + 12.8 µl from the stock solution of 5 mM in milli-q) were dissolved in 80.8 µl milli-q water and kept for complexation for 1 h *ex-situ* in a microcentrifuge tube. It is then mixed with 540 µl cell media containing 10 µM verapamil (DTX: CB[7] = 5 µM: 100 µM) added in three different wells, 100 µl in each. The remaining 340 µl was mixed with 340 µl fresh cell media containing 10 µM verapamil to get the final concentration of 2.5 µM: 50 µM of DTX: CB[7] and added in three different wells, 100 µl in each. The remaining 340 µl was mixed with 340 µl fresh cell media containing 10 µM verapamil to get the final concentration of 1.25 µM: 25 µM of DTX: CB[7] and added in three different wells, 100 µl in each. This half-dilution step is repeated to get 16 different concentrations.

3. After 24 h, the cells were washed thrice and incubated with fresh cell media.

4. After 24 h, the cells were washed once and incubated with media containing MTT solution (90 µl 1× PBS (pH=7.4) + 10 µl MTT from stock) per well.

5. After ~2 h incubation, the MTT solution was removed and 100 µl HPLC grade DMSO was added to each well.

6. After ~15 min, absorbance was measured at 570 nm (experimental wavelength) and 690nm (reference wavelength), and cellular viability was determined by using the formula shown below:

Percentage of cell viability (%) = (Absorbance$_{570\ nm-690\ nm}$ treatment / Absorbance$_{570\ nm-690\ nm}$ control) × 100 %

Cell viability data from this experiment is shown in Fig. S20.

15. Fluorescence-based displacement study of Xyl guest by high-affinity ADA in phenol-red free DMEM media (containing 10% FBS)

Strategy and protocol for fluorescence-based determination of displacement of Xyl guest by high-affinity ADA in phenol-red free DMEM media (containing 10% FBS):

We evaluated whether the CB[7]-Xyl complex can be displaced by high-affinity ADA in cell culture media using a quenched FRET pair, CB[7]-TAMRA and Xyl-BHQ2. First, we prepared a CB[7]-TAMRA-Xyl-BHQ2 complex in cell culture media and recorded its fluorescence emission. Subsequently, ADA was added to the solution, and its emission spectra were recorded, showing a clear increase in TAMRA emission (Fig. S21). In contrast, the addition of a low-affinity cyclohexyl guest did not result in any change in fluorescence from the quenched complex. This observation suggests that the high-affinity guest ADA is capable of displacing Xyl from the CB[7] cavity in cell culture media by forming a CB[7]-ADA complex.

Note: Xyl-BHQ2 is reported in our recent manuscript, available as a bioRxiv preprint (bioRxiv doi: https://doi.org/10.1101/2023.04.24.538015).

Protocol for sample preparation and measurement: Firstly, We prepared the primary stock solution of 1-adamantylamine hydrochloride (1 mM) in milli-q water, cyclohexylamine hydrochloride (1 mM) in milli-q water, and CB[7]-TAMRA (368.47 µM) in DMSO and Xyl-BHQ2 (77.89 µM) in DMSO. We took (1:1) complex of CB[7]-TAMRA (1 µM, 0.542 µl from the stock solution) and Xyl-BHQ2 (1 µM, 2.567 µl from the stock solution, 1eq.) dissolved it in 200 µl phenol red-free DMEM cell media with 10 % FBS and added in the one well of a 96-well plate of non-binding surface and recorded the fluorescence spectra emission from 560 nm to 700 nm upon excitation at 530 nm wavelength at 37°C. Then, we added a high-affinity guest 1-adamantylamine (10 µM, 2 µl from the stock solution) and recorded the fluorescence spectra, and observed a significant increase in the fluorescence intensity. In another well, we titrated (1:1) CB[7]-TAMRA (1µM) and Xyl-BHQ2 (1µM) complex with cyclohexylamine hydrochloride (10 µM, 2 µl from the stock solution) using similar parameters and observed no significant change in the fluorescence intensity.

16. Displacement of Xyl guest by high-affinity ADA in the cellular environment:

To investigate the possibility of Xyl displacement by ADA in a cellular environment, we used cells with microtubules fluorescently labeled via CB[7]-Xyl interaction (see figure below). ADA molecules dispersed in cell culture media were added to these fluorescently labeled cells. As shown in Fig S22, the disappearance of the fluorescence signal from the microtubules indicated that CB[7]-FLs are no longer attached to Xyl, having been displaced by ADA through the formation of a CB[7]-FL·ADA complex. This supports the possibility of displacement of the Xyl guest by ADA in a cellular environment.

Protocol for sample preparation and imaging:

Methanol fixation of cells:

1. Culture media was removed from chamber wells, and cells were washed twice with 1 × PBS (pH 7.4).

2. Cells were fixed for 7 minutes with chilled methanol at −20°C followed by washing three times with 1 × PBS.

3. Cells were blocked for 2 h with 3% bovine serum albumin in 1 × PBS at room temperature.

Cell-imaging:

1. BS-C1 cells (~10000) were plated in an 8-well chambered coverglass and, after 24 h, fixed using methanol fixation protocol.

2. Cells were incubated for 36 h at 4°C /well with primary antibody against tubulin (10 µg/ml, 150 µl) diluted in PBS containing 3% bovine serum albumin in 1× PBS (pH=7.4).

3. Excess primary antibody was removed by washing with 200 µl 1 × PBS thrice with 5 min incubation each time.

4. Cells were incubated at room temperature for 2 h with Xyl conjugated Donkey Anti-Rat secondary antibody (10 µg/ml, 120 µl) in PBS containing 3% bovine serum albumin.

5. Excess secondary antibody was removed by washing with 200 µl 1 × PBS thrice with 5 min incubation each time.

6. Cells were incubated with CB[7]-TAMRA (50 nM) in phenol-red free DMEM media (150 µl) for 15 min and imaged.

7. The higher affinity guest (1-adamantylamine hydrochloride) (50 µM, 7.5 µl from the stock solution of 1 mM in Milli-Q) was added to the cell media.

8. Structured illumination images were then recorded in the 561 nm after 1 h, keeping the laser intensity and exposure time similar before and after ADA addition.

17. MALDI-MS characterization of ADA triggered displacement in phenol-red free DMEM media (containing 10% FBS).

MALDI-MS characterization of ADA added CB[7]·Xyl-DTX complex in phenol-red free DMEM media (containing 10% FBS), showing displacement of Xyl-DTX from the CB[7] cavity:

We studied the displacement of the CB[7]•Xyl-DTX complex by ADA in cell culture media using MALDI-MS spectrometry. To do this, we first prepared the CB[7]•Xyl-DTX complex in cell culture media. Subsequently, ADA was added to the mixture. Analysis of this mixture using MALDI mass spectrometry clearly showed the disappearance of the mass peak corresponding to the CB[7]•Xyl-DTX complex (Fig. S23). Concurrently, a new mass peak corresponding to the CB[7]•ADA complex appeared. This observation suggests that ADA molecules are capable of displacing the CB[7]•Xyl-DTX complex formed in cell culture media, resulting in the formation of a CB[7]•ADA complex.

We carried out matrix-assisted laser desorption ionization mass spectrometry (MALDI-MS) analysis using an α-Cyano-4-hydroxycinnamic acid (CHCA) matrix. We initiated a complexation reaction by mixing 3 µM of Xyl-DTX with 20 eq. of CB[7] in Phenol-red free DMEM media with 10 % FBS for 1 h. Then, we added ADA (20 eq.) to this solution for 30 min and used this solution for MALDI-MS. The MALDI-MS spectra of this mixture displayed an intense mass signature at 1314.20 m/z, corresponding to the host-guest complex between CB[7] and ADA. However, no mass peak was observed corresponding to the CB[7] and Xyl-DTX complex.

Protocol for sample preparation and MALDI-MS analysis:

Xyl-DTX (0.60 µl from the stock solution of 150 µM in DMSO) and CB[7] (1.8 µl from the stock solution of 1 mM in milli-q water) were dissolved in 25.8 µl phenol-red free DMEM media and kept for complexation for 1 h in 0.5 ml microcentrifuge tube. After 1 h, 1-adamantylamine hydrochloride (ADA) (1.8 µl from the stock solution of 1 mM in milli-q water) was added to this solution for 30 min and used for MALDI-MS analysis.

Samples, consisting of 5 µl, were deposited onto a 384 MTP ground steel MALDI plate. Subsequently, it was gently blended with 2.5 µl of the MALDI matrix, which is a saturated solution of CHCA in 70% acetonitrile and 30% water with 0.1% TFA. The mixture was allowed to air dry before analysis.

18. Cellular assays and cell imaging studies upon activation of CB[7]·Xyl-DTX complex with ADA

18.1 Determination of cell viability upon treatment with Xyl-DTX, CB[7]·Xyl-DTX, [CB[7]·Xyl-DTX + ADA] and [CB[7] + ADA]:

1. HeLa cells were seeded at ~10,000 cells/100 µl cell media in a 96-well plate.

2. After 24 h, the cells were washed with 1× DPBS and incubated for 24 h with the following solutions:

(i) Xyl-DTX (2.01 µl from the stock solution of 372.51 µM in DMSO) was dissolved in 300 µl cell culture media containing 10 µM verapamil (final conc.: 2.5 µM) and added in three different wells, 100 µl in each.

(ii) Xyl-DTX + CB[7] (2.01 µl from the stock solution of 372.51 µM in DMSO + 3 µl from the stock solution of 5 mM in milli-q water) were dissolved in 25 µl milli-q water and kept for complexation for 1 h *ex-situ*. Then it is mixed with 270 µl cell media containing 10 µM verapamil (Xyl-DTX: CB[7] = 2.5 µM: 50 µM) and added in three different wells, 100 µl in each.

(iii) Xyl-DTX + CB[7] (4.03 µl from the stock solution of 372.51 µM in DMSO + 6 µl from the stock solution of 5 mM in milli-q water) were dissolved in 49.97 µl milli-q water and kept for complexation for 1 h *ex-situ* in a microcentrifuge tube. Then it is mixed with 540 µl cell media containing 10 µM verapamil (Xyl-DTX: CB[7] = 2.5 µM: 50 µM) and added in six different wells, 100 µl in each. 1-adamantylamine hydrochloride (ADA) (final conc.:50 µM, 1 µl from the stock solution of 5 mM in milli-q water) was added *in situ* at 0 h in three different wells and at 2 h in three different wells.

(iv) CB[7] + ADA (16 µl from the stock solution of 5 mM in milli-q water + 16 µl from the stock solution of 5 mM in milli-q water) were mixed and kept for complexation for 1 h *ex-situ* in a microcentrifuge tube. It was then mixed with 608 µl cell media containing 10 µM verapamil (CB[7]: ADA = 125 µM: 125 µM) and added in three different wells, 100 µl in each. The remaining 340 µl was mixed with 340 µl fresh cell media containing 10 µM verapamil to get the final concentration (62.50 µM + 62.50 µM) of CB[7] + ADA and added in three different wells, 100 µl in each. The remaining 340 µl was mixed with 340 µl fresh cell media containing 10 µM verapamil to get the final concentration (31.25 µM + 31.25 µM) of CB[7] + ADA and added in three different wells, 100 µl in each.

(v) ADA (final conc.: 125 µM, 16 µl from the stock solution of 5 mM in milli-q water) was dissolved in 624 µl cell media containing 10 µM verapamil and added in three different wells, 100 µl in each. The remaining 340 µl

was mixed with 340 µl fresh cell media containing 10 µM verapamil to get the final concentration (62.50 µM) and added in three different wells, 100 µl in each. The remaining 340 µl was mixed with 340 µl fresh cell media containing 10 µM verapamil to get the final concentration (31.25 µM) and added in three different wells, 100 µl in each.

3. After 24 h, the cells were washed thrice with 1× DPBS, and fresh media was added to the cells.

4. Further, after 24 h, the cells were washed once with 1× DPBS and incubated with media containing MTT solution (90 µl 1 × PBS (pH=7.4) + 10 µl MTT from stock) per well.

5. Post MTT incubation for 2 h, the solution was removed and 100 µl of DMSO was added to each well.

6. After ~20 min, absorbance was measured at 570 nm (experimental wavelength) and 690 nm (reference wavelength) using a Plate Reader Synergy H1 (BioTek), and cellular viability was determined by using the formula shown below:

$$\text{Percentage of cell viability (\%)} = (\text{Absorbance}_{570\text{ nm}-690\text{ nm}}\ \text{treatment} / \text{Absorbance}_{570\text{ nm}-690\text{ nm}}\ \text{control}) \times 100\ \%$$

Cell viability data from this experiment is shown in Fig. 5b and 5c.

18.2 Protocol of cellular imaging of HeLa cells expressing GFP labelled α-tubulin upon treatment with Xyl-DTX, CB[7]·Xyl-DTX, [CB[7]·Xyl-DTX + ADA] and [CB[7] + ADA]:

1. HeLa cells expressing GFP-labelled *α- tubulin* were seeded at ~3500 cells / 150 µl media in four different single glass bottom 35 mm dishes (10 mm plating area). (For the comparison with control)

2. After 24 h of plating, cells were washed with 1× DPBS and incubated with the following solutions:

(i) Xyl-DTX (2.01 µl from the stock solution of 372.51 µM in DMSO) was mixed with DMEM-phenol red-free media (final conc.: 5 µM) containing 10 µM verapamil and added in the first dish.

(ii) Xyl-DTX + CB[7] (2.01 µl from the stock solution of 372.51 µM in DMSO + 3 µl from the stock solution of 5 mM in milli-q water) were dissolved in 25 µl milli-q water and kept for complexation for 1 h *ex-situ* in a microcentrifuge tube. It is then mixed with 120 µl DMEM-phenol red-free media (Xyl-DTX: CB[7] = 5 µM: 100 µM) containing 10 µM verapamil and added to the second dish.

(iii) Xyl-DTX + CB[7] (2.01 µl from the stock solution of 372.51 µM in DMSO + 3 µl from the stock solution of 5 mM in milli-q water) were dissolved in 25 µl milli-q water and kept for complexation for 1 h *ex-situ* in a microcentrifuge tube. It is then mixed with 120 µl DMEM-phenol red-free media (Xyl-DTX: CB[7] = 5 µM: 100

μM) containing 10 μM verapamil and added to the third dish. 1-adamantylamine hydrochloride (ADA) (final conc.: 100 μM, 3 μl from the stock solution of 5 mM in milli-q water) was added *in situ* at 0 h in the dish directly.

(iv) CB[7] + ADA (3 μl from the stock solution of 5 mM in milli-q water + 3 μl from the stock solution of 5 mM in milli-q water) were dissolved in 24 μl milli-q water and kept for complexation for 1 h *ex-situ* in a microcentrifuge tube. It is then mixed with 120 μl DMEM-phenol red-free media (CB[7]: ADA = 100 μM: 100 μM) containing 10 μM verapamil and added to the fourth dish.

3. SIM images were acquired with a 488 nm excitation laser after incubation of the compound (Fig. 5d and S24).

19. Experiments with ^{C}ADA

19.1 Protocol of fluorescence titration of CB[7]-Bodipy and Xyl-BHQ1 complex with ^{C}ADA :

We carried out the titration of *p*-xylenediamine conjugated BHQ1 (1 μM) with increasing concentration of CB[7] conjugated BODIPY in a 96 well plate in PBS buffer (10 mM, pH= 7.4). Forster Resonance Energy Transfer (FRET) from BODIPY-fluorophore to BHQ1 upon excitation at 460 nm leads to the fluorescence quenching of BODIPY upon formation of the CB[7]-Xyl inclusion complex and recorded the fluorescence spectra emission from 500 nm to 600 nm at room temperature. Upon titrating increasing amounts of the quencher, we observed greater than 92% quenching in fluorescence intensity obtained upon adding 1 equivalent of Xyl-BHQ1. Further, we found no change in the fluorescence of BODIPY fluorophore by the ^{C}ADA addition (5 μM). After 90 s UV irradiation (50 mW/cm^2), we analyzed the recovery in fluorescence of BODIPY fluorophore. Fig. 6b shows the regeneration of BODIPY fluorescence by the light-activated generation of higher affinity guest ADA. This indicates the ability of the caged ADA (^{C}ADA) to enable functional displacement of xylene-conjugated BHQ1 from CB[7] via host-guest interactions upon light activation.

Note: The data presented in Figure 5b was corrected for the photobleaching of BODIPY fluorescence induced by light irradiation. The extent of photobleaching was assessed by subjecting only the BODIPY conjugated CB[7] to the same irradiation duration (90 seconds of UV irradiation at an intensity of 50 mW/cm^2).

CB[7]-Bodipy conjugation:

CB[7]-NH_2 was reacted with NHS ester of Bodipy in the presence of triethylamine base in dry DMSO for 6 h at room temperature and then purified using reversed-phase HPLC. The detailed protocol is reported in our previously published manuscript.[14]

Xyl-BHQ1 conjugation:

Xyl-EDA-PC was reacted with NHS ester of BHQ1 in the presence of triethylamine in dry DMSO for 12 h at room temperature to get the Xyl-PC-BHQ1. The PC group-protected Xyl-BHQ1 was dissolved in a 1:1 water/acetonitrile mixture and irradiated with 365 nm UV light (50 mW/cm^2) for 5 min and purified using reversed-phase HPLC to get the final product Xyl-BHQ1. The detailed protocol is reported in a part of our recent manuscript, available as a bioRxiv preprint (bioRxiv doi: https://doi.org/10.1101/2023.04.24.538015).

19.2 Determination of cell viability upon treatment with Xyl-DTX, CB[7]·Xyl-DTX, [CB [7]·Xyl-DTX + ^{C}ADA] with and without light, and CB [7] + ^{C}ADA with and without light:

1. HeLa cells were seeded at ~10,000 cells/100 µl cell media in a 96-well plate.

2. After 24 h, the cells were washed with 1× DPBS and incubated for 24 h with

(i) Xyl-DTX (final conc.: 3 µM, 0.96 µl from the stock solution of 1 mM in DMSO) dissolved in 320 µl cell media containing 10 µM verapamil and added in three different wells 100 µl in each.

(ii) Xyl-DTX + CB [7] (0.96 µl from the stock solution of 1 mM (Xyl-DTX) in DMSO + 3.84 µl from the stock solution of 5 mM (CB [7]) in milli-q water) were dissolved in 25.2 µl milli-q water and kept for complexation for 1 h *ex-situ* in a microcentrifuge tube. It is then mixed with 290 µl cell media containing 10 µM verapamil (Xyl-DTX: CB [7] = 3 µM: 60 µM) and added in three different wells, 100 µl in each.

(iii) Xyl-DTX + CB [7] (0.96 µl from the stock solution of 1 mM (Xyl-DTX) in DMSO + 3.84 µl from the stock solution of 5 mM (CB [7]) in milli-q water) were dissolved in 25.2 µl milli-q water and kept for complexation for 1 h *ex-situ* in a microcentrifuge tube. It is then mixed with ^{C}ADA (5.55 µl from the stock concentration of 3.46 mM in DMSO) for 30 min. Then 284.45 µl cell media containing 10 µM verapamil (Xyl-DTX: CB [7]: ^{C}ADA = 3 µM: 60 µM: 60 µM) and added in three different wells.

(iv) Xyl-DTX + CB [7] (0.96 µl from the stock solution of 1 mM (Xyl-DTX) in DMSO + 3.84 µl from the stock solution of 5 mM (CB [7]) in milli-q water) were dissolved in 25.2 µl milli-q water and kept for complexation for 1 h *ex-situ* in a microcentrifuge tube. It is then mixed with ^{C}ADA (5.55 µl from the stock concentration of 3.46 mM in DMSO) for 30 min. This solution was irradiated with a UV lamp (50 mW/cm^2) for 180 sec. Then 284.45 µl cell media containing 10 µM verapamil (Xyl-DTX: CB [7]: ^{C}ADA = 3 µM: 60 µM: 60 µM) and added in three different wells.

(v) ^{C}ADA + CB [7] (5.55 µl from the stock solution of 3.46 mM (^{C}ADA) in DMSO + 3.84 µl from the stock solution of 5 mM (CB [7]) in milli-q water) dissolved in 20.61 µl milli-q water and kept for complexation for 1 h *ex-situ* in a microcentrifuge tube. It is then mixed with 290 µl cell media containing 10 µM verapamil (^{C}ADA: CB [7] = 60 µM: 60 µM) and added in three different wells, 100 µl in each.

(vi) ^{C}ADA + CB [7] (5.55 µl from the stock solution of 3.46 mM (^{C}ADA) in DMSO + 3.84 µl from the stock solution of 5 mM (CB[7]) in milli-q water) dissolved in 20.61 µl milli-q water and kept for complexation for 1 h *ex-situ* in a microcentrifuge tube. This solution was irradiated with a UV lamp (50 mW/cm^2) for 180 sec. It is then mixed with 290 µl cell media containing 10 µM verapamil (^{C}ADA: CB[7] = 60 µM: 60 µM) and added in three different wells, 100 µl in each.

(vii) For control 6.51 (0.96 +5.55) µl of DMSO was mixed with 313.49 µl of cell media containing 10 µl verapamil and added in three different wells, 100 µl in each.

3. After 24 h, the cells were washed thrice and incubated with DMEM complete cell media.

4. After 24 h, the cells were washed once and incubated with media containing MTT solution (90 µl 1× PBS (pH=7.4) + 10 µl MTT from stock) per well.

5. After ~2 h incubation, the MTT solution was removed and 100 µl HPLC grade DMSO was added to each well.

6. After ~15 min, absorbance was measured at 570 nm (experimental wavelength) and 690 nm (reference wavelength), and cellular viability was determined by using the formula shown below:

$$\text{Percentage of cell viability (\%)} = (\text{Absorbance}_{570\text{ nm-}690\text{ nm}}\ \text{treatment}/\text{Absorbance}_{570\text{ nm-}690\text{ nm}}\ \text{control}) \times 100\ \%$$

Cell viability data from this experiment is shown in Fig. 6c.

19.3 Effect of light irradiation on live cells

1. HeLa cells were seeded at ~10,000 cells/100 µl cell media in two 96-well plates.

2. After 24 h, the cells were washed with 1× DPBS, and the fresh cell media was added to the wells (a total of seven wells).

3. In one plate, cells were irradiated with a hand-held UV tube, and another plate was kept without UV irradiation. Given that greater surface area irradiation was required, we used a 75W 365 nm UV tube for this purpose. Given its higher power, 120 s irradiation was used.

3. After 24 h, the cells were washed thrice with DPBS and incubated with DMEM complete cell media.

4. After 24 h, the cells were washed once and incubated with media containing MTT solution (90 µl 1× PBS (pH=7.4) + 10 µl MTT from stock) per well.

5. After ~2 h incubation, the MTT solution was removed and 100 µl HPLC grade DMSO was added to each well.

6. After ~15 min, absorbance was measured at 570 nm (experimental wavelength) and 690 nm (reference wavelength), and cellular viability were determined by using the formula shown below:

$$\text{Percentage of cell viability (\%)} = (\text{Absorbance}_{570\ \text{nm-}690\ \text{nm}}\ \text{treatment}/\text{Absorbance}_{570\ \text{nm-}690\ \text{nm}}\ \text{control}) \times 100\ \%$$

Cell viability data from this experiment is shown in Fig. S25.

19.4 Protocol of cellular imaging of HeLa cells expressing GFP labelled α-tubulin upon treatment with Xyl-DTX, CB[7]·Xyl-DTX, [CB[7]·Xyl-DTX + ^{C}ADA] and [CB[7] + ^{C}ADA], with and without light:

1. HeLa cells expressing GFP-labelled α- tubulin were seeded at ~8000 cells / 150 µl media in two different 4-chamber glass bottom 35 mm dishes with 20 mm bottom well. (For the comparison with control).

2. After 24 h of plating, cells were washed with 1× DPBS and incubated with the following solutions:

(i) Xyl-DTX (final conc.:3 µM, 1.21 µl from the stock solution of 372.51 µM in DMSO) with DMEM-phenol red-free media containing 10 µM verapamil in the first well.

(ii) Xyl-DTX + CB[7] (1.21 µl from the stock solution of 372.51 µM in DMSO + 0.90 µl from the stock solution of 5 mM in milli-q water) were dissolved in 27.89 µl milli-q water and kept for complexation for 1 h *ex-situ* in a microcentrifuge tube. It is then mixed with 120 µl DMEM-phenol red-free media (Xyl-DTX: CB[7] = 3 µM: 30 µM) containing 10 µM verapamil and added to the second well.

(iii) Xyl-DTX + CB[7] (1.21 µl from the stock solution of 372.51 µM in DMSO + 0.90 µl from the stock solution of 5 mM in milli-q water) were dissolved in 27.89 µl milli-q water and kept for complexation for 1 h *ex-situ* in a microcentrifuge tube. It is then mixed with 120 µl DMEM-phenol red-free media (Xyl-DTX: CB[7] = 3 µM: 30 µM) containing 10 µM verapamil and added to the third well. ^{C}ADA (final conc.=25 µM, 2.16 µl from the stock solution of 1.736 mM in DMSO) was added *in situ* at 0 h in the dish directly.

(iv) ^{C}ADA + CB[7] (2.16 µl from the stock solution of 1.736 mM in DMSO + 0.90 µl from the stock solution of 5 mM in milli-q water) were dissolved in 26.94 µl milli-q water and kept for complexation for 1 h *ex-situ* in a

microcentrifuge tube. Then it was mixed with 120 µl DMEM-phenol red-free media (^{C}ADA: CB[7]= 25 µM: 50 µM) containing 10 µM verapamil and added to the fourth well.

Note: We prepared two dishes with the above-mentioned compositions. One dish was used for the control study, no UV irradiation. In the second dish, after 2 h of compound incubation, all four wells were irradiated with UV light (50 mW/cm^2) for 100 s.

3. SIM images were acquired with a 488 nm excitation laser after incubation of the compound (Fig. 6d and S26).

20. Synthesis and experiments with ADA-NP

20.1 Synthesis of PC-ADA-PEG$_2$-OH (^{C}ADA):

Tetra ethylene glycol (TEG) was reacted with tosyl chloride in the presence of triethylamine base in acetonitrile for 14 h at room temperature. The product formed (TEG-OTs) was reacted with tert-butyldimethylsilyl chloride (TBDMSCl) in the presence of imidazole base in dry DMF for 24 h at room temperature. Next, the product formed (TBDMS-TEG-OTs) was reacted with 1-aminoadamantane hydrochloride (ADA) in the presence of K_2CO_3 base in DMF for 48 h at 85°C. The product formed (TBDMS-TEG-ADA) was further reacted with PC-ONHS in the presence of triethylamine in dry DCM for 12 h at room temperature to get PC-ADA-TEG-TBDMS. It was dissolved in methanol and amberlyst-15 was added to it for 6 h at room temperature to get the final product **PC-ADA-PEG$_2$-OH** (^{C}ADA). The detailed protocol is reported in our previously published manuscript. [15]

20.2 Synthesis of PC-ADA-PEG$_2$-NHS:

PC-ADA-PEG$_2$-OH (^{C}ADA) was reacted with N, N'- Disuccinimidyl carbonate in the presence of triethylamine in dry acetonitrile for 12 h at room temperature to get the PC-ADA-TEG-NHS. Then it was reacted with amino-PEG6-carboxylic acid in the presence of triethylamine in dry DMF for 12 h at room temperature to get the PC-ADA-PEG$_2$-OH. Further, it was reacted with N, N'- Disuccinimidyl carbonate in the presence of triethylamine in dry DMF for 12 h at room temperature and purified using reversed-phase HPLC to get the final product PC-ADA-PEG$_2$-NHS. It was used for the post-functionalization of gold nanoparticles. The detailed protocol of PC-ADA-PEG$_2$-NHS and Au-NMe$_2$-Prp-NH$_2$ is reported in our previously published manuscript. [15,16]

20.3 Synthesis protocol for preparation of ADA-NP via post-synthesis functionalization of Au-NMe$_2$-Prp-NH$_2$ gold nanoparticles:

The gold nanoparticle was synthesized in-house using the following three steps:

Step 1: Synthesis of pentane thiol-coated gold nanoparticle: Gold nanoparticles of approximately 2 nm in diameter were synthesized using the Brust-Schiffrin method (Reference: M. Brust, M. Walker, D. Bethell, D. J. Schiffrin, and R. Whyman, *J. Chem. Soc., Chem. Commun.*, 1994, **0**, 801–802).

Step 2: Functionalization of nanoparticle with amine-terminated ligand: The gold nanoparticles were then surface functionalized with an amine-terminated thiol ligand using the Murray place exchange reaction (Reference: M. J. Hostetler, S. J. Green, J. J. Stokes, and R. W. Murray, *J. Am. Chem. Soc.*, 1996, **118**, 4212–4213). The synthesis of the amine-terminated thiol ligand and the procedure for the place exchange reaction have been reported in our earlier study (M. Pahwa, P. Jain, N. Das Saha, C. Narayana, and S. S. Agasti, *Chem. Commun.*, 2021, **57**, 9534–9537). This nanoparticle, which is surface functionalized with the amine-terminated ligand, is referred to as $AuNMe_2PrpNH_2$ and is depicted in Supplementary Scheme S1.

Step 3: Formation of ADA-NP: The amine functionalities of the $AuNMe_2PrpNH_2$ were reacted with NHS-functionalized ADA moiety to create the final ADA-NP. Details of the reaction and purification are provided below.

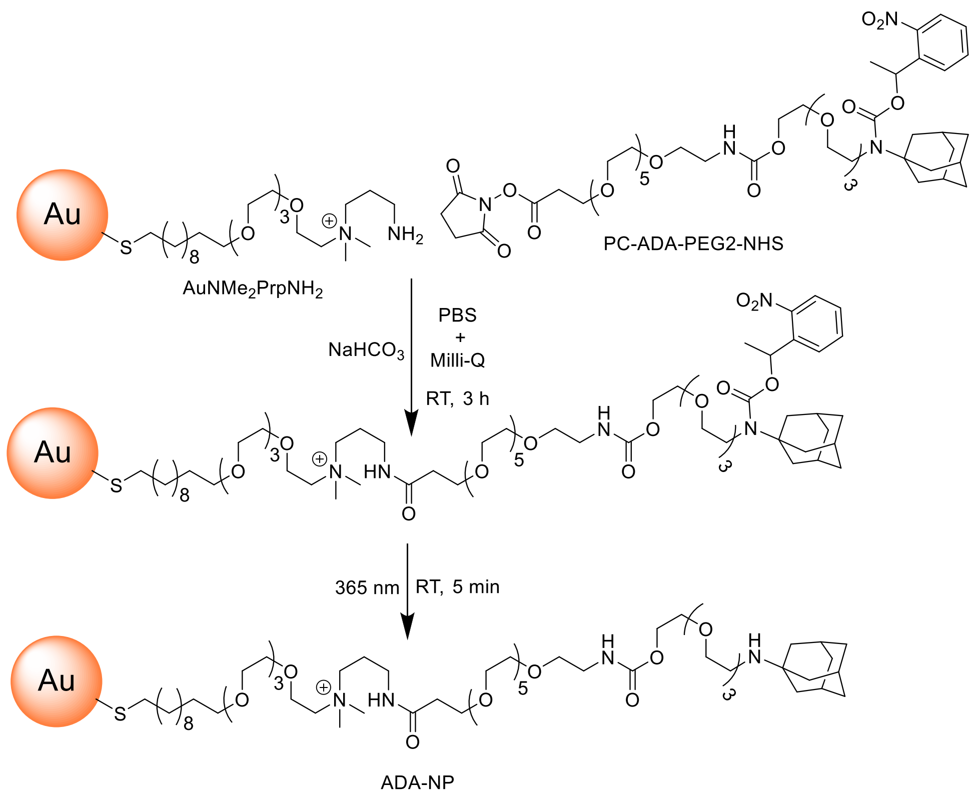

Supplementary Scheme S1. Synthesis scheme for the preparation of ADA functionalized Au-NMe_2-Prp-NH_2.

$AuNMe_2PrpNH_2$ gold nanoparticle (~2 nm core diameter) (100 µl, from 35 µM stock in water) was taken in a 1.5 ml microcentrifuge tube. To this, 10 µl of $NaHCO_3$ (1 M in 1 × PBS buffer (pH = 7.4)) was added and vortexed. After this, PC-ADA-PEG_2-NHS (5 µl, 150 mM solution in dry DMF, 250 eq) solution was added to the nanoparticle solution. After addition, the mixture was vortexed. The resultant mixture was stirred for 3 h at RT. The conjugated nanoparticles were irradiated with UV light (365 nm, 50 mW/cm^2) for 5 min and then purified by a 50 kDa molecular weight cut-offs (MWCO) filter by washing three times with milli-q water. The obtained ADA-functionalized gold-nanoparticles (ADA-NPs) were characterized by various methods as described below.

20.4 Characterization of ADA-NP:

We have conducted a comprehensive characterization of ADA-NP using Transmission Electron Microscopy (TEM) and Dynamic Light Scattering (DLS). This characterization was crucial for understanding the physical properties of the nanoparticles. The TEM images of ADA-NP, shown in Fig. S27a, reveal the detailed morphology and size of the nanoparticles. From these images, we estimated the average core size (diameter, d) of the nanoparticles to be 2.03±0.55 nm (Fig. S27b). The hydrodynamic diameter of ADA-NP was measured using DLS, resulting in an average value of 6.84±2.71 nm (Fig.7b). This measurement includes the nanoparticle core as well as the surface grafted ligands, providing a comprehensive view of the nanoparticle's size in a hydrated state. The DLS data, which is included in the main text, complements the TEM analysis by offering insight into the behavior of the nanoparticles in solution and their potential for aggregation. MALDI-MS analysis confirmed the presence of ADA-conjugated ligands, showing a peak at 1153.95 m/z corresponding to the molecular ion peak of the ADA-appended surface ligand (Fig. 7c).

Protocol for sample preparation and analysis:

TEM studies. Nanoparticles were drop cast on a TEM (carbon-coated copper grid) and dried using the infrared drying method. Then TEM images were captured after 2 h. Transmission Electron Microscopy (TEM) was performed on JEOL JEM-3010 operating at 300 kV.

DLS studies. The hydrodynamic diameter of the Au-ADA-NPs were measured by dynamic light scattering (DLS) in 10 mM phosphate buffer (pH=7.4). DLS experiment was performed using a Malvern Zetasizer instrument. DLS measurements were analyzed in the Zetasizer Xplorer software and GraphPad Prism software. GraphPad Prism was used to plot the data.

MALDI-MS studies. 3 µl of the solution (from 7.05 µM stock in milli-q water) was deposited onto a 384 MTP ground steel MALDI plate. Subsequently, it was gently blended with 2.5 µl of the MALDI matrix, which is a saturated solution of CHCA in 70% acetonitrile and 30% water with 0.1% TFA. The mixture was allowed to air dry before analysis. MALDI-MS: Calculated $C_{58}H_{113}N_4O_{16}S^+$ for ADA-conjugated ligand:1153.79, Found:1153.95 [M^+].

Average number of ADA groups per nanoparticle: To determine the number of adamantane (ADA) molecules per nanoparticle, we devised an assay leveraging the gold core's ability to quench dye fluorescence. This quenching occurs when the fluorophore is in close proximity to the gold core, specifically when it is surface-bound, due to various energy transfer mechanisms. Accordingly, we conducted a fluorescence quenching titration using a CB[7]-conjugated fluorophore (CB[7]-FL). In this assay, the CB[7]-FL solution was titrated with an ADA-NP solution. Our hypothesis was that the binding of CB[7]-FL to the surface-appended ADA moiety would result in fluorescence quenching due to the close proximity of the fluorophore to the gold core. This quenching phenomenon could then be used to calculate the number of ADA moieties per nanoparticle. Accordingly, CB[7]-FL solution was titrated with an ADA-NP solution where increasing amounts of ADA-NP were added to the CB[7]-FL solution, and the fluorescence emission from the fluorophore was measured after each addition. As a control, ADA-NP was added to a solution of a control fluorophore (EtA-FL) that does not contain a CB[7] moiety. The addition of ADA-NP to the CB[7]-FL solution resulted in immediate fluorescence quenching (Fig. S28). In contrast, the control experiment with EtA-FL showed no fluorescence change, confirming that the quenching is due to the specific binding of CB[7] to the ADA moieties. As we increased the concentration of ADA-NP, the fluorescence quenching increased, eventually saturating at an ADA-NP to CB[7] dye ratio of 1:60. This saturation indicates the complete occupancy of ADA binding sites by CB[7]-FL, suggesting that each nanoparticle has approximately 60 ADA ligands.

Evidence that ADA is attached to the nanoparticle surface: To purify the nanoparticle conjugate after the ADA-NHS coupling reaction, we performed extensive washing using a 50 kDa Amicon ultracentrifugal filter. The nanoparticles were washed 3 times with water to remove any unconjugated ADA molecules. The attachment of ADA molecules to the nanoparticles is supported by the observed fluorescence quenching when the nanoparticle complex interacts with CB[7]-FL. This quenching can only occur if ADA is close to the gold core, indicating attachment to the surface ligand. To further support this observation, we conducted a control experiment to determine whether ADA molecules in solution, but not attached to the surface ligand, could also cause this effect. We used the precursor propylamine nanoparticle and added 60 equivalents of ADA-amine, resulting in a

nanoparticle solution containing non-surface-attached ADA molecules. Titration of CB[7]-FL with this nanoparticle solution did not result in any significant change in fluorescence intensity (Fig. S28). This validates that surface-attached ADA is critical for fluorescence quenching. Additionally, MALDI-MS data from the ADA-NP indicated the presence of only ADA-conjugated surface ligands, formed via the amine-NHS reaction. The absence of ADA-NHS reactant or its hydrolyzed product further eliminated the possibility of any free ADA being present.

Firstly, We prepared the primary stock solution of CB[7]-TAMRA (64 µM) in DMSO, EtA-TAMRA (64 µM) in DMSO, and Au-NMe2-Prp-PEG2-ADA (0.674 µM) in milli-q water.[14] We took CB[7]-TAMRA (500 nM, 1.562 µl from the stock solution) and dissolved them in 200 µl of 1×PBS (pH=7.4). and added in the one well of a 96-well plate of non-binding surface and recorded the fluorescence spectra emission from 560 nm to 700 nm upon excitation at 530 nm wavelength. Then, we added Au-ADA-NPs (2.5 nM, 0.742 µl from the stock solution) and recorded the fluorescence spectra. We continued this experiment by stepwise addition of 2.5 nM of Au-ADA-NPs up to 30 nM and observed a significant decrease in the fluorescence. From the plotted graph, we can see that fluorescence is saturating around 12-13 nM concentration of ADA functionalized gold nanoparticles indicating a total of around 60 number ADA attached to per nanoparticles. In another well we have taken EtA-TAMRA (500 nM) and similar way we titrated with Au-ADA-NPs and observed no change in the fluorescence.

In another control experiment, we prepared the primary stock solution of CB[7]-TAMRA (64 µM) in DMSO, and Au-Prp-NH_2 + 60 eq. ADA (0.674 µM + 40.44 µM) respectively in milli-q water. We dissolved CB[7]-TAMRA (500 nM, 1.562 µl from the stock solution) in 200 µl of 1×PBS (pH=7.4), and added in the one well of a 96-well plate of non-binding surface, then recorded the fluorescence spectra emission from 560 nm to 700 nm upon excitation at 530 nm wavelength. Then, we added Au-Prp-NH_2-NPs + 60eq. ADA (2.5 nM + 150 nM, 0.742 µl from the stock solution) and recorded the fluorescence spectra. We continued this experiment by stepwise addition of (2.5 nM + 150 nM) of Au-Prp-NH_2-NPs + 60 eq. ADA up to (30 nM + 1.8 µM) and observed no decrease in the fluorescence. This experiment indicates that if ADA is not functionalized to the gold nanoparticle, it cannot quench the fluorescence of CB[7]-TAMRA.

20.5 Protocol of fluorescence titration of CB[7]-BHQ3 and Xyl-SiR complex with ADA-NPs:

We carried out the titration of *p*-xylenediamine conjugated Silicorhodamine fluorophore (Xyl-SiR) (1 µM) with increasing concentration of CB[7] conjugated BHQ3 (Black hole quencher 3) in a 96 well plate in PBS buffer (10 mM, pH= 7.4). Forster Resonance Energy Transfer (FRET) from SiR-fluorophore to BHQ3 upon excitation at 620 nm leads to the fluorescence quenching of SiR upon formation of the CB[7]-Xyl inclusion complex and recorded the fluorescence spectra emission from 650 nm to 750 nm at room temperature. Upon titrating increasing

amounts of the quencher, we observed greater than 90% quenching in fluorescence intensity was obtained upon the addition of 1.5 equivalents of CB[7]-BHQ3. Further, we analyzed the recovery in fluorescence of SiR fluorophore by displacement of Xyl-SiR by the higher affinity guest ADA functionalized AuNPs as seen in Fig. 7d. We titrated the complex of Xyl-SiR and CB[7]-BHQ3 with 20 nM of ADA-NPs and observed fluorescence recovery of SiR, thus indicating the ability of the nanoparticle to enable functional displacement of xylene conjugated SiR fluorophore from CB[7] via host-guest interactions.

CB[7]-BHQ3 conjugation:

CB[7]-NH_2 was reacted with NHS ester of BHQ3 in the presence of triethylamine base in dry DMF for 9 h at room temperature and then purified using reversed-phase HPLC. The detailed protocol is reported in our previously published manuscript.[15]

Xyl-SiR conjugation:

Xyl-EDA-PC was reacted with NHS ester of Silicon rhodamine in the presence of triethylamine base in dry DMSO for 6 h at room temperature to get the Xyl-PC-SiR. The PC group-protected Xyl-SiR was dissolved in 1:1 water/acetonitrile mixture and irradiated with 365 nm UV light (50 mW/cm^2) for 5 min and purified using reversed-phase HPLC to get the final product Xyl-SiR. The preprint manuscript reports the detailed protocol (bioRxiv doi: https://doi.org/10.1101/2023.04.24.538015).

20.6 Determination of cell viability upon treatment with Xyl-DTX, CB[7]·Xyl-DTX, [CB[7]·Xyl-DTX + ADA-NPs], [CB[7] +ADA-NPs] and ADA-NPs

1. HeLa cells were seeded at ~10,000 cells/100 µl cell media in a 96-well plate.

2. After 24 h, the cells were washed with 1× DPBS and incubated for 24 h with

(i) Xyl-DTX (final conc.: 3 µM, 2.42 µl from the stock solution of 372.51 µM in DMSO) dissolved in 300 µl cell media containing 10 µM verapamil and added in three different wells 100 µl in each.

(ii) Xyl-DTX + CB[7] (2.42 µl from the stock solution of 372.51 µM in DMSO + 1.08 µl from the stock solution of 5 mM in milli-q water) were dissolved in 26.5 µl milli-q water and kept for complexation for 1 h *ex-situ* in a microcentrifuge tube. It is then mixed with 270 µl cell media containing 10 µM verapamil (Xyl-DTX: CB[7] = 3 µM: 18µM) and added in three different wells, 100 µl in each.

(iii) Xyl-DTX + CB[7] (2.42 µl from the stock solution of 372.51 µM in DMSO + 1.08 µl from the stock solution of 5 mM in milli-q water) were dissolved in 26.5 µl milli-q water and kept for complexation for 1 h ex-situ in a

microcentrifuge tube. It is then mixed with 270 µl cell media containing 10 µM verapamil (Xyl-DTX: CB[7] = 3 µM: 18µM) and added in three different wells, 100 µl in each. ADA-NPs (final conc. =125 nM, 1.77 µl from the stock solution of 7.05 µM in milli-q water) were added *in situ* at 0 h in these wells.

(iv) ADA-NPs (final conc. = 125 nM, 5.34 µl from the stock solution of 7.05 µM in milli-q water) dissolved in 294.66 µl cell media containing 10 µM verapamil and added in three different wells, 100 µl in each.

(v) ADA-NPs + CB[7] (5.34 µl from the stock solution of 7.05 µM in milli-q water + 1.08 µl from the stock solution of 5 mM in milli-q water) dissolved in 23.58 µl milli-q water and kept for complexation for 1 h *ex-situ* in a microcentrifuge tube. It is then mixed with 270 µl cell media containing 10 µM verapamil (ADA-NPs: CB[7] = 125 nM: 18 µM) and added in three different wells, 100 µl in each.

3. After 24 h, the cells were washed thrice and incubated with cell media.

4. After 24 h, the cells were washed once and incubated with media containing MTT solution (90 µl 1 × PBS (pH=7.4) + 10 µl MTT from stock) per well.

5. After ~2 h incubation, the MTT solution was removed and 100 µl HPLC grade DMSO was added to each well.

6. After ~15 min, absorbance was measured at 570 nm (experimental wavelength) and 690 nm (reference wavelength), and cellular viability were determined by using the formula shown below:

$$\text{Percentage of cell viability (\%)} = (\text{Absorbance}_{570\text{ nm-}690\text{ nm}}\text{ treatment}/\text{Absorbance}_{570\text{ nm-}690\text{ nm}}\text{ control}) \times 100\ \%$$

Cell viability data from this experiment is shown in Fig. 7e.

20.7 Protocol of cellular imaging of HeLa cells expressing GFP labelled α-tubulin upon treatment with Xyl-DTX, CB[7]·Xyl-DTX, [CB[7]·Xyl-DTX+ADA-NPs] and ADA-NPs:

1. HeLa cells expressing GFP-labelled α- tubulin were seeded at ~8000 cells / 150 µl media in 4-chamber glass bottom 35 mm dish with 20 mm bottom well (For the comparison with control)

2. After 24 h of plating, cells were washed with 1× DPBS and incubated with

(i) Xyl-DTX (final conc.: 3 µM, 1.21 µl from the stock solution of 372.51 µM in DMSO) with DMEM-phenol red-free media containing 10 µM verapamil in the first well.

(ii) Xyl-DTX + CB[7] (1.21 µl from the stock solution of 372.51 µM in DMSO + 0.54 µl from the stock solution of 5 mM in milli-q water) were dissolved in 28.25 µl milli-q water and kept for complexation for 1 h *ex-situ* in a

microcentrifuge tube. It is then mixed with 120 µl DMEM-phenol red-free media (Xyl-DTX: CB[7] = 3 µM: 18 µM) containing 10 µM verapamil and added to the second well.

(iii) Xyl-DTX + CB[7] (1.21 µl from the stock solution of 372.51 µM in DMSO + 0.54 µl from the stock solution of 5 mM in milli-q water) were dissolved in 28.25 µl milli-q water and kept for complexation for 1 h *ex-situ* in a microcentrifuge tube. It is then mixed with 120 µl DMEM-phenol red-free media (Xyl-DTX: CB[7] = 3 µM: 18 µM) containing 10 µM verapamil and added to the third well. ADA-NPs (final conc.: 125 nM, 2.66 µl from the stock solution of 7.05 µM in milli-q water) were added *in situ* at 0 h in the dish directly.

(iv) ADA-NPs (final conc.: 125 nM, 2.66 µl from the stock solution of 7.05 µM in milli-q water) dissolved in 147.34 µl DMEM-phenol red-free media containing 10 µM verapamil and added to the fourth well.

3. SIM images were acquired with a 488 nm excitation laser after incubation of the compound (Fig.7f and S29).

21. Concentration measurement of Xyl-DTX using UV-Visible study.

We first prepared the 150 µM, 100 µM, 50 µM, 25 µM and 12.5 µM stock solutions of docetaxel in acetonitrile (ACN). We recorded the absorbance spectra of DTX stock solutions in a 3.5 ml cuvette. Then we plotted the graph between the concentration of DTX and absorbance at 235 nm of each stock solution and did the linear fitting of it to get the slope of the line. From the slope, we have calculated the extinction coefficient of DTX at 235 nm and found it to be 14910 $M^{-1}cm^{-1}$. Then we prepared the stock of *p*-xylenediamine (Xyl) 400 µM, 200 µM,100 µM, 50 µM, 25 µM, and 12.5 µM in ACN and recorded the absorbance spectra of Xyl stock solutions in a 3.5 ml cuvette. Then we plotted the graph between the concentration of Xyl and absorbance at 235nm of each stock solution and did the linear fitting of it to get the slope of the line. From the slope, we have calculated the extinction coefficient of Xyl at 235 nm and found it to be 3000 $M^{-1}cm^{-1}$. Then we add the extinction coefficient value of DTX and Xyl observed at 235 nm and got the final value of extinction coefficient value 27910 $M^{-1}cm^{-1}$, which was further used to calculate the concentration of docetaxel conjugated xylene (Xyl-DTX) (Fig. S30).

22. Microscopy set up and image processing

Structured illumination microscopy (SIM) was carried out using an inverted Zeiss ELYRA PS1 microscope equipped with 4 excitation lasers and an sCMOS camera. Live tissue samples kept in microscope imaging dishes were placed under the microscope maintained at 37°C. The 5% CO2 atmosphere and fluorescence microscopic images were captured by structured illumination using an inverted Zeiss ELYRA PS1 microscope. One laser has been used for excitation: 488 nm (200 mW) for respective excitation of green fluorescent protein. Imaging was performed using a Zeiss oil–immersion objective (alpha Plan–apochromat DIC 63x/1.40 Oil DIC M27, numerical

aperture (NA) 1.40 oil). Fluorescence light was spectrally filtered with emission filters (MBS– 488+EF BP 495–570/LP 750 for laser line 488 and imaged using a PCO edge sCMOS camera.

Image processing and data analysis: Structured illumination image was reconstructed using a structured illumination analysis package for Zen 2.0 software (Zeiss). Additional software has been used for color adjustment (ImageJ).

23. Synthesis of Benz-Cy5

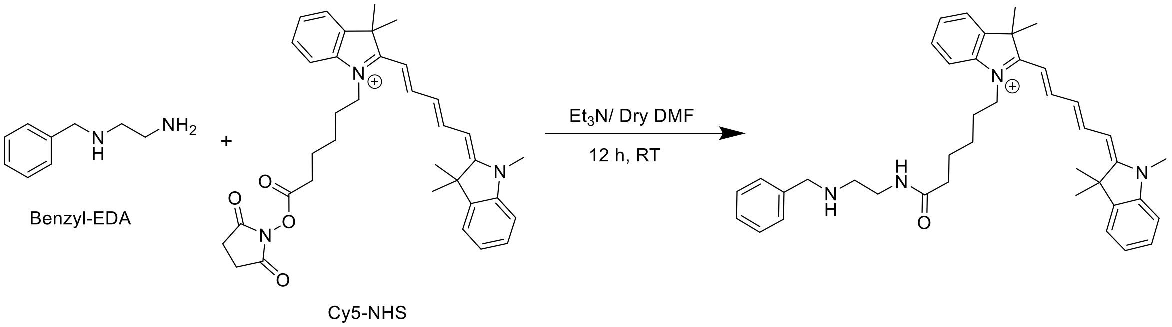

Reaction procedure:

Benzyl-EDA (178.6 µg, 7.15 µl from 25 µg/µl stock in dry DMF, 1.181 µmol) was taken in a 0.5 ml microcentrifuge tube. Triethyl amine (9.86 µl from the stock of 1% (v/v) triethylamine in dry DMF, 0.708 µmol) was added to it. Then, NHS ester of Cy5 (180 µg, 9 µl from 20 µg/µl stock in dry DMF, 0.236 µmol) was added to the reaction mixture and stirred at room temperature for 12 h. The reaction mixture was diluted using water and injected into HPLC for purification. HRMS (ESI-MS): calculated for $C_{41}H_{51}N_{4}O^{+}$: 615.4057, found 615.4063 [M^{+}] and 308.2058 [M^{+}+H^{+}] (Fig. S31). LC-Chromatogram of the purified product is shown in Fig. S32.

24. Preparation of Benz-conjugated antibody

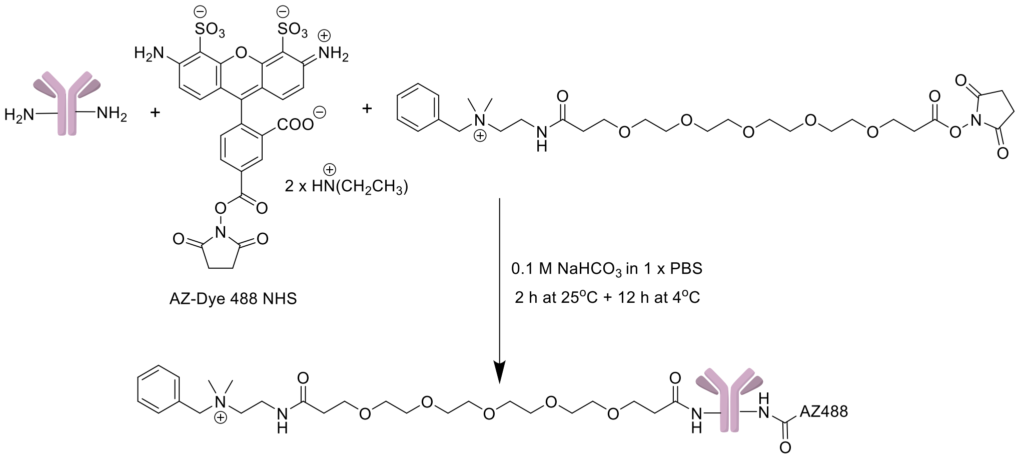

Reaction procedure:

1. Secondary antibody donkey anti-rat (13 µg, 0.086 nmol) was first taken in a 0.5 mL microcentrifuge tube.

2. Then 1.3 µl of 1 M $NaHCO_3$ in 1 × PBS (pH=7.4) was added to make it finally 0.1 M $NaHCO_3$ in the final reaction mixture.

3. Then AZDye 488-NHS (0.35 µl from a stock of 1 mg/ml in dry DMSO) and 1.02 µL of Benzyl-NMe_2-EDA-PEG_5-NHS (1.02 µl from a stock of 2 mg/ml in dry DMSO) were added to the reaction mixture.

(Note: Synthesis of Benzyl-NMe_2-EDA-PEG_5-NHS is described below)

4. The reaction mixture was kept at 25°C for 2 h and then 12 h at 4°C.

5. The Conjugated antibody was then purified by ZebaTM spin column (pre-equilibrated with 1 × PBS).

Materials for purification:

1. ZebaTM spin desalting columns, 7K MWCO, 75 µl

2. 1 × PBS (pH=7.4)

3. 1.5 mL microcentrifuge tube

Procedure for purification:

1. Remove the column bottom closure and loosen the cap.

2. Place the column in a 1.5 mL microcentrifuge tube for collection (loosen cap). Centrifuge at 1000 x g for 1 minute to remove storage solution (centrifuge was used with 4°C temperature control).

3. Place a mark on the side of the column where the compacted resin is slanted upward. Place the column in the centrifuge with the mark facing outward in all subsequent centrifugation steps.

4. Then 50 µl of 1× PBS was added on top of the resin bed. Centrifuge at 1000 x g for 1 min. Repeat this step 3 times.

5. Place the column in a new 1.5 ml microcentrifuge tube and apply 12.67 µl of antibody conjugation solution to the top of the compact resin bed.

6. Centrifuge at 1000 x g for 2 minutes at 4°C to collect the purified conjugated antibody sample.

7. Discard the desalting sample after use.

8. After purification, measure the antibody conjugate concentration and the volume of the solution. Concentration: 0.849 mg/ml (11.60 µl)

Scheme: Synthesis of Benzyl-NMe$_2$-EDA.

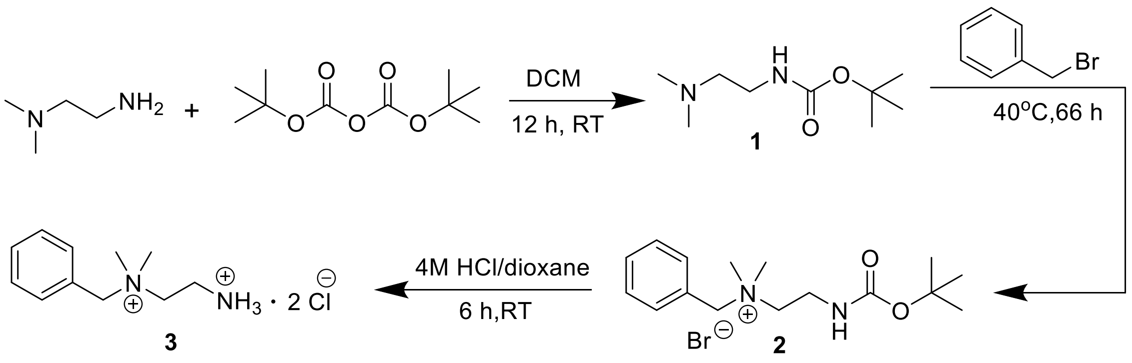

Step 1: Synthesis of tert-butyl 2-(dimethylamino)ethyl carbamate

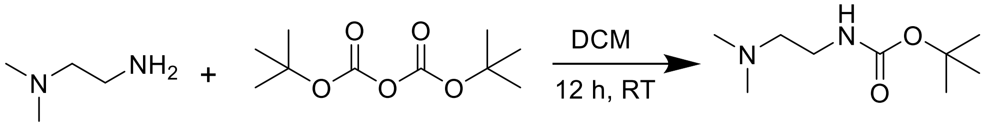

N$_1$,N$_1$-dimethylethylenediamine (2 g, 22.68 mmol) was dissolved in 15 ml of dichloromethane (DCM) in a 100 ml round bottom flask. Di-tert-butyl decarbonate (5.67 g, 27.216 mmol) was dissolved in 5 ml of DCM and added to above reaction mixture dropwise using dropping funnel up to 1 h at ice bath. The reaction was stirred at room temperature for 12 h. The reaction mixture was then washed with water (1 × 50 mL) and brine (2 × 50 mL), and the organic phase dried over anhydrous Na_2SO_4. The organic phase was dried under reduced pressure to get the tert-butyl 2-(dimethylamino)ethyl carbamate as a colorless. (3.29 g, Y.: 77%) having ^{1}H NMR (400 MHz, CDCl3): δ 5.02 (br, 1H), 3.18-3.20 (q, 2H), 2.37 (t, 2H), 2.21 (s, 6H), 1.43 (s, 9H) (Fig. S33)

Step 2: Synthesis of Benzyl-NMe$_2$-EDA-Boc.

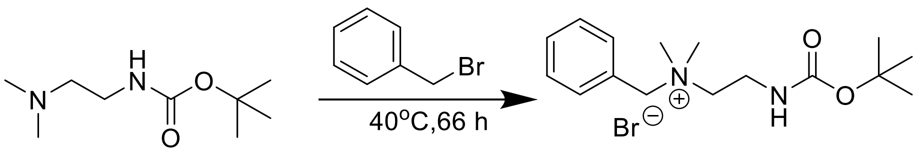

tert-butyl 2-(dimethylamino) ethyl carbamate (2 g, 10.62 mmol) was mixed with benzyl bromide (1.88 g, 11.68 mmol) in a sealed tube. The reaction mixture was heated at 40°C for 66 h. Then 40 ml toluene was added to the

reaction mixture and broken the gel manually with a spatula. Again, the reaction mixture was heated at 70°C for 3 h. The solid was filtered and washed three times with toluene and three times with diethyl ether. The compound was dried using a vacuum to get the final product as a white solid. (2.07 g, Y.: 70%) having ^{1}H-NMR (400 MHz, $CDCl_3$): δ7.64-7.45 (m, 5H), 4.93 (S, 2H), 3.82 (S, 3H), 3.30 (S, 6H), 1.79 (S, 3H), 1.41 (S, 9H). (Fig. S34)

HRMS (ESI-MS): [M^+] Calculated for $C_{16}H_{27}N_2O_2^+$: 279.2067, found 279.2064 [M^+] (Fig. S35)

Step 3: Synthesis of Benzyl-NMe_2-EDA.

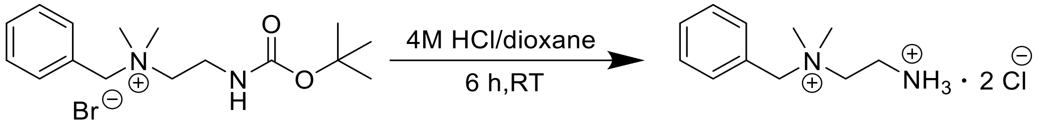

Compound 2 (250 mg) was taken in a 10 ml pear-shaped round bottom flask and mixed with 1 ml of 4M HCl/dioxane solution. The reaction mixture was stirred at room temperature for 6 h. The acid was evaporated at a high vacuum. The residue was washed three times with hexane and three times with diethyl ether. The compound was dried using a vacuum to get the final product as a white solid. (113 mg, Y.: 70%). ^{1}H-NMR (400 MHz, CD_3OD) δ:7.64-7.56 (m, 5H), 4.71 (s, 2H), 3.60 (m, 2H), 3.69 (m, 2H), 3.31 (s, 6H) (Fig. S36)

HRMS (ESI-MS): [M^+] Calculated for $C_{11}H_{19}N_2^+$: 179.1543, found 179.1540 [M^+] (Fig. S37)

Synthesis of Benzyl-NMe₂-EDA-PEG₅-NHS:

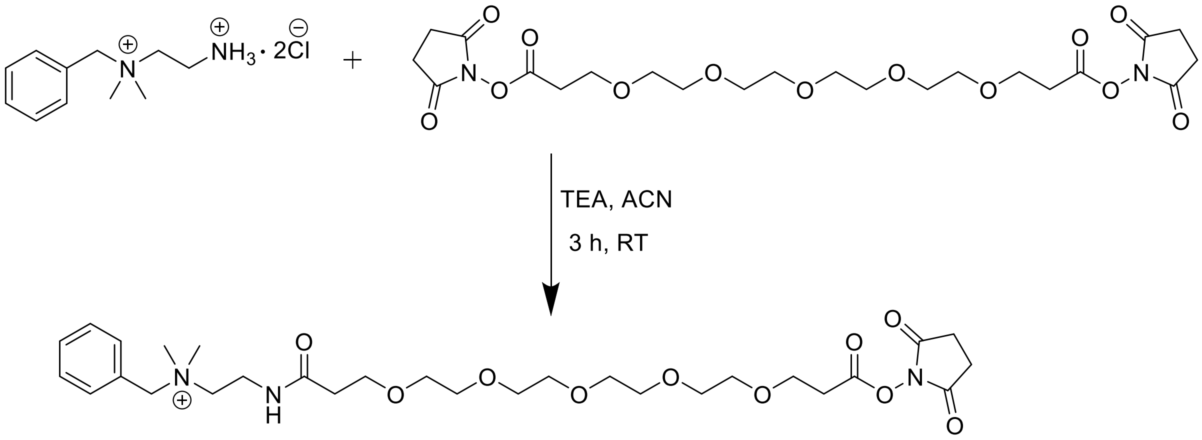

Benzyl-NMe_2-EDA (3 mg, 0.013 mmol) was dissolved in 100 µl of acetonitrile in a 0.5 ml microcentrifuge tube. Then TEA (3.87 µl, 0.026 mmol) was added to the mixture. The reaction mixture was stirred for 15 min at room temperature, and then Bis-PEG_5-NHS (70 mg, 0.13 mmol) was added to the reaction mixture and kept for stirring

at room temperature. After 3 h the product was purified through semi-preparative HPLC. Solvents used: water + 0.1% trifluoroacetic acid (A), acetonitrile + 0.1% trifluoroacetic acid (B). The mobile phase gradient used: 5 to 30% of B in 20 min then 30 to 100 % of B in 20 to 40 min. The desired product Benzyl-NMe$_2$-EDA-PEG$_5$-NHS was eluted at R_t= 20.7-22.1 min. (5.6 mg, Y.: 66%) HRMS (ESI-MS): calculated for $C_{29}H_{46}N_3O_{10}^+$ 596.3178 $[M]^+$; found 596.3175 $[M^+]$ (Fig. S38).

25. Figures

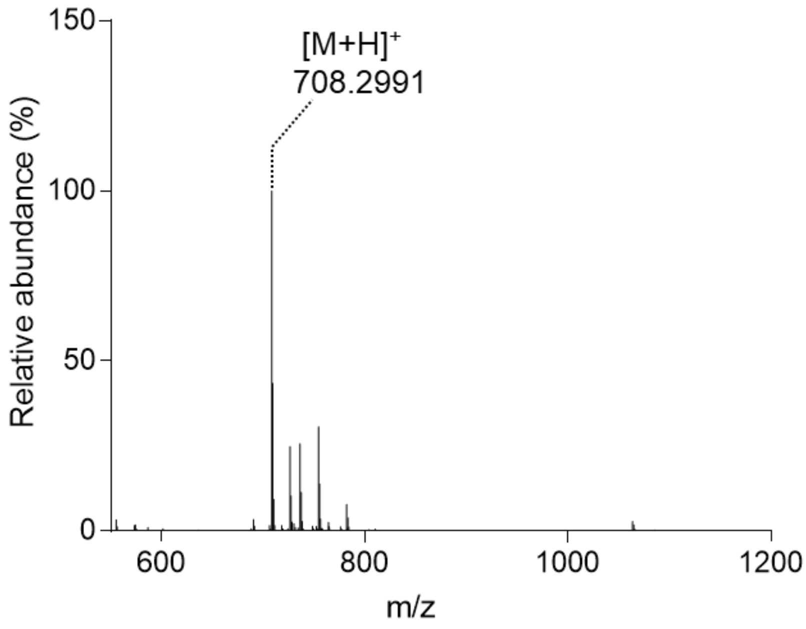

Figure S1: HRMS spectrum of DTX–NH$_2$.

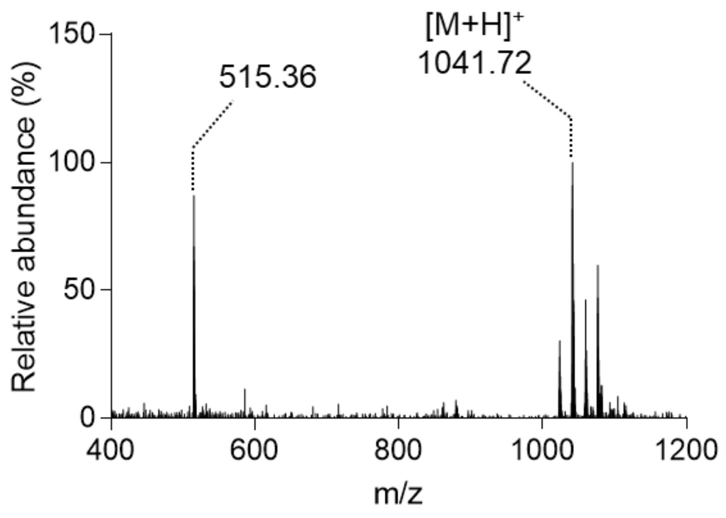

Figure S2: LCMS spectrum of DTX–BS3.

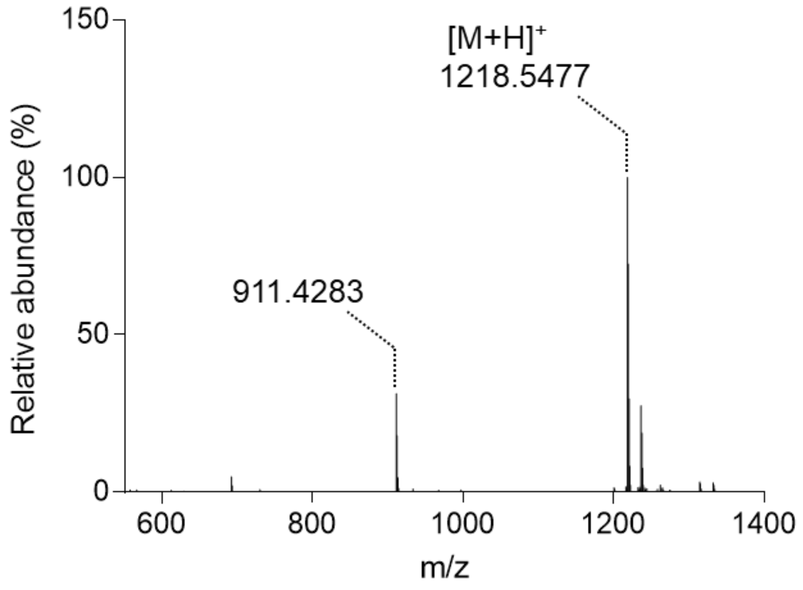

Figure S3: HRMS spectrum of Xyl-DTX-PC.

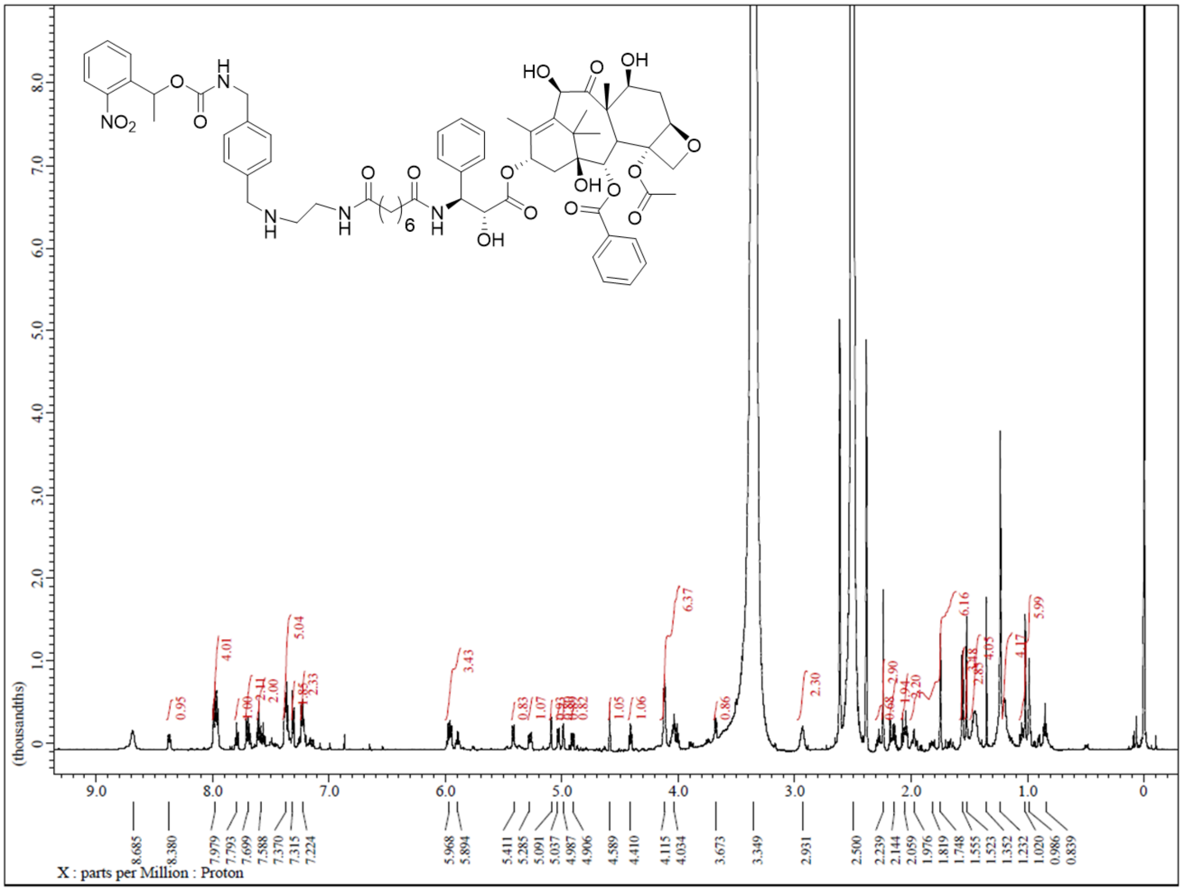

Figure S4: ^{1}H-NMR spectrum of Xyl-DTX-PC (600 MHz, DMSO–d_6).

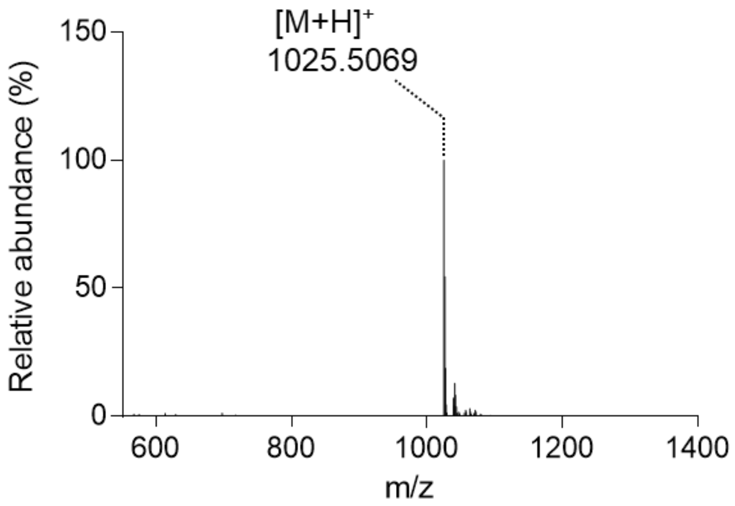

Figure S5: HRMS spectrum of Xyl-DTX.

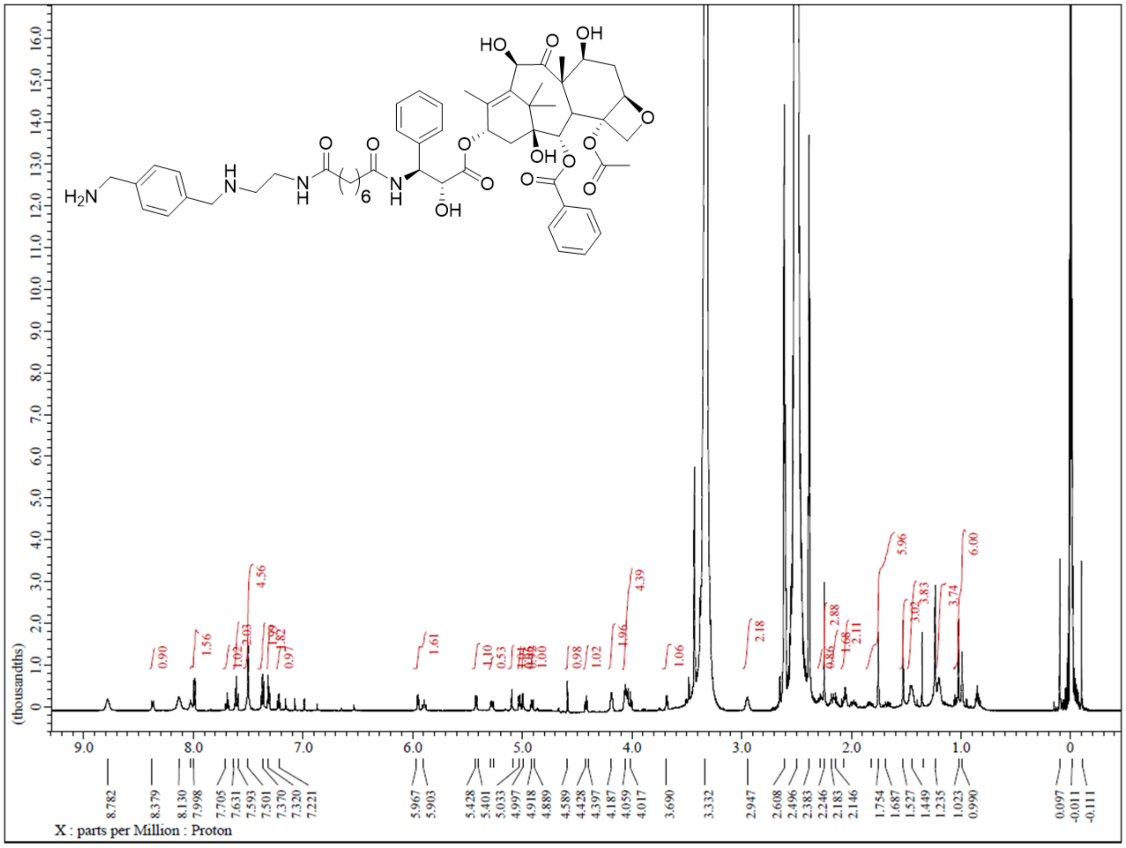

Figure S6: ^{1}H-NMR spectrum of Xyl-DTX (600 MHz, DMSO–d_6).

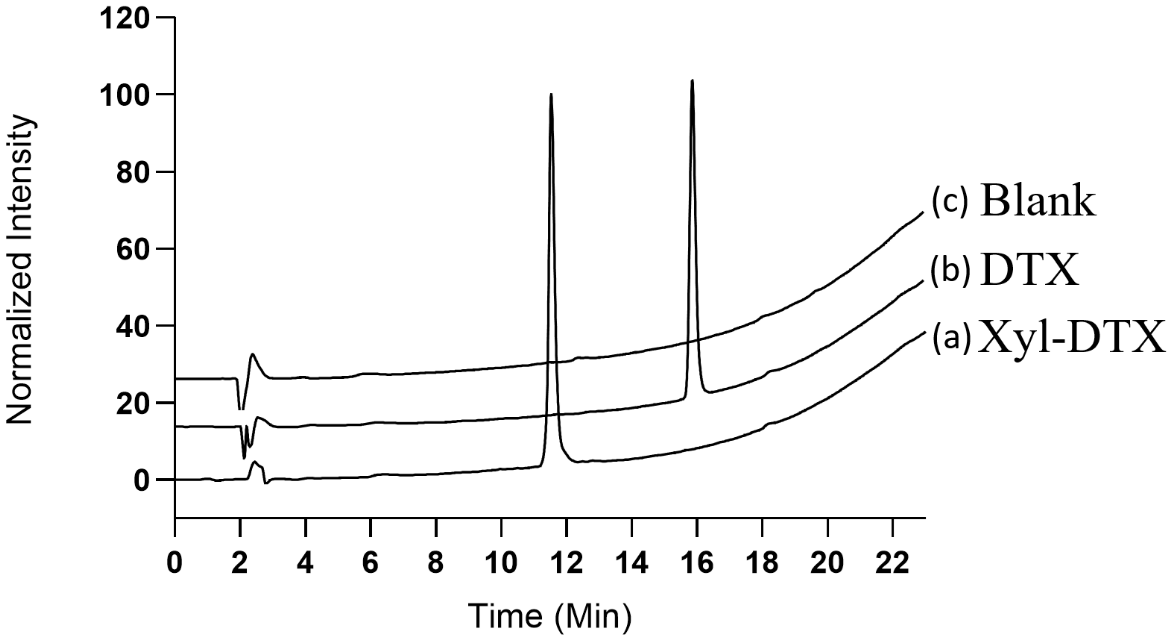

Figure S7: HPLC chromatogram of (a) Xyl conjugated docetaxel (Xyl-DTX), (b) docetaxel (DTX), and (c) Blank run. Water and acetonitrile with 0.1% TFA were used as a mobile phase. The polarity of acetonitrile varied from 5% to 100% over the time of 23 min. The conjugated product Xyl-DTX was eluted at 11.4-11.8 min and DTX at 15.8-16.1 min. The Xyl-DTX and DTX samples were prepared in 3:2 acetonitrile and water mixture for HPLC injection and recorded the HPLC chromatogram at 235 nm wavelength absorption.

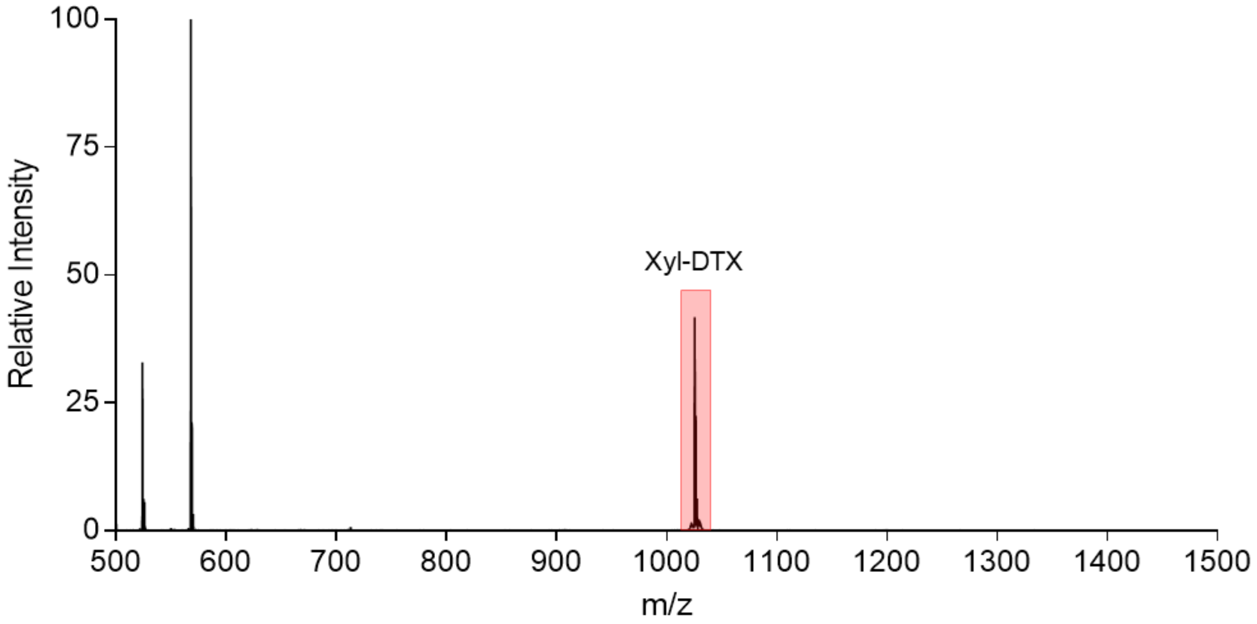

Figure S8: MALDI-MS spectrum of Xyl-DTX. [Xyl-DTX+H^+] calculated:1025.51, found 1025.51.

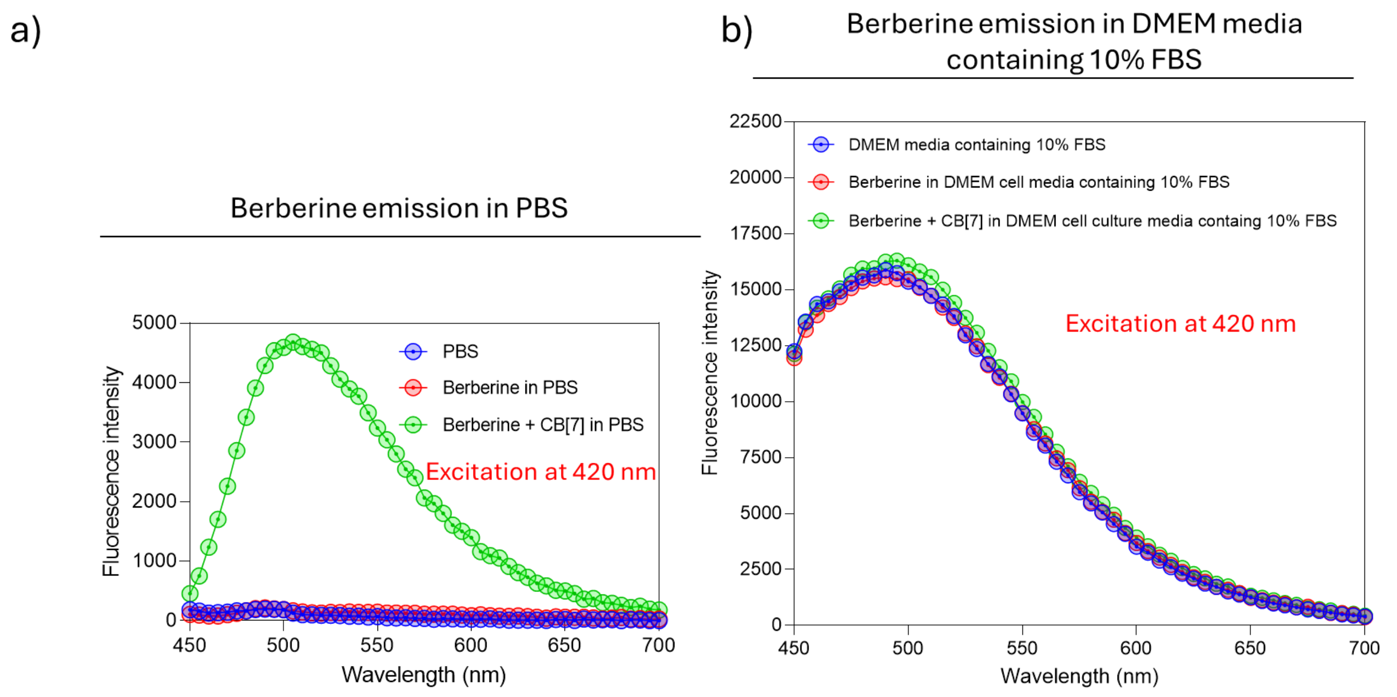

Figure S9. *In vitro* fluorescence studies depicting the significant increase in (a) Berberine (5 µM) fluorescence with the addition of CB[7] (25 µM) in 1× PBS and (b) negligible change after the addition of CB[7] (25 µM) in Berberine (5 µM) solution in DMEM-phenol red-free media with 10% FBS.

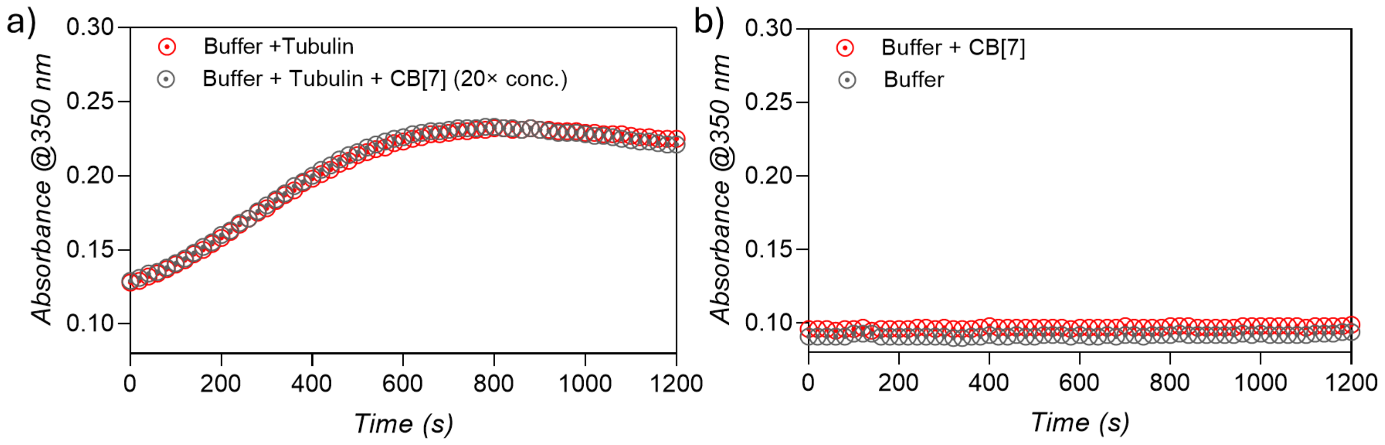

Figure S10. CB[7] controls in turbidity assay. (a) Influence of CB[7] on the turbidity during tubulin polymerization in 1× BRB80 buffer. The turbidity is monitored over time to assess the polymerization process. (b) Kinetic graph showing the turbidity of a 200 µM solution of CB[7] in the assay buffer against the buffer itself.

Experiment in DMEM cell culture media containing 10% FBS

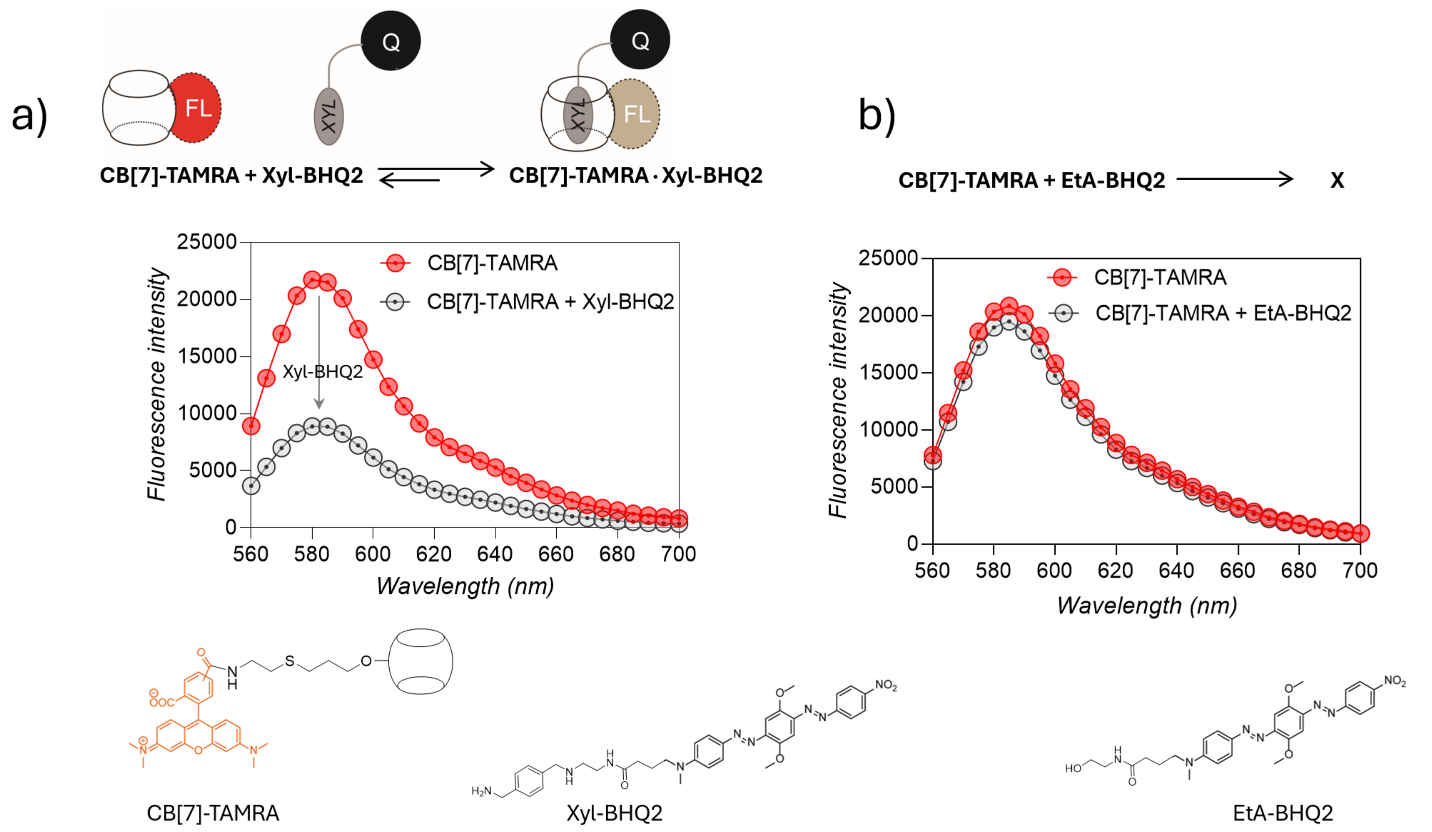

Figure S11: FRET-assays to study the binding of CB[7] with Xyl-DTX in cell culture media. Fluorescence emission (λ_{ex}=530 nm) measurement depicting (a) quenching of CB[7]-TAMRA (1 µM) after the addition of Xyl-BHQ2 (1 µM) and (b) negligible change in CB[7]-TAMRA (1 µM) fluorescence after the addition of EtA-BHQ2 (1 µM) in DMEM-phenol red-free media with 10% FBS.

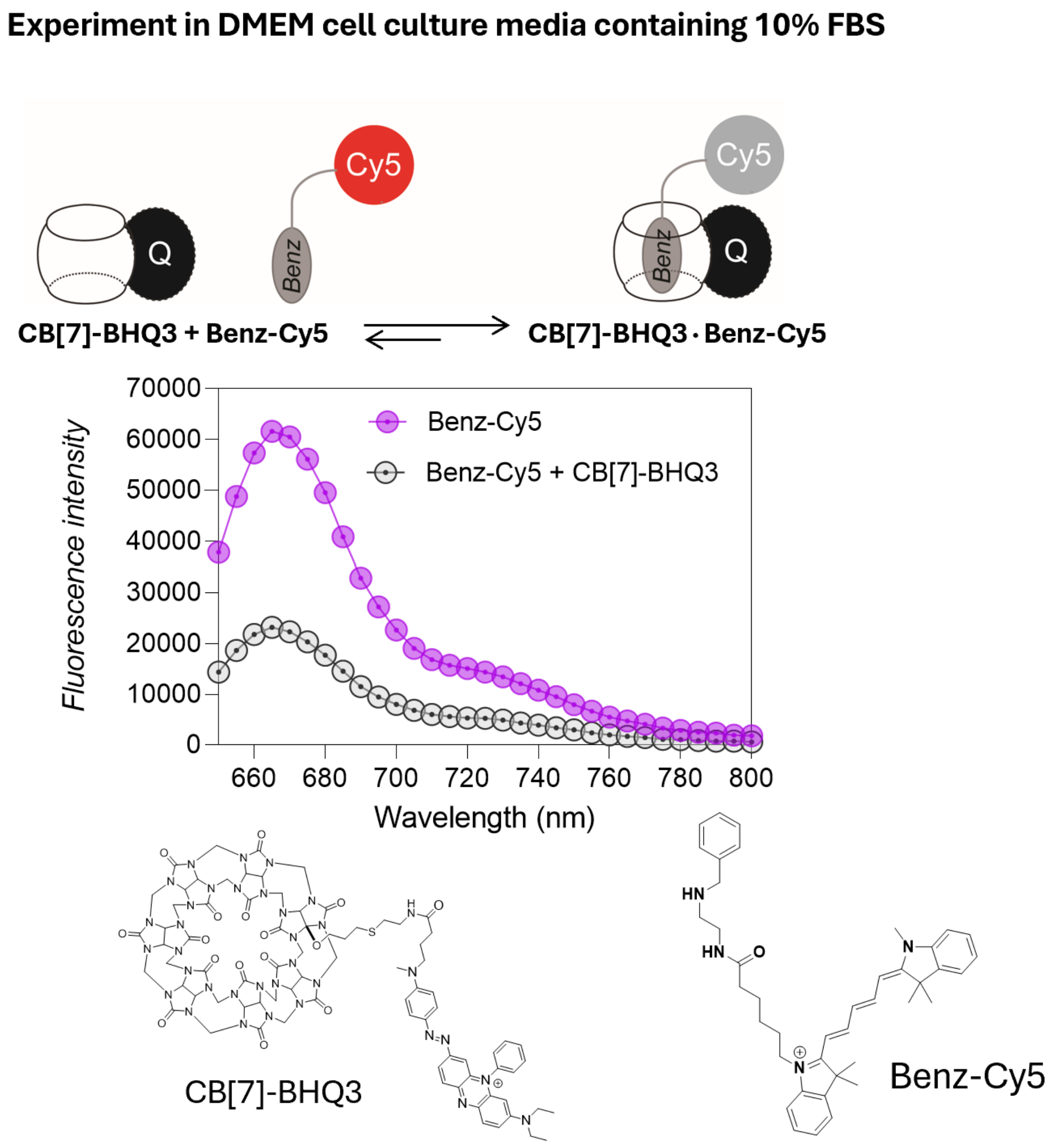

Figure S12: Fluorescence measurement (λ_{ex}=620 nm) depicting quenching of Benz-Cy5 (1 µM) fluorescence after the addition of CB[7]-BHQ3 (1 µM) in DMEM-phenol red-free media with 10% FBS.

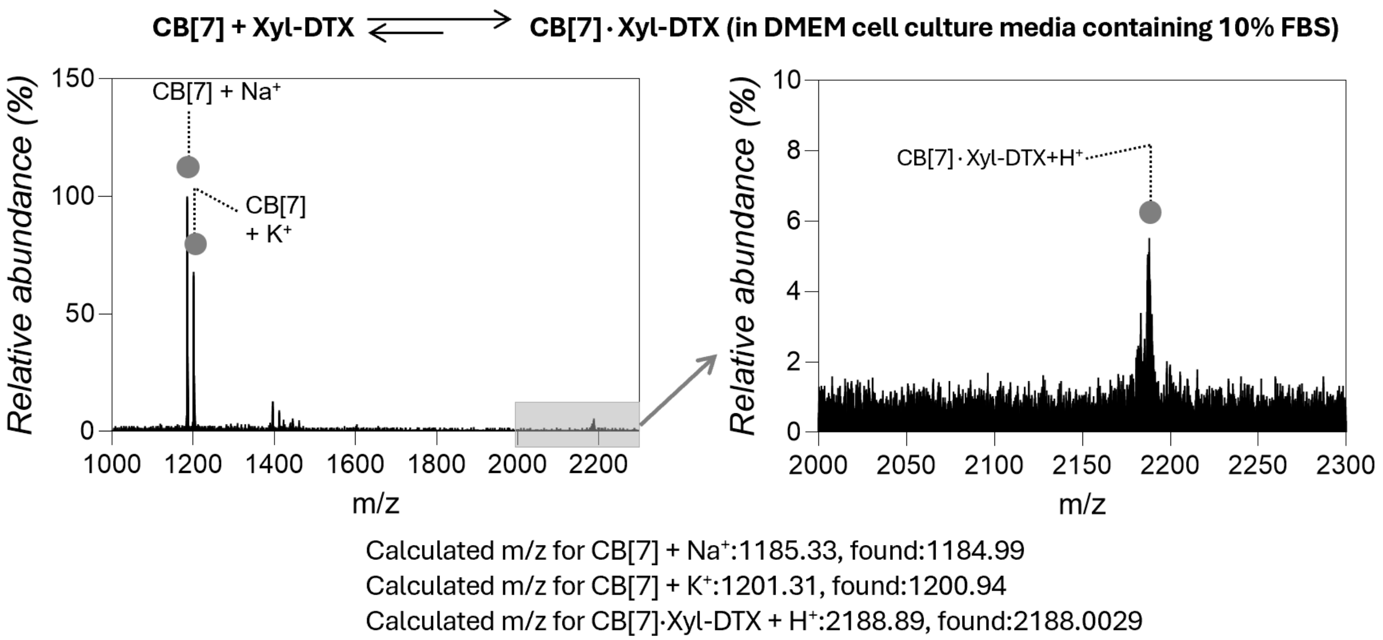

Figure S13: MALDI-MS analysis of a sample containing a mixture of CB[7] and Xyl-DTX mixture in cell culture media. MALDI-MS spectrum in positive mode was acquired after incubation of Xyl-DTX (3 µM) with CB[7] (60 µM) in Phenol-red free DMEM media with 10% FBS.

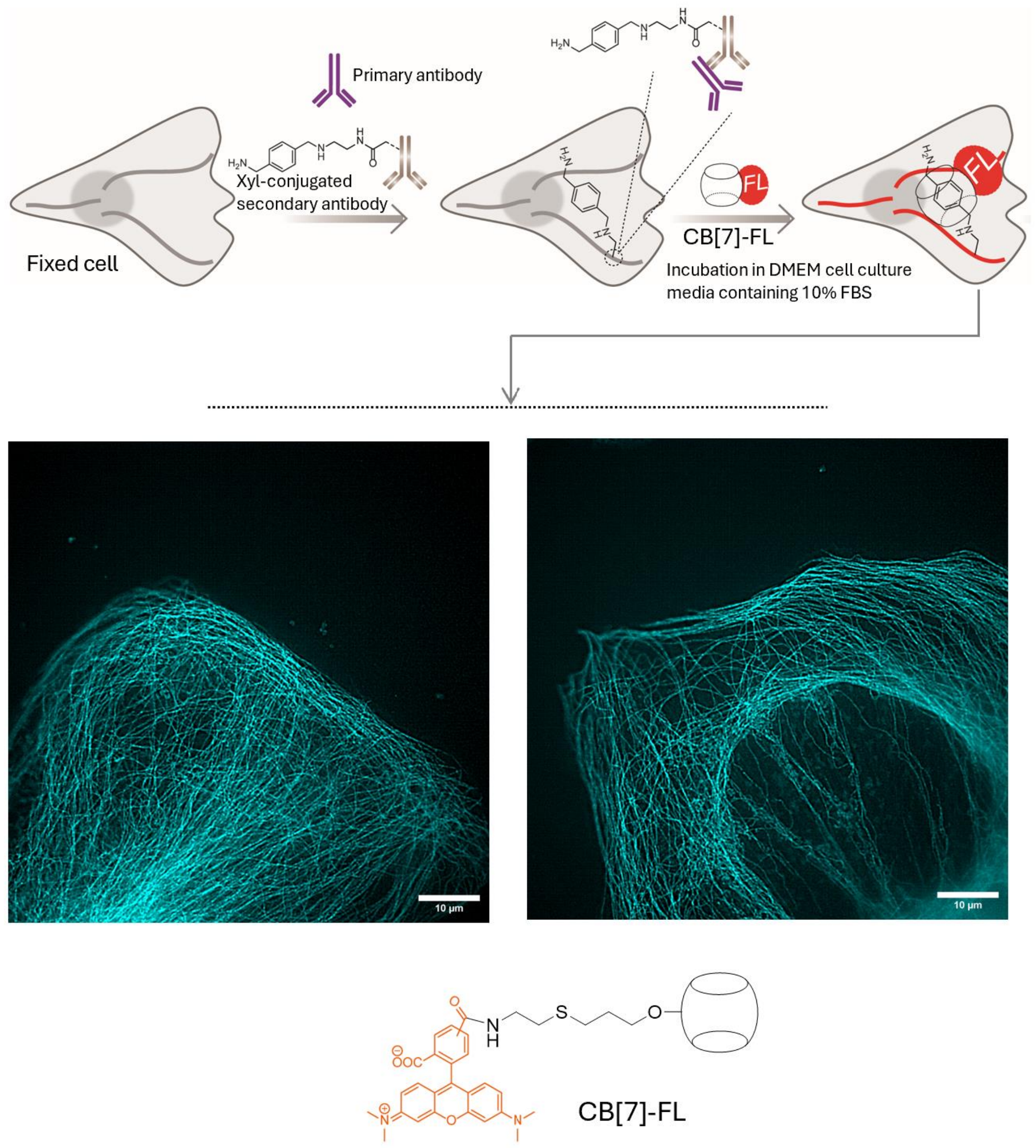

Figure S14: Cellular imaging study of microtubule labeling in BS-C1 cells after adding the CB[7]-TAMRA to the Xyl-conjugated antibody in phenol-red free DMEM media with 10% FBS. Scale bar: 10 µm

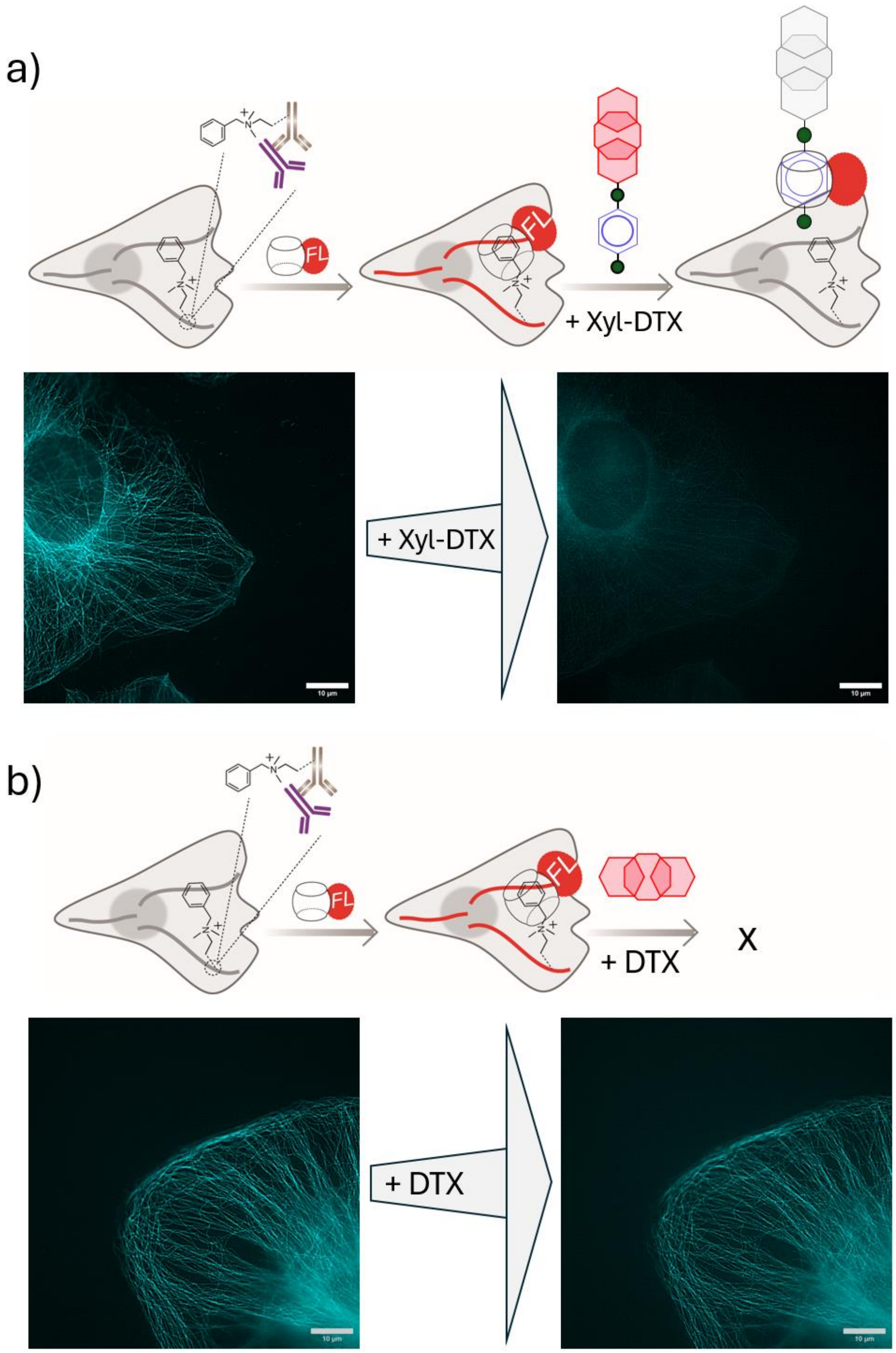

Figure S15: Cellular imaging study in BS-C1 cells shows (a) the loss of microtubule fluorescence signals after adding Xyl-DTX to a CB[7]-FL-Benz-Ab complex labeled cells, and (b) no loss of microtubule signals after adding DTX. Experiments were performed in phenol-red free DMEM media with 10% FBS. CB[7]-FL=CB[7]-TAMRA. Scale bar: 10 µm

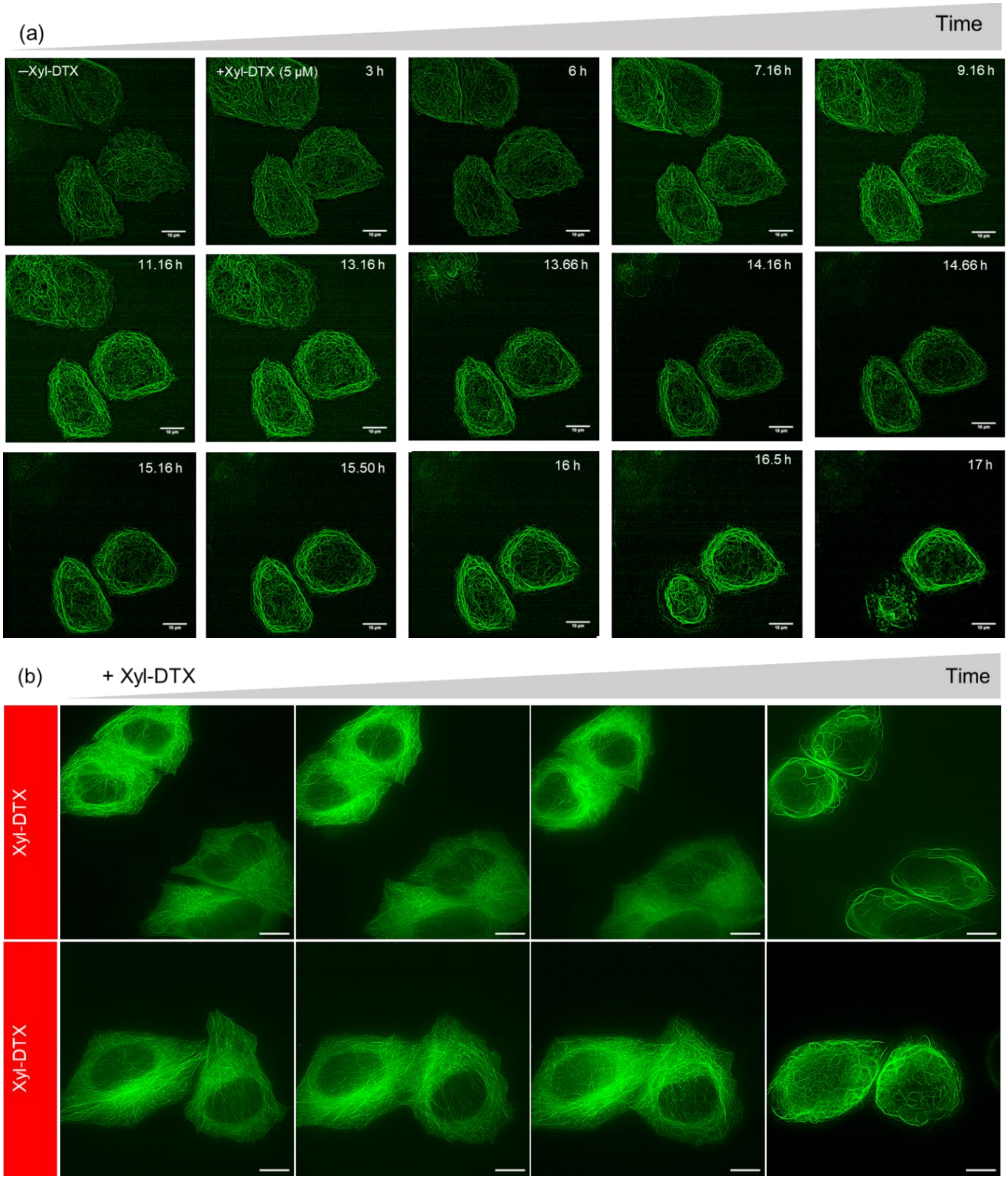

Figure S16: Time-lapse images of HeLa cells expressing GFP labelled *α-tubulin* (a) before treatment and after treatment of Xyl-DTX (times points are written on images), (b) after treatment with Xyl-DTX at different time points 0 h, 3 h, 6 h, 17 h (left to right). Scale bar: 10 μm

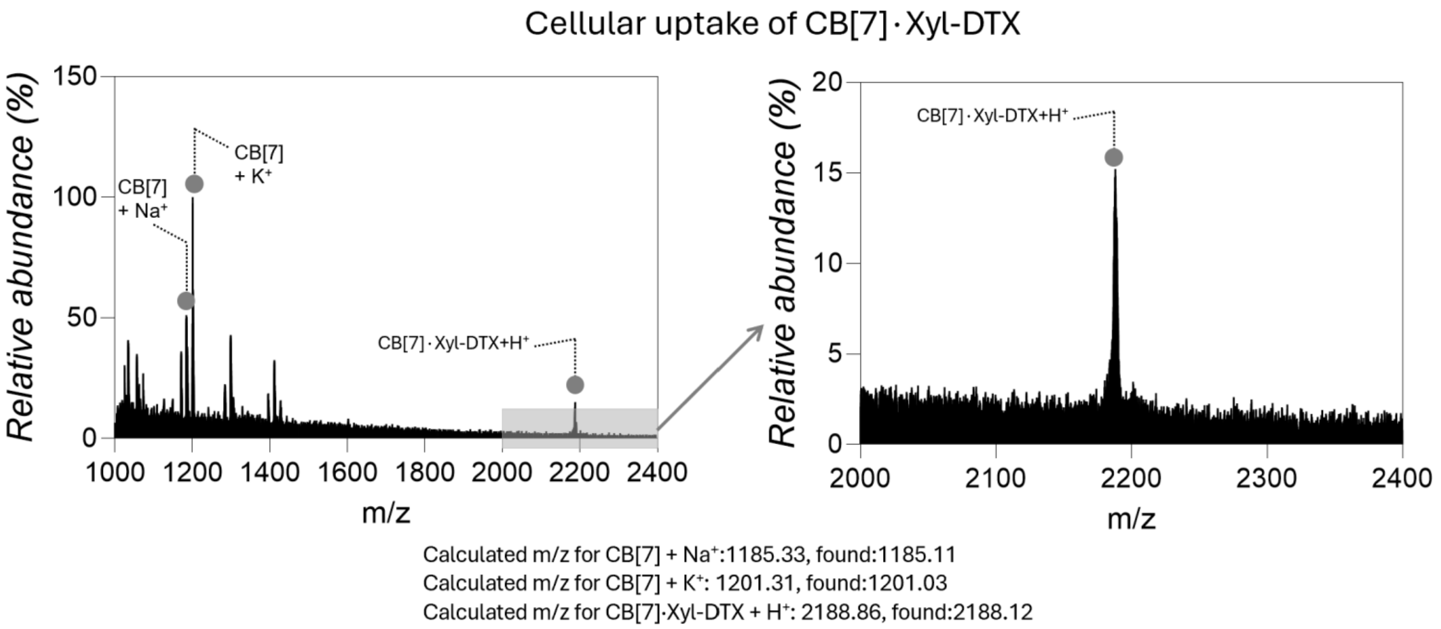

Figure S17: MALDI-MS characterization of Cellular uptake of CB[7]·Xyl-DTX complex. MALDI-MS data was acquired from the methanol-extracted fraction from cells, which was treated with CB[7]·Xyl-DTX .

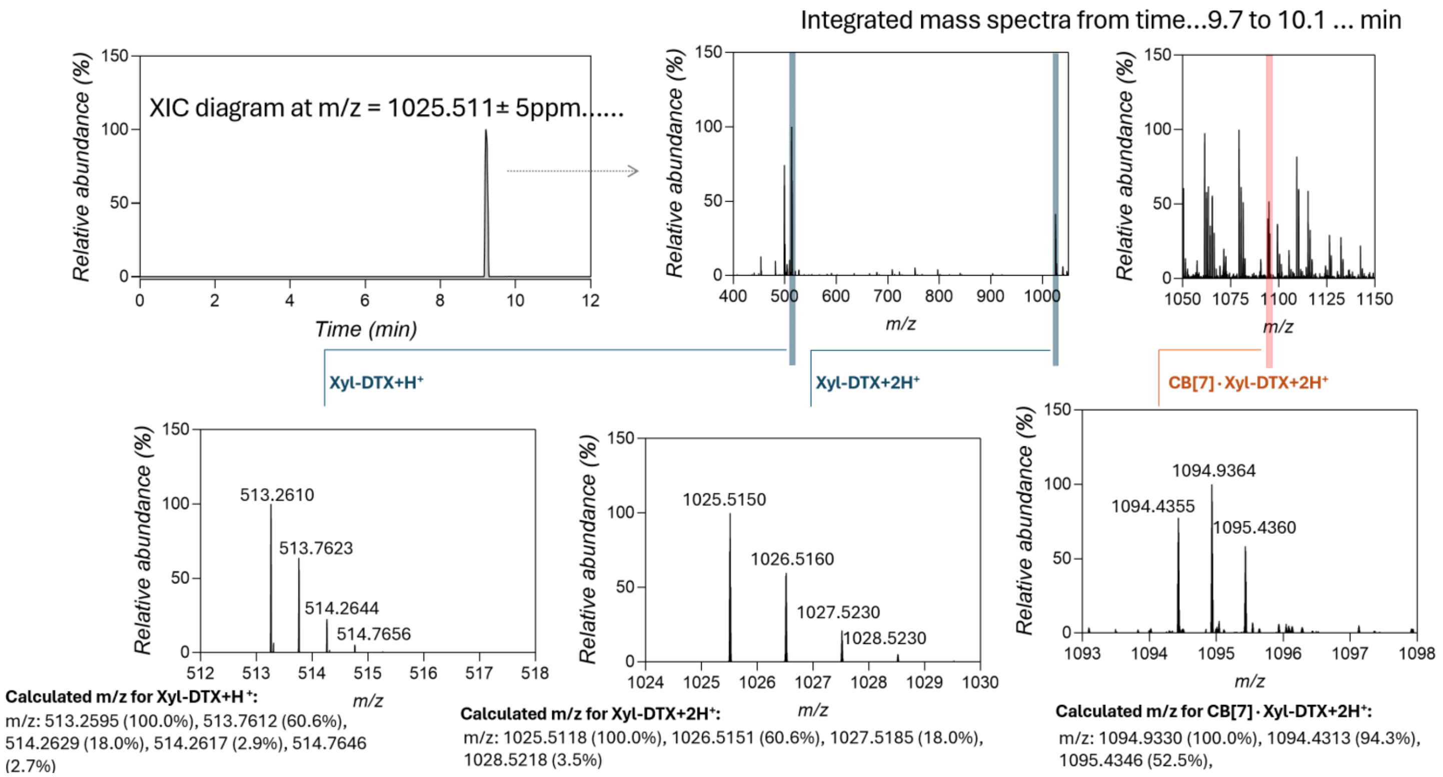

Figure S18: LC-MS characterization of Cellular uptake of CB[7]·Xyl-DTX complex. LC-MS data was acquired from the methanol-extracted fraction from cells, which was treated with CB[7]·Xyl-DTX.

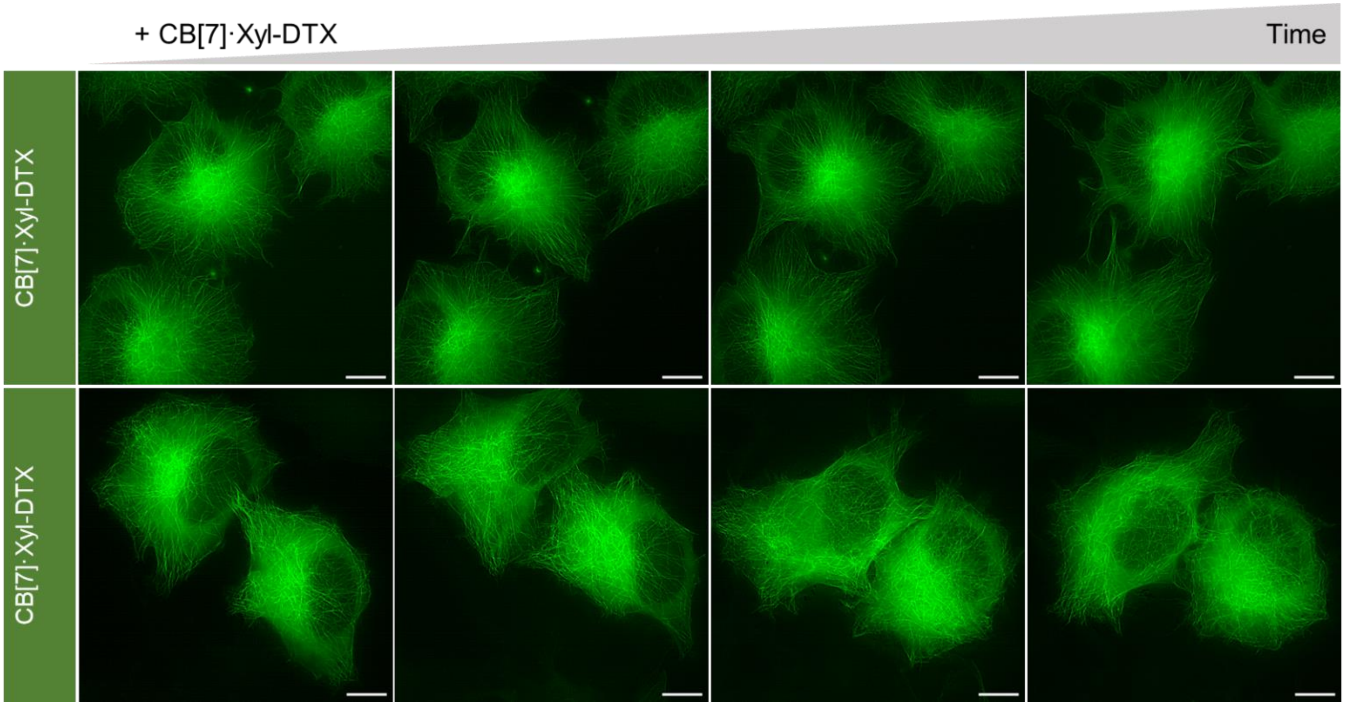

Figure S19: Time-lapse images of HeLa cells expressing GFP labelled *α-tubulin* after treatment with CB[7]·Xyl-DTX at different time points 10.5 h, 12.5 h, 20.5 h, 22.5 h (left to right). Scale bar: 10 µm

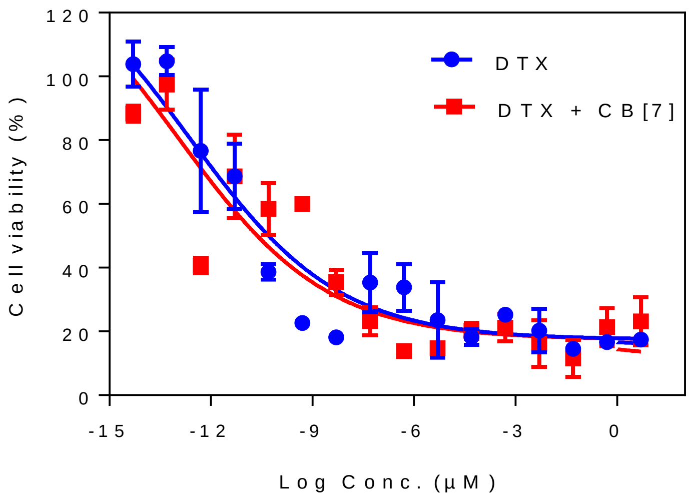

Figure S20: Cytotoxicity curve to compare the toxicity of DTX and DTX + CB[7].

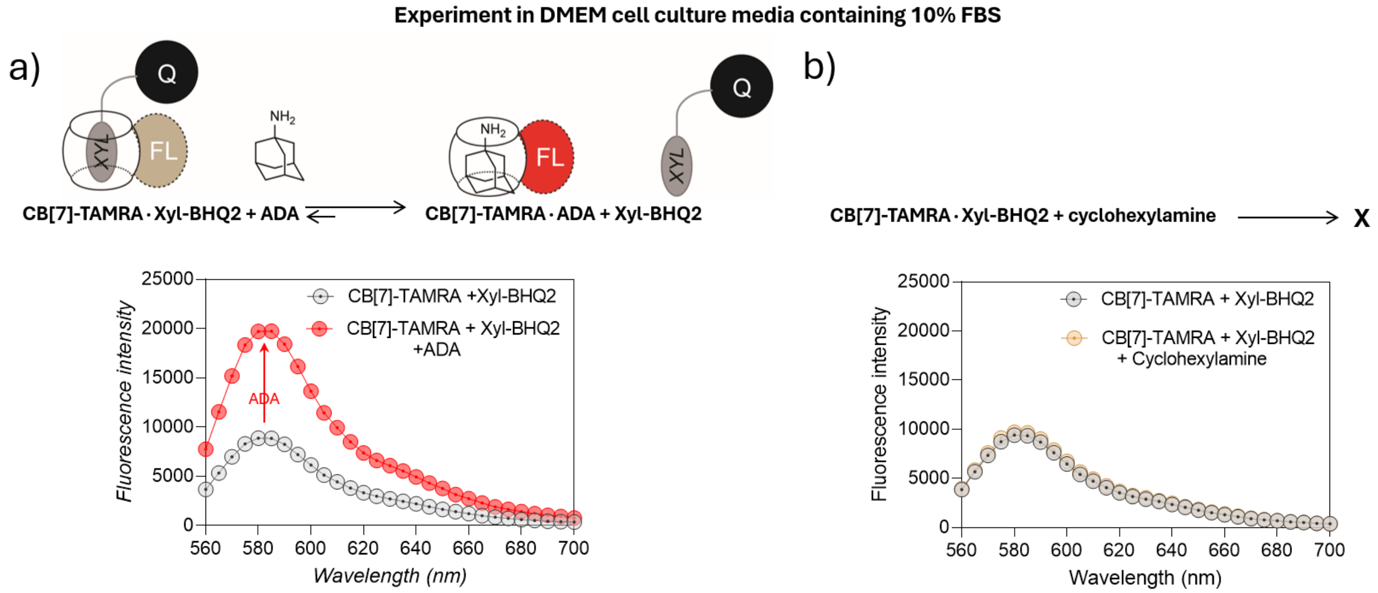

Figure S21. Fluorescence displacement assay showing (a) the recovery of fluorescence from (1:1) complex of CB[7]-TAMRA (1 µM) and Xyl-BHQ2 (1 µM) with the addition of ADA·HCl (10 µM) and (b) no recovery with the addition of Cyclohexylamine·HCl (10 µM) in phenol-red free DMEM media with 10 % FBS.

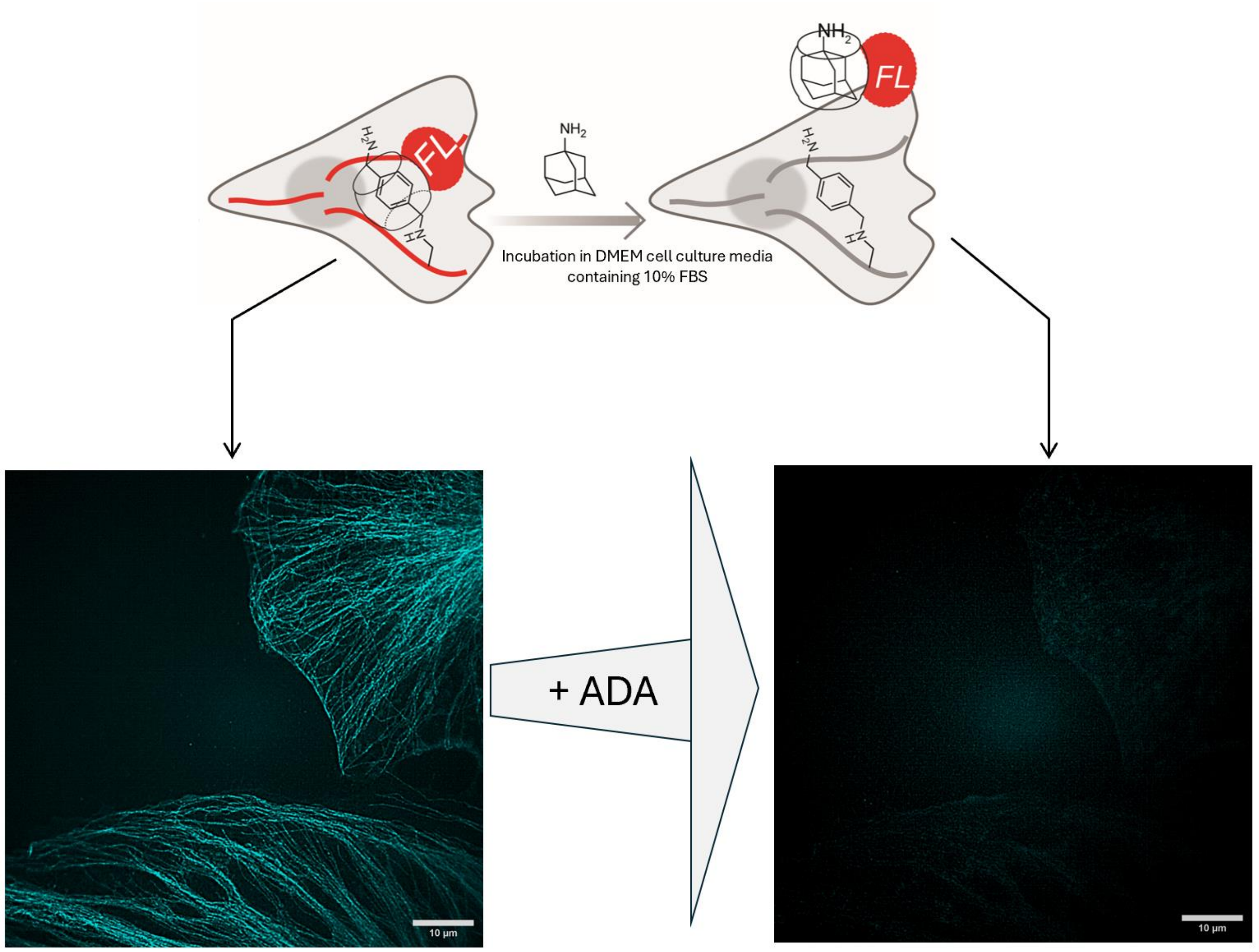

Figure S22: Cellular imaging study in BS-C1 cells shows loss of microtubule-specific fluorescence signals after adding high-affinity guest (ADA), leading to decomplexation of Xyl and CB[7]-TAMRA complex within simulated intracellular environment. Scale bar: 10 µm

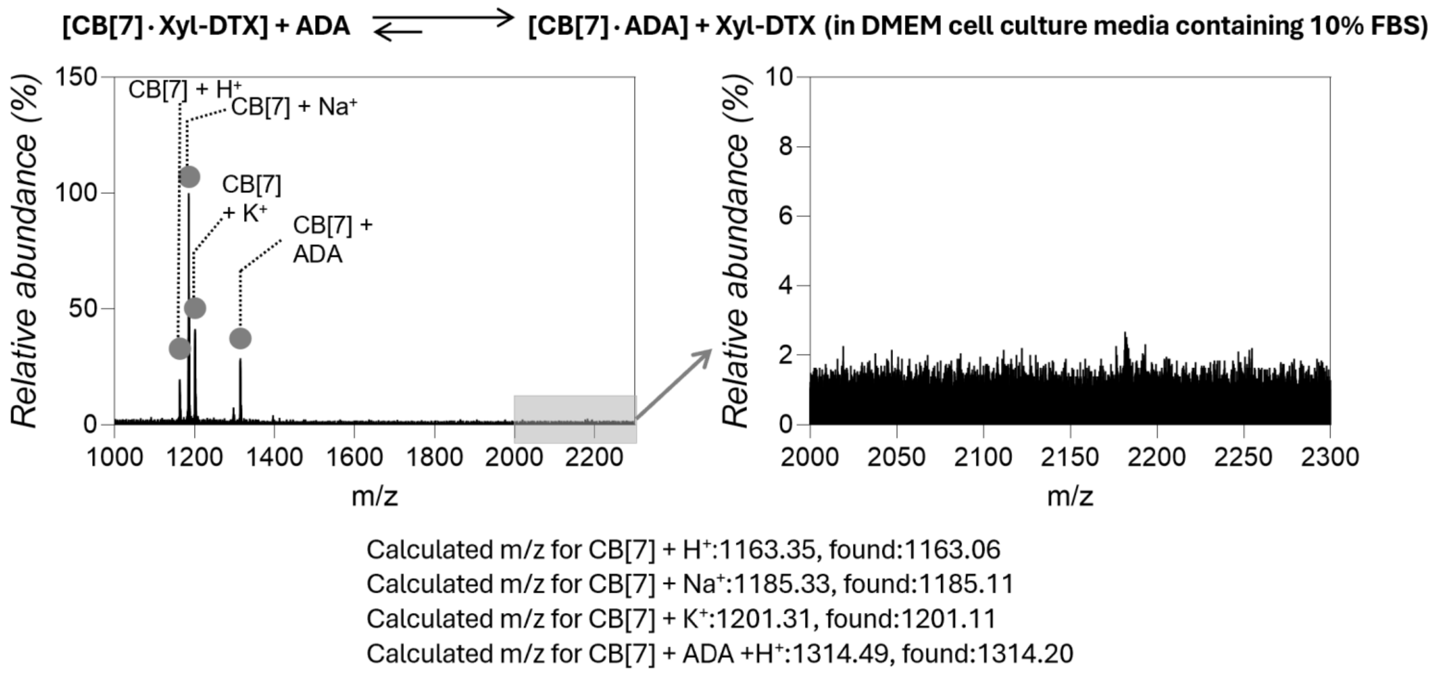

Figure S23: Displacement studies of **Xyl-DTX** and CB[7] complex after the addition of ADA. Positive MALDI-MS spectrum of ADA (60 µM) added to CB[7]·Xyl-DTX (60 µM + 3 µM) complex solution in Phenol-red free DMEM media with 10 % FBS.

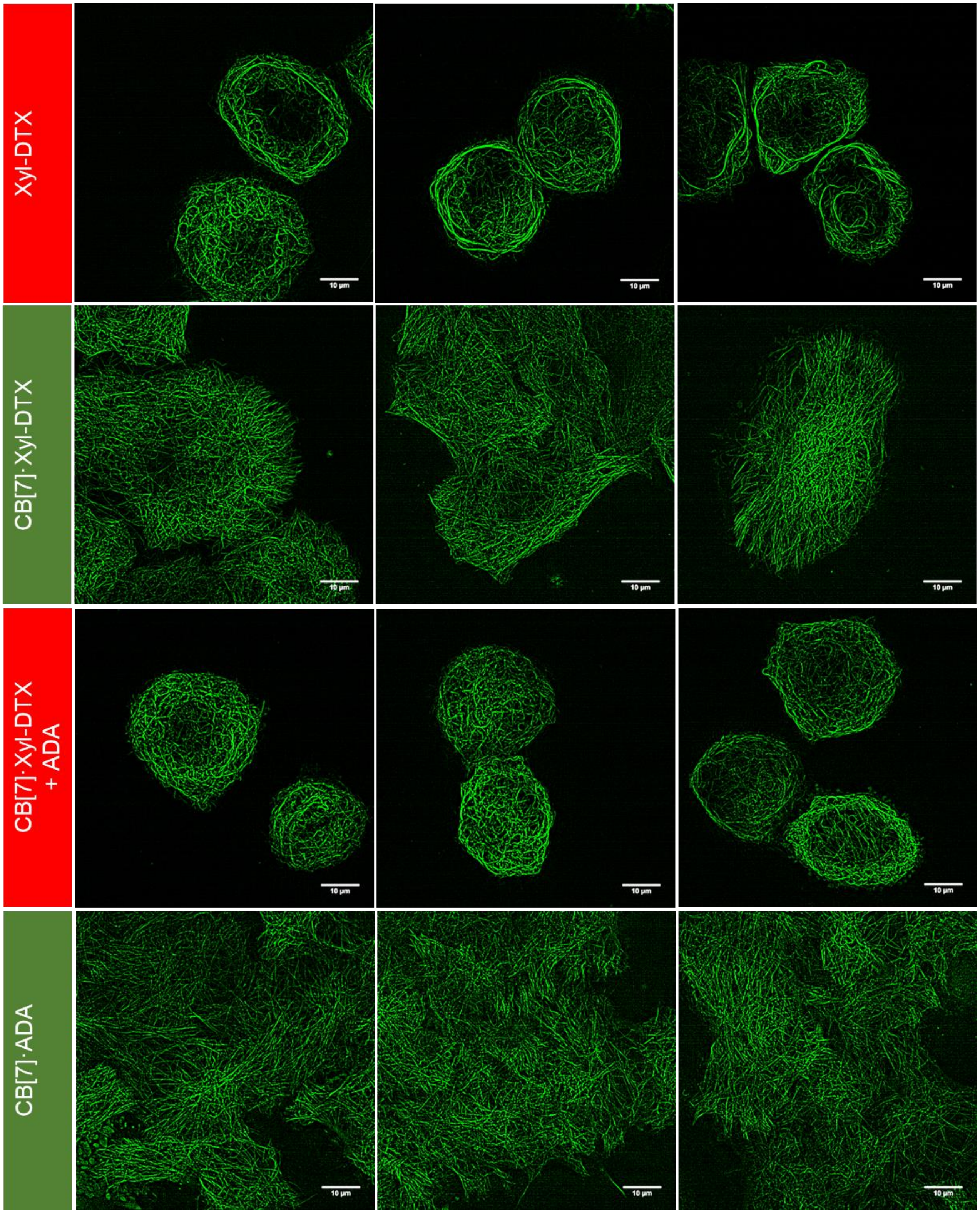

Figure S24: Cellular images of HeLa cells expressing GFP labelled *α-tubulin* treated with Xyl-DTX, CB[7]·Xyl-DTX, [CB[7]·Xyl-DTX + ADA] and CB[7]·ADA (top to down) after 23 h. Scale bar: 10 µm

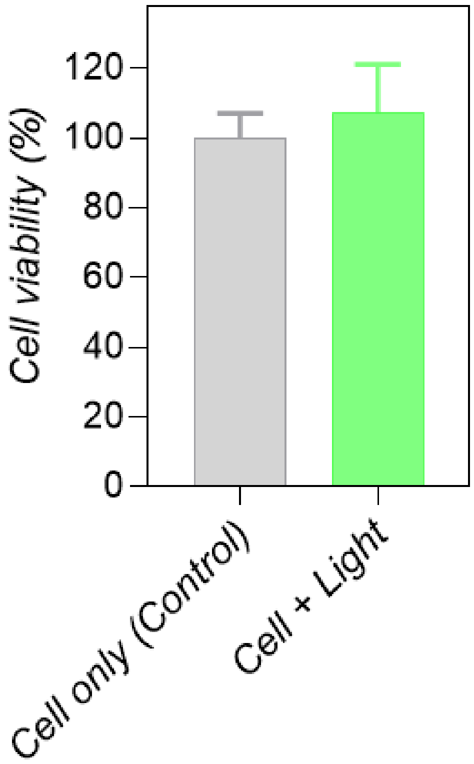

Figure S25: MTT assay showing effect of UV light irradiation on cell viability.

.

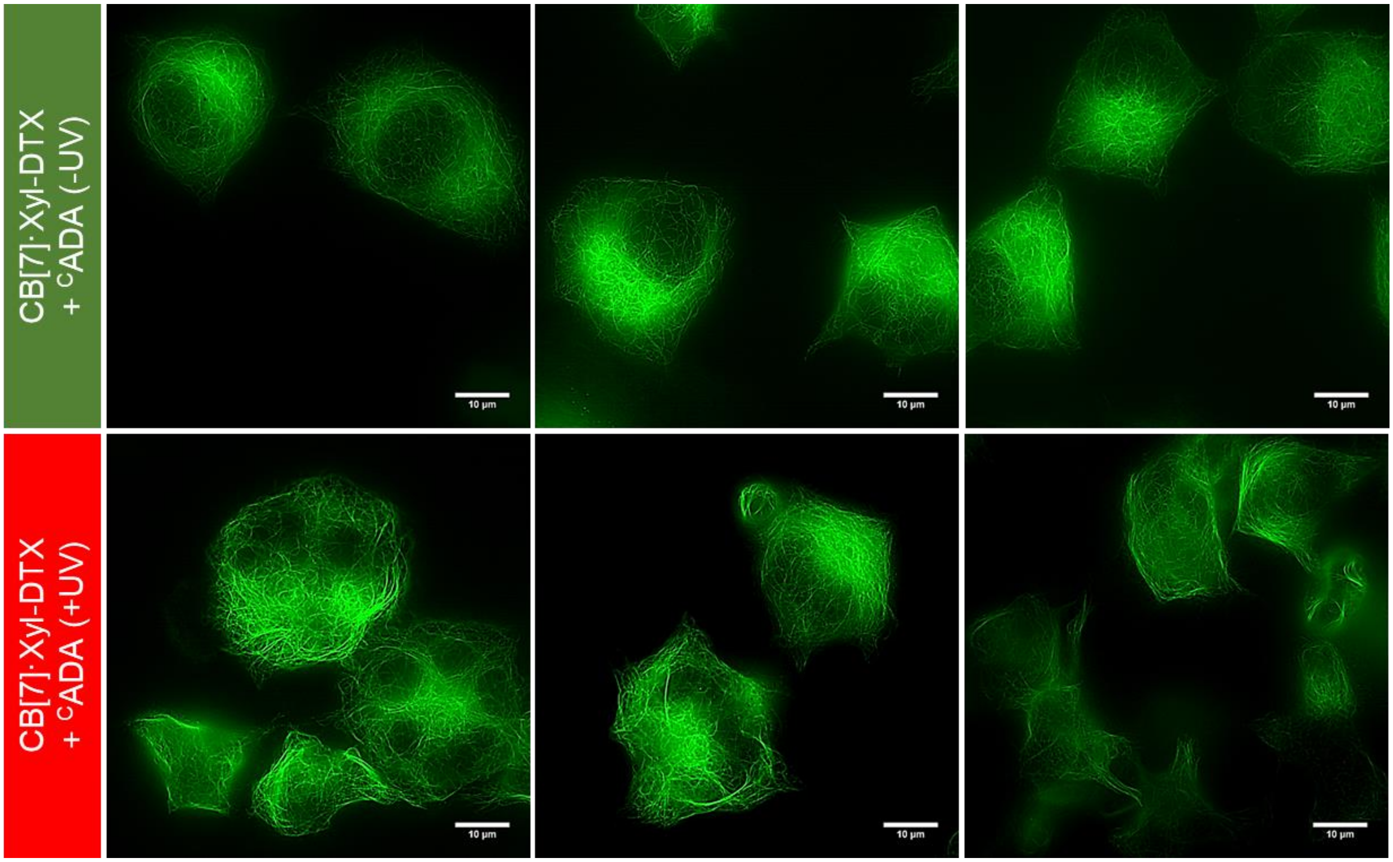

Figure S26: Cellular images of HeLa cells expressing GFP labelled *α-tubulin* treated with [CB[7]·Xyl-DTX + ^{C}ADA] without UV and with UV (top to down) after 23 h. Scale bar: 10 µm.

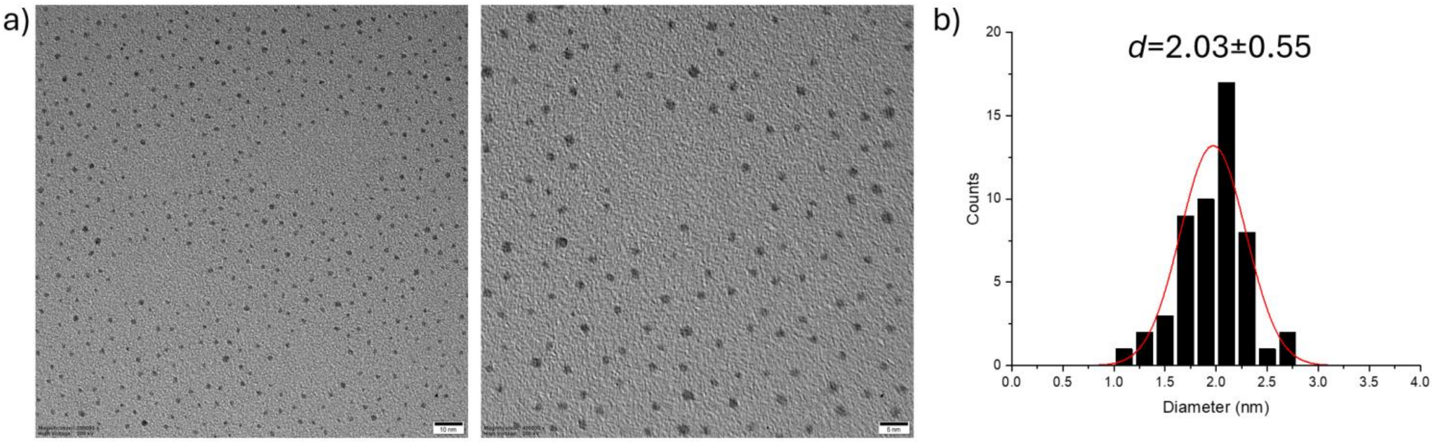

Figure S27: (a) High-resolution TEM image of ADA-NP nanoparticles. (b) Size distribution analysis of ADA-NP.

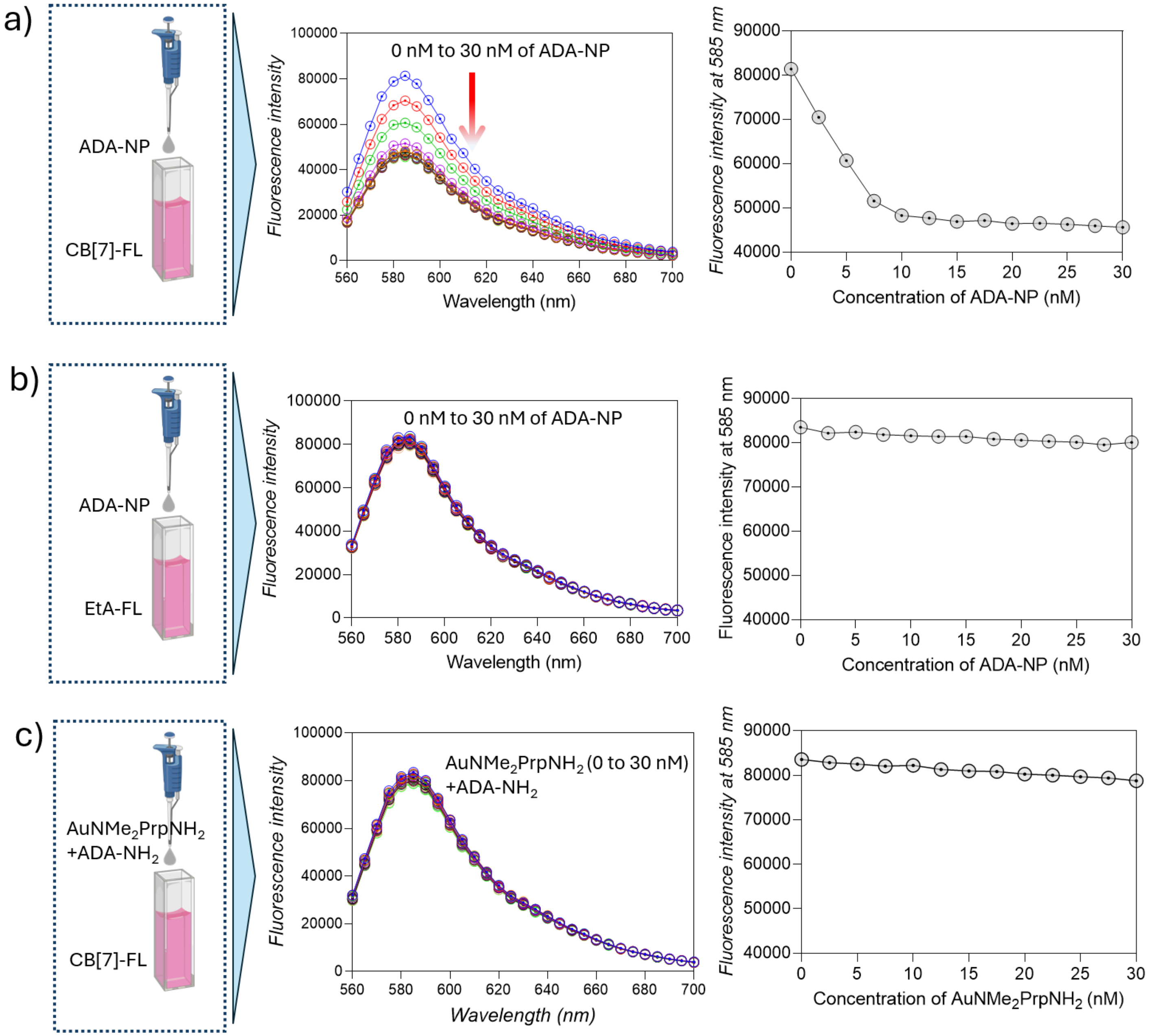

Figure S28. Quenching titration of CB[7]-TAMRA (500 nM) in PBS (a) and EtA-TAMRA (500 nM) (b) using ADA functionalized gold nanoparticles (c) Au-Prp-NH_2 gold nanoparticles without ADA functionalization.

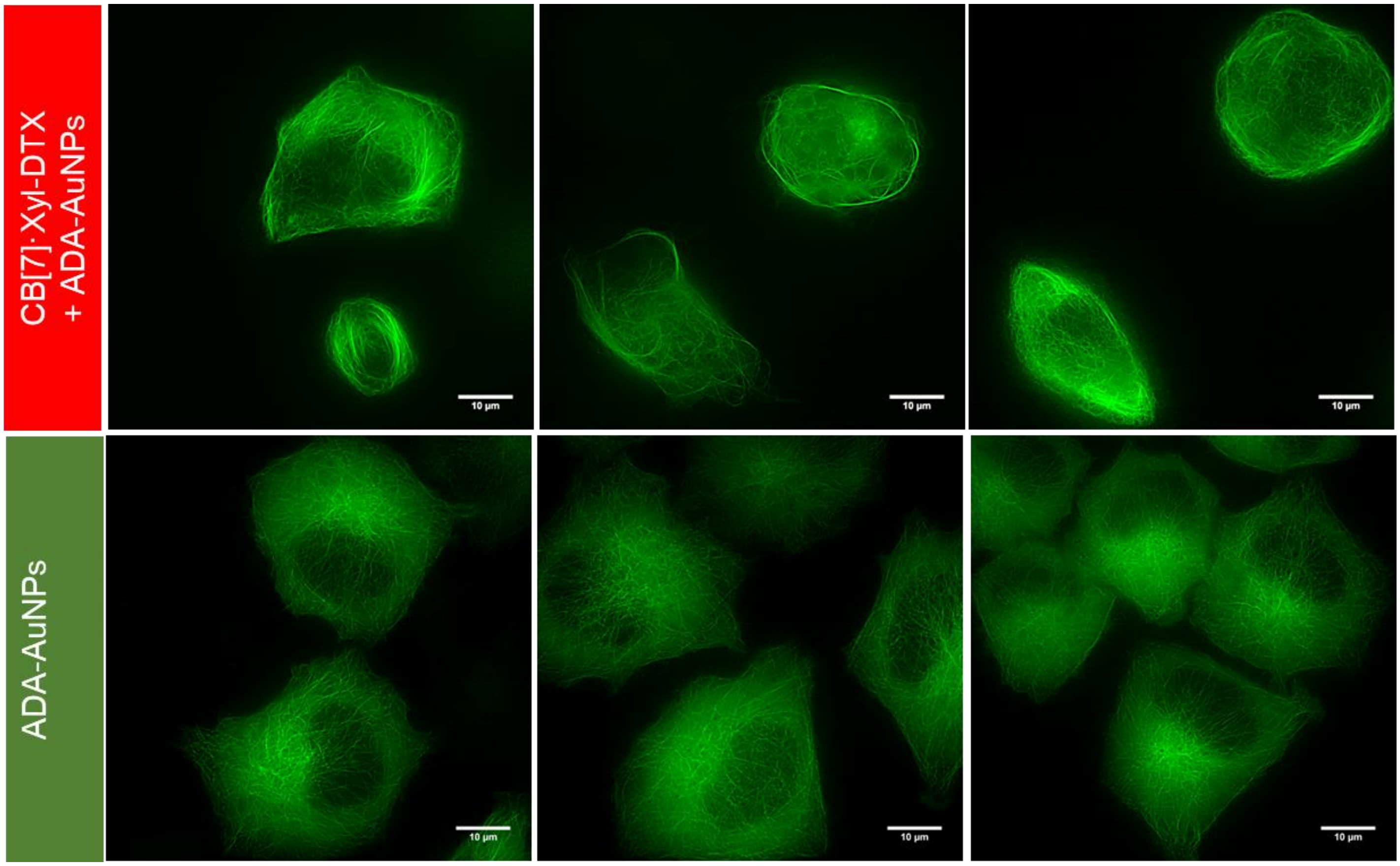

Figure S29: Cellular images of HeLa cells expressing GFP labelled *α-tubulin* treated with [CB[7]·Xyl-DTX + ADA-NPs] and ADA-NPs (top to down) after 23 h. Scale bar: 10 μm.

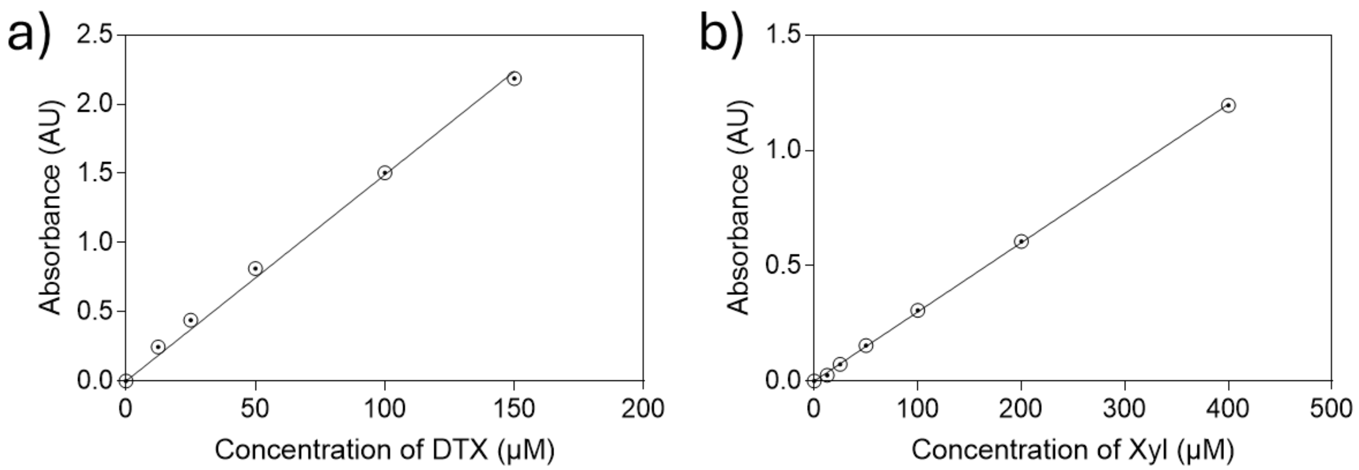

Figure S30: To calculate the molar extinction coefficient of DTX and Xyl. (a) Plot of absorbance *vs.* concentration of DTX (b) Plot of absorbance *vs.* concentration of Xyl.

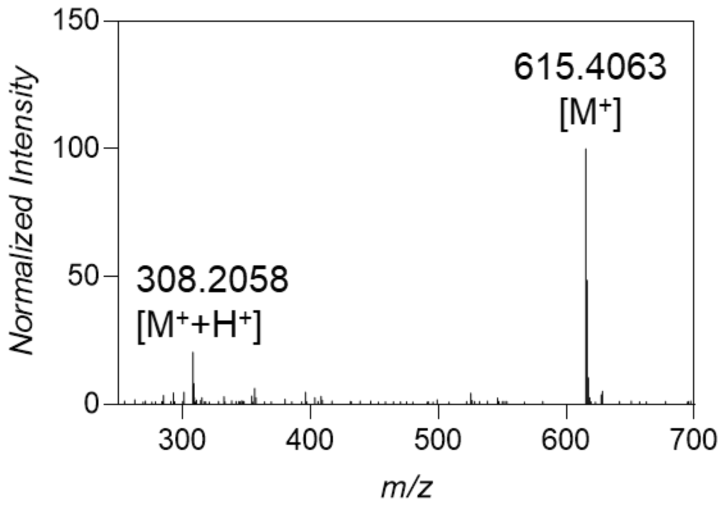

Figure S31: HRMS spectrum of Benz-Cy5.

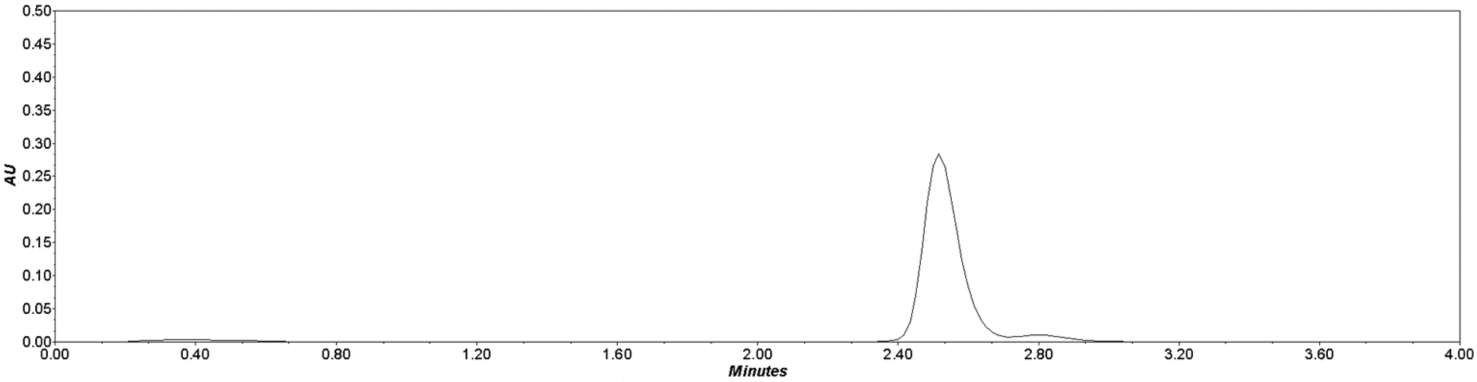

Figure S32: LC chromatogram of Benzyl conjugated Cy5 (Benz-Cy5). Water and acetonitrile with 0.1% formic acid were used as a mobile phase. The polarity of acetonitrile varied from 5% to 100% over the period of 4 min. The conjugated product Benz-Cy5 was eluted at 2.40 -2.66 min. The samples were prepared by mixing 20 µl milli-q water and 2 µl of Benzyl-Cy5 (from 245 µM stock in DMSO) and recorded the LC chromatogram at 646 nm wavelength absorption.

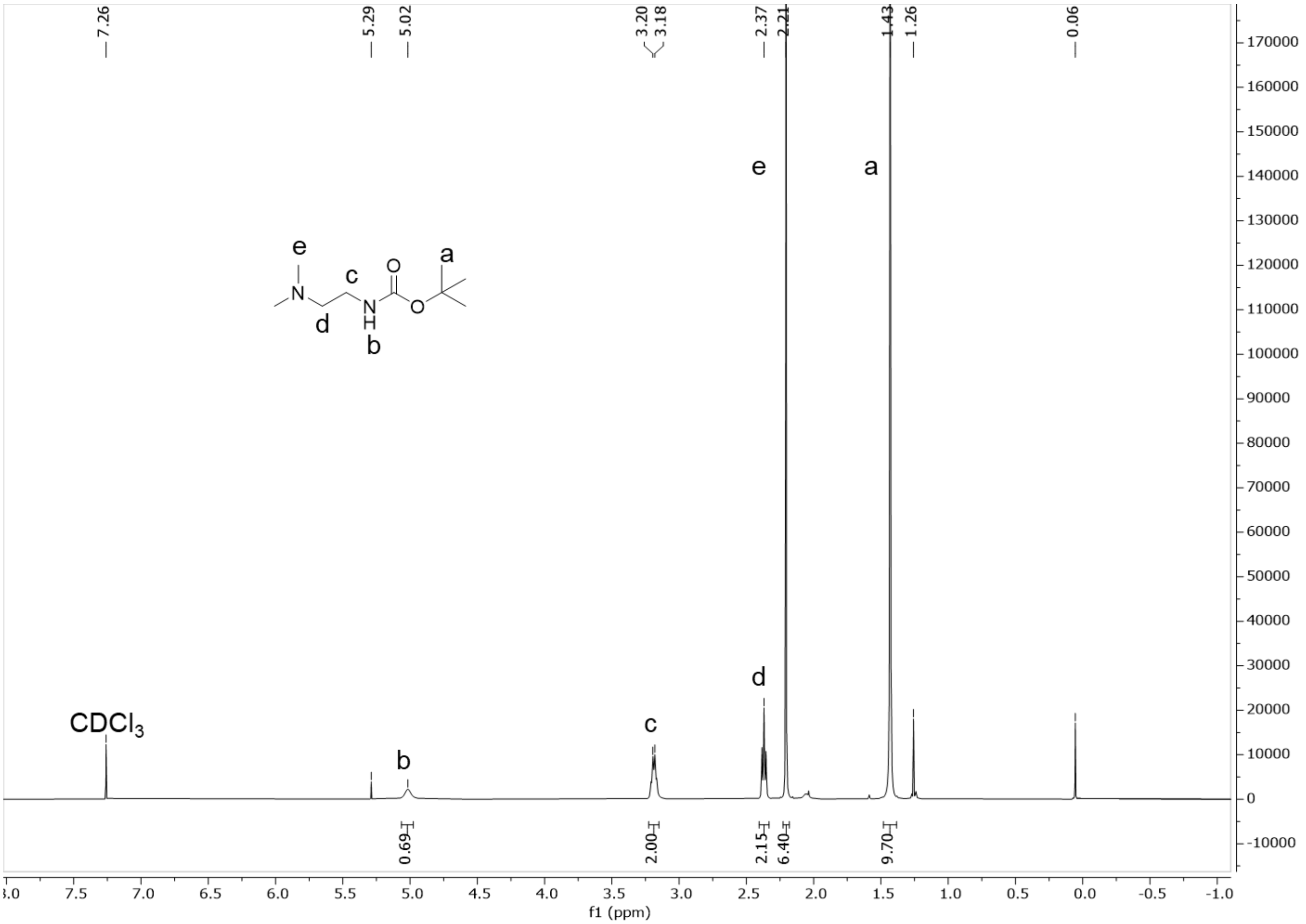

Figure S33: ^{1}H-NMR spectrum of tert-butyl 2-(dimethylamino) ethyl carbamate (400 MHz, $CDCl_3$).

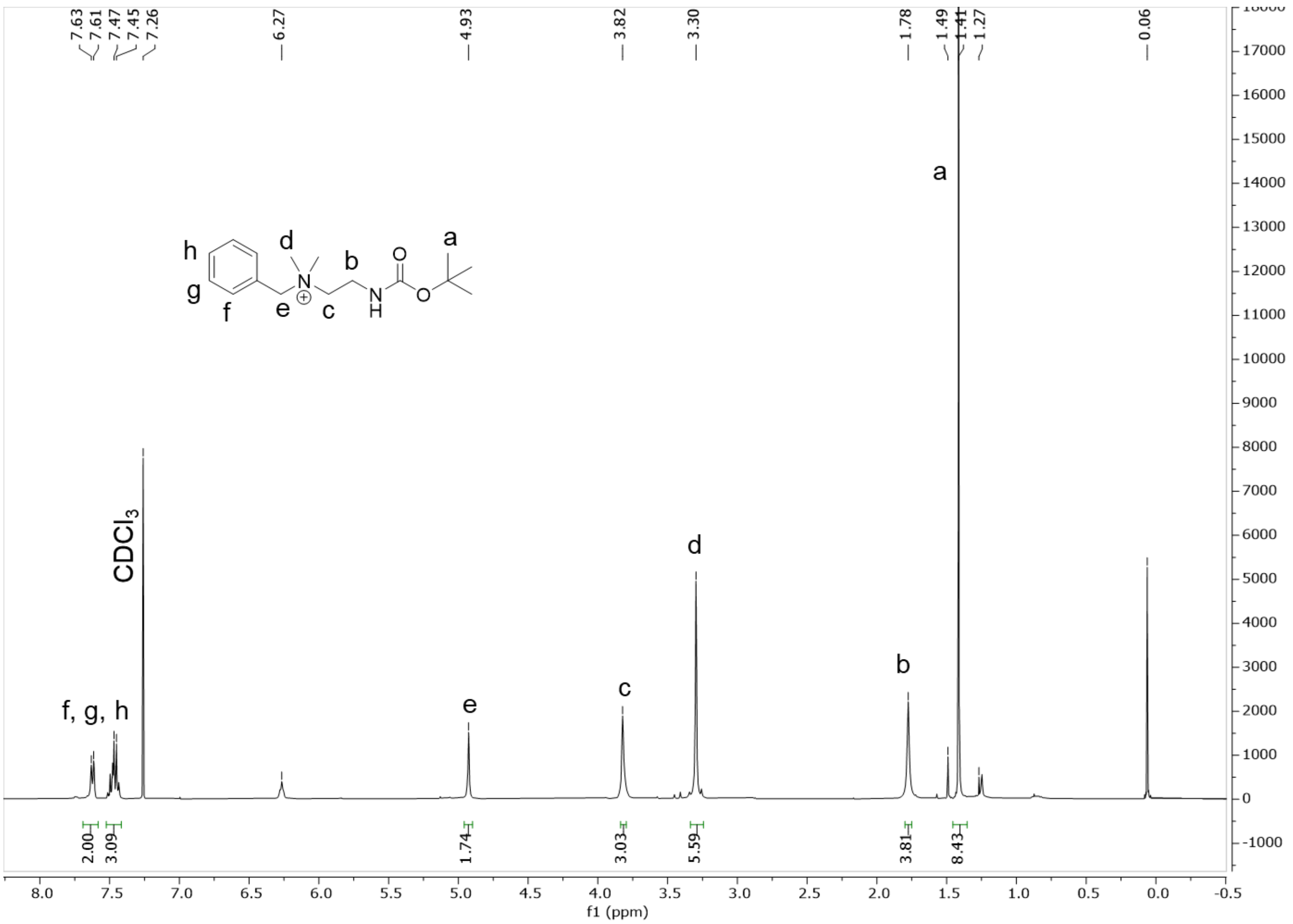

Supplementary S34: ^{1}H-NMR spectrum of Benzyl-NMe2-EDA-Boc (400 MHz, $CDCl_3$).

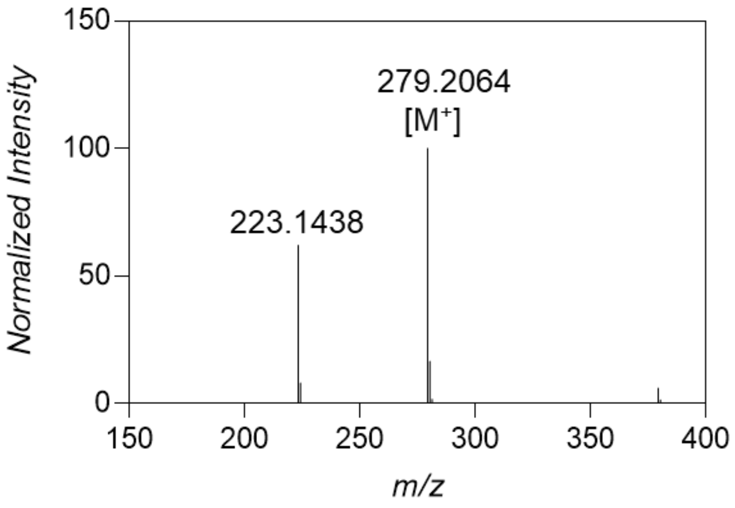

Supplementary S35: HRMS spectrum of Benzyl-NMe2-EDA-Boc.

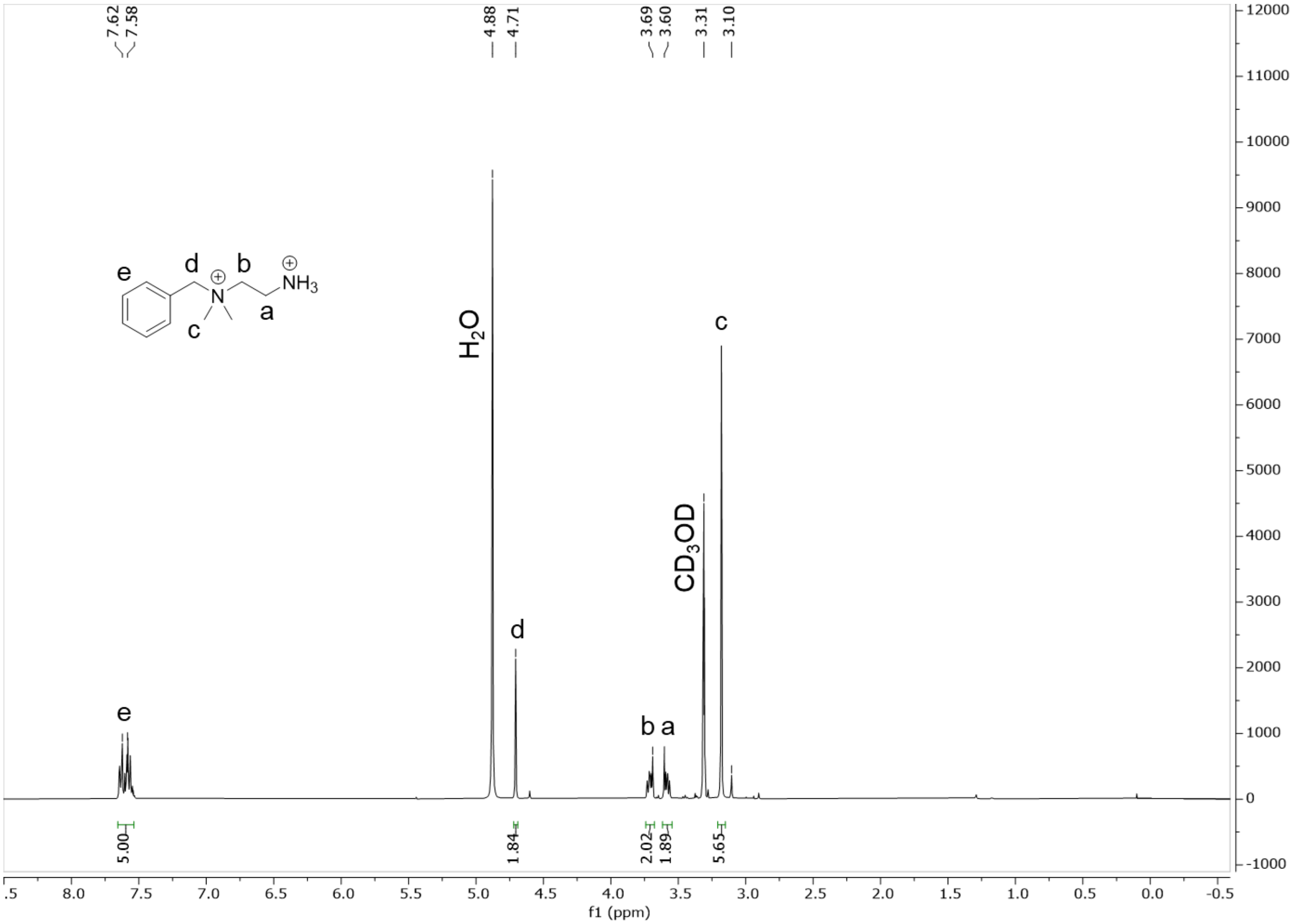

Supplementary S36: ^{1}H-NMR spectrum of Benzyl-NMe2-EDA (400 MHz, $CDCl_3$).

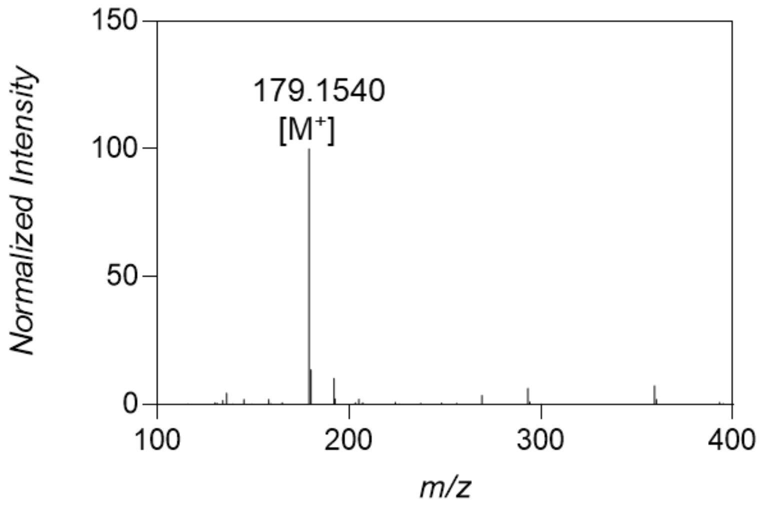

Supplementary S37: HRMS spectrum of Benzyl-NMe2-EDA.

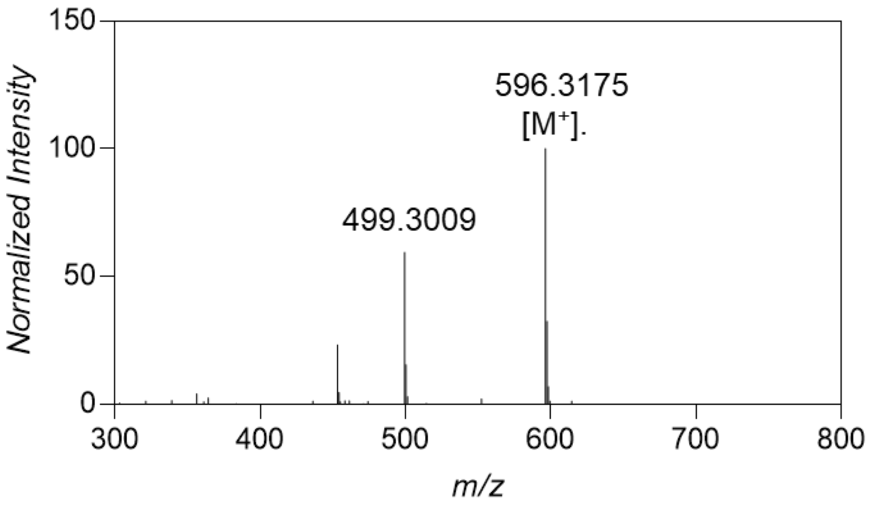

Supplementary S38: HRMS spectrum of Benzyl-NMe_2-EDA-PEG_5-NHS.

26. References:

1. Wang, J.; Wang, W.; Kollman, P. A.; Case, D. A. Antechamber: an accessory software package for molecular mechanical calculations. *J. Am. Chem. Soc.* **2001**, *222*, U403.

2. Kim, S.; Lee, J.; Jo, S.; Brooks III, C. L.; Lee, H. S.; Im, W. CHARMM-GUI ligand reader and modeler for CHARMM force field generation of small molecules. Wiley Online Library: **2017**.

3. Jorgensen, W. L.; Chandrasekhar, J.; Madura, J. D.; Impey, R. W.; Klein, M. L. Comparison of simple potential functions for simulating liquid water. *J. Chem. Phys.* **1983**, *79* (2), 926-935.

4. Lindahl, Abraham, Hess, van der Spoel, **2021**, DOI 10.5281/zenodo.4420785.

5. Löwe, J.; Li, H.; Downing, K.; Nogales, E. Refined structure of αβ-tubulin at 3.5 Å resolution. *J. Mol. Biol.* **2001**, *313* (5), 1045-1057.

6. Trott, O.; Vina, O. improving the speed and accuracy of docking with a new scoring function, efficient optimization and multithreading Oleg Public Access. *J. Comput. Chem.* **2010**, *31*, 455-461.

7. Barducci, A.; Bussi, G.; Parrinello, M. Well-tempered metadynamics: a smoothly converging and tunable free-energy method. *Phys. Rev. Lett.* **2008**, *100* (2), 020603.

8. Lindahl; Abraham; Hess; der Spoel, van. GROMACS 2019.6 Source Code. Zenodo February **2020**. https://doi.org/10.5281/zenodo.3685922

9. Tribello, G. A.; Bonomi, M.; Branduardi, D.; Camilloni, C.; Bussi, G. PLUMED 2: New feathers for an old bird. *Comput. Phys. Commun.* **2014**, *185* (2), 604-613.

10. Best, R. B.; Zhu, X.; Shim, J.; Lopes, P. E.; Mittal, J.; Feig, M.; MacKerell Jr, A. D. Optimization of the additive CHARMM all-atom protein force field targeting improved sampling of the backbone ϕ, ψ and side-chain $\chi 1$ and $\chi 2$ dihedral angles. Journal of chemical theory and computation **2012**, *8* (9), 3257-3273.

11. Evans, D. J.; Holian, B. L. The nose–hoover thermostat. *J. Chem. Theory Comput.* **1985**, *83* (8), 4069-4074.

12. Parrinello, M.; Rahman, A. Polymorphic transitions in single crystals: A new molecular dynamics method. *J. Appl. Phys.* **1981**, *52* (12), 7182-7190.

13. Adasme, M. F.; Linnemann, K. L.; Bolz, S. N.; Kaiser, F.; Salentin, S.; Haupt, V. J.; Schroeder, M. PLIP 2021: Expanding the scope of the protein–ligand interaction profiler to DNA and RNA. *Nucleic Acids Res.* **2021**, *49* (W1), W530-W534.

14. Das Saha, N.; Pradhan, S.; Sasmal, R.; Sarkar, A.; Berač, C. M.; Kölsch, J. C.; Pahwa, M.; Show, S.; Rozenholc, Y.; Topçu, Z.; Alessandrini, V.; Guibourdenche, J.; Tsatsaris, V.; Gagey-Eilstein, N.; Agasti, S. S. Cucurbit[7]uril Macrocyclic Sensors for Optical Fingerprinting: Predicting Protein Structural Changes to Identifying Disease-Specific Amyloid Assemblies. *J. Am. Chem. Soc.* **2022**, *144* (31), 14363-14379.

15. Som, A.; Pahwa, M.; Bawari, S.; Saha, N. D.; Sasmal, R.; Bosco, M. S.; Mondal, J.; Agasti, S. S. Multiplexed optical barcoding of cells via photochemical programming of bioorthogonal host–guest recognition. *Chem. Sci.* **2021**, *12* (15), 5484-5494.

16. Pahwa, M.; Jain, P.; Das Saha, N.; Narayana, C.; Agasti, S. S. Interfacial tetrazine click chemistry mediated assembly of multifunctional colloidosomes. *Chem. Commun.* **2021**, *57* (75), 9534-9537.
